# Immune-effector and infection-associated reporting patterns of CD20×CD3 bispecific antibodies in B-cell lymphoma: a label-anchored FAERS analysis with descriptive JADER contextualization

**DOI:** 10.3389/fimmu.2026.1920122

**Published:** 2026-10-02

**Authors:** Hao Zhong, Yongjian Liu, Yanduan Lin, Qi Yang, Guihao Hou

**Affiliations:** 1 Department of Pharmacy, The Third Affiliated Hospital of Sun Yat-sen University, Guangzhou, China; 2 Department of Pharmacy, Huizhou Central People’s Hospital, Huizhou, China

**Keywords:** CD20×CD3 bispecific antibody, cytokine release syndrome, FAERS, ICANS, JADER, opportunistic infection, pharmacovigilance, t-cell engager

## Abstract

**Background:**

CD20×CD3 bispecific antibodies are associated with acute immune-effector toxicities and infections, but drug-specific postmarketing co-reporting and report-level onset patterns remain incompletely characterized in B-cell lymphoma.

**Methods:**

We performed a label-anchored FAERS pharmacovigilance analysis with descriptive JADER contextualization. Deduplicated FAERS reports from 2022 Q4 through 2026 Q1 were restricted to primary-suspect epcoritamab, glofitamab, or mosunetuzumab reports in B-cell lymphoma, yielding 2,879 exposed reports. Prespecified MedDRA endpoints were evaluated against a disease-restricted comparator. Primary adjusted reporting odds ratios (aRORs) accounted for demographics, disease subgroup, reporting region, and calendar year. We mapped report-level phenotype intersections, summarized time to onset (TTO), and performed model-specification, restriction, comparator-exclusion, and TTO-completeness analyses.

**Results:**

The cohort included 1,463 epcoritamab, 944 glofitamab, and 472 mosunetuzumab reports. CRS had higher primary adjusted reporting odds for epcoritamab (aROR 4.70, 95% CI 4.15 to 5.32), glofitamab (3.43, 2.94 to 4.00), and mosunetuzumab (6.23, 4.97 to 7.81). ICANS had higher reporting odds for epcoritamab (2.28, 1.87 to 2.80); the glofitamab estimate crossed 1 in the primary model (1.26, 0.93 to 1.70) and varied with covariate specification, while mosunetuzumab ICANS was sparse. Epcoritamab had higher reporting odds for CMV (4.54, 3.66 to 5.62) and narrow opportunistic infection (2.20, 1.84 to 2.63). Mosunetuzumab had higher reporting odds for pneumonia/lower respiratory infection (1.74, 1.32 to 2.31) and COVID-19 (1.66, 1.25 to 2.21), although these infection-specific patterns attenuated in some geographic and calendar restrictions. Immune-effector and infectious phenotypes co-occurred in reports containing serious outcomes. TTO was calculable for 33.7%–61.2% of reports and suggested earlier CRS than infection-associated reporting, but report characteristics differed by TTO completeness.

**Conclusions:**

CRS was the clearest shared reporting pattern across the three CD20×CD3 antibodies. Neurotoxicity and infection-associated findings were more drug- and analysis-dependent. Report-level co-reporting supports concurrent consideration of inflammatory toxicity and infection when presentations overlap, while later infection-associated TTO distributions support continued awareness beyond step-up dosing. Spontaneous-reporting data cannot estimate incidence, causality, or comparative treatment risk.

## Introduction

T-cell-engaging bispecific antibodies redirect cytotoxic T cells toward tumor-associated antigens and have become an established therapeutic platform in hematologic malignancies (1, 2). In B-cell lymphoma, the CD20×CD3 antibodies epcoritamab, glofitamab, and mosunetuzumab have produced clinically meaningful responses after prior therapy and are increasingly being incorporated into broader treatment strategies (3–8). Their off-the-shelf availability and distinct dosing schedules offer practical advantages, but sustained T-cell engagement creates a toxicity profile that extends beyond isolated acute adverse-event terms.

Current US prescribing information identifies cytokine release syndrome (CRS) as a central toxicity for all three agents. Neurologic toxicity, including immune effector cell-associated neurotoxicity syndrome (ICANS), and serious infections are also recognized, with drug-specific differences in boxed warnings, dosing, and clinical context (9–11). Expert consensus therefore emphasizes step-up dosing, close observation for CRS and ICANS, infection assessment, and individualized prophylaxis (12). However, trial populations, follow-up duration, disease composition, and management practices differ across agents and indications, limiting indirect comparisons.

Early CD3-mediated T-cell activation can manifest as CRS, ICANS, or HLH-like inflammatory syndromes. B-cell depletion, hypogammaglobulinaemia, cytopenias, prior immunochemotherapy, corticosteroid exposure, and lymphoma-related immune dysfunction may contribute to infection susceptibility (12–16). Fever, hypotension, hypoxia, encephalopathy, cytopenias, and inflammatory-marker elevation may occur in both immune-effector toxicity and infection. Evaluating these domains separately may therefore miss clinically relevant report-level co-reporting.

ASTCT consensus criteria provide a common framework for grading CRS and ICANS, while subsequent consensus work has distinguished immune effector cell-associated HLH-like syndromes from conventional CRS (17, 18). These frameworks improve clinical recognition, but spontaneous reports rarely contain the granular examination, laboratory, and treatment-response data required for formal adjudication.

Spontaneous-report systems cannot estimate incidence, but they can identify reporting patterns across large, heterogeneous postmarketing populations and characterize uncommon or co-reported phenotypes (19, 20). Previous bispecific-antibody pharmacovigilance analyses have largely emphasized class-wide or preferred-term signal screening, multi-database comparisons, or organ-specific events (21–23). The incremental focus here was a lymphoma-restricted comparator, label-informed prespecified domains, explicit report-level co-reporting, TTO completeness assessment, and descriptive contextualization in a second reporting system. These design features address indication leakage and comparator heterogeneity, but they do not overcome the inherent limitations of spontaneous reports.

We used a label-anchored FAERS pharmacovigilance framework, supplemented by descriptive JADER contextualization, to characterize immune-effector and infection-associated reporting patterns of epcoritamab, glofitamab, and mosunetuzumab in B-cell lymphoma. The objectives were to estimate drug-specific reporting odds for prespecified domains; characterize report-level co-reporting and reported clinical consequences; describe TTO among reports with calculable dates; and evaluate sensitivity to model specification, disease composition, reporting geography, calendar period, comparator construction, and TTO completeness.

## Methods

### Study design and reporting framework

This retrospective pharmacovigilance study used publicly available, deidentified individual case safety reports. The analytic framework was finalized before production of the main figures and was anchored to current FDA prescribing information for epcoritamab, glofitamab, and mosunetuzumab (9–11).

Endpoint families were derived from FDA prescribing information and organized into immune-effector toxicities, infectious toxicities, and immune-suppression context markers, as summarized in **Supplementary Figures S1A, B**. The label-informed working hypotheses used to guide drug-specific phenotyping are shown in **Supplementary Figure S1C**. Endpoint categories and analysis tiers are provided in **Supplementary Table S1**.

FAERS was the primary analytic database. JADER, whose structure and data quality have been independently characterized, provided descriptive cross-database contextualization rather than formal validation, replication, or pooled cross-database disproportionality analysis (24, 25).

### FAERS data source and report deduplication

Quarterly FAERS data files were obtained from the FDA public dashboard and data download service (24). The study period extended from 2022 Q4, corresponding to the first US approval within the study class, through 2026 Q1. Drug, reaction, indication, demographic, outcome, and therapy files were linked using PRIMARYID, CASEID, and drug sequence identifiers.

Reports were deduplicated according to FDA conventions by retaining the most recent version for a case, using case and receipt-date fields and, where needed, the highest primary identifier. The resulting source contained 4,967,690 deduplicated reports. Initial FDA receipt date (INIT_FDA_DT) was used for reporting-trend analyses of overall drug reports and prespecified endpoints (**Supplementary Figures S3A, B**), while demographic, reaction, outcome, indication, and therapy information was taken from the retained latest version. Withdrawn or structurally invalid records were excluded during data preparation. The complete cohort workflow and quality-control audit are provided in **Supplementary Figure S2**.

### Drug exposure and B-cell lymphoma cohort

Generic names, brand names, spelling variants, and product-specific aliases were mapped to epcoritamab, glofitamab, or mosunetuzumab using a prespecified drug dictionary. Indication terms were then restricted to diffuse large B-cell lymphoma, high-grade or other large B-cell lymphoma, follicular lymphoma, and other or unspecified B-cell non-Hodgkin lymphoma categories (**Supplementary Table S2B**), yielding a final exposed cohort of 2,879 reports (**Figure 1A**).

**Figure 1:**
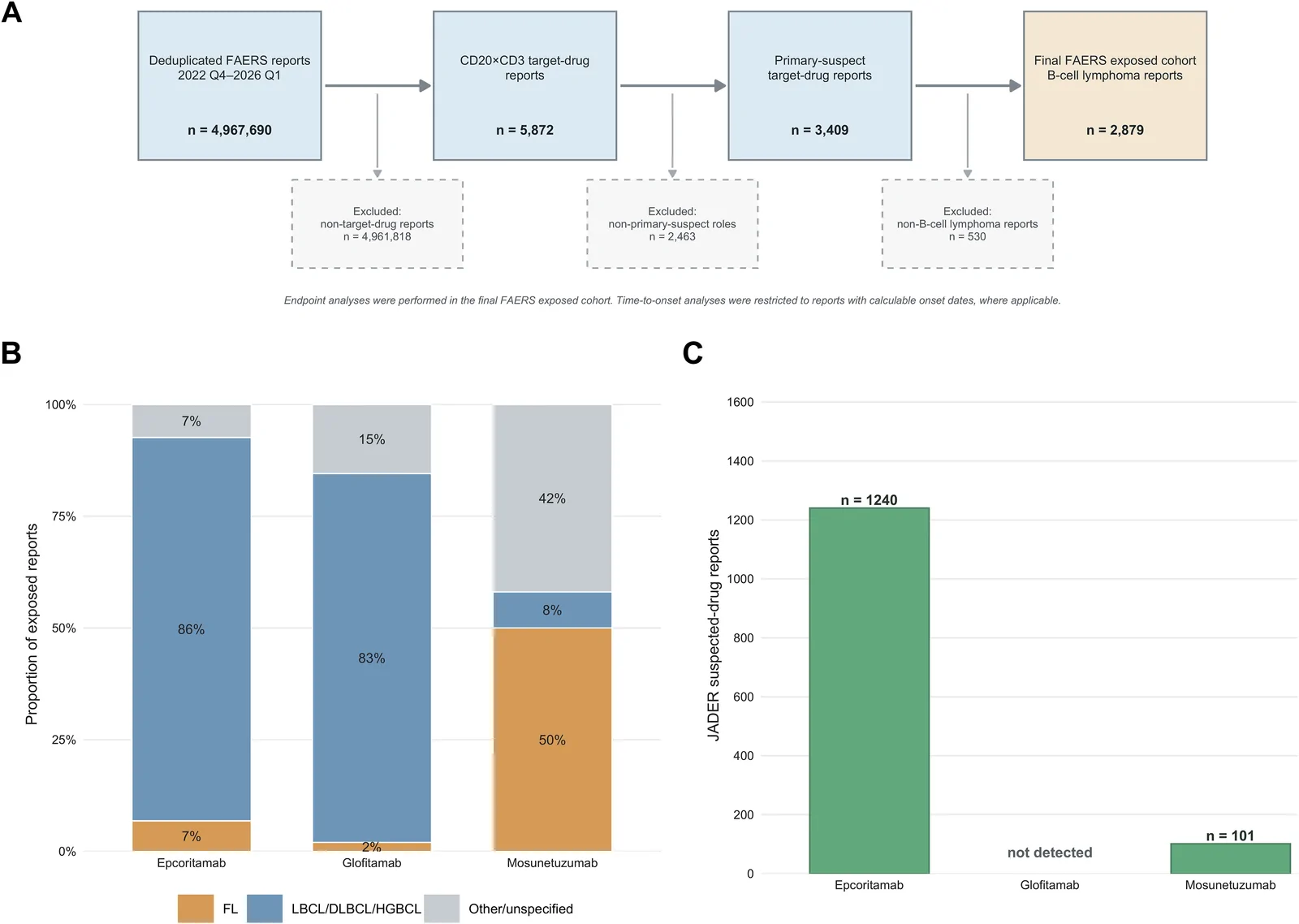
Cohort assembly and case landscape for FAERS analysis and descriptive JADER contextualization. **(A)** Simplified FAERS cohort construction from deduplicated reports to CD20×CD3 target-drug reports, primary-suspect reports, and the final B-cell lymphoma-exposed cohort. Endpoint analyses used the final FAERS exposed cohort; TTO analyses were restricted to reports with calculable dates. **(B)** Distribution of exposed FAERS reports across LBCL/DLBCL/high-grade B-cell lymphoma, follicular lymphoma, and other or unspecified B-cell lymphoma/NHL subgroups. **(C)** Suspected-drug report availability in JADER. Epcoritamab and mosunetuzumab were evaluable, whereas glofitamab was not detected using the prespecified dictionary.

For disproportionality analyses, the comparator was the full set of 63,544 deduplicated FAERS reports coded for B-cell lymphoma, including the 2,879 exposed reports. This disease-restricted population compared each target drug with other reports arising in a more similar indication context and reduced, but could not eliminate, confounding by disease composition. Reports listing more than one study CD20×CD3 antibody as a primary suspect were excluded from regression models to preserve drug-specific exposure assignment. RORs quantify relative reporting odds in spontaneous-reporting data; they do not estimate incidence, causality, or comparative clinical risk. A sensitivity analysis excluded all primary-suspect study-drug reports from the comparator. Crude RORs obtained using the full FAERS database and the B-cell lymphoma comparator are presented in **Supplementary Table S4**.

### Label-anchored endpoint definitions

Adverse events were coded using MedDRA version 28.1. Clinical domains were defined with individual preferred terms (PTs), narrow Standardised MedDRA Queries (SMQs), and prespecified curated PT groups (26). Endpoint categories and analysis tiers are summarized in **Supplementary Table S1**, and complete PT/SMQ mappings are provided in **Supplementary Table S3**.

The study was designed and reported with reference to the READUS-PV recommendations for disproportionality analyses and the STROBE statement (27–29).

The primary clinical domains were CRS, ICANS, and the infection composite. Secondary and contextual domains included broadly defined neurotoxicity, serious-infection proxy, pneumonia/lower respiratory infection, COVID-19, cytomegalovirus (CMV), herpes simplex or varicella-zoster virus infection, fungal infection, Pneumocystis jirovecii pneumonia (PJP), progressive multifocal leukoencephalopathy (PML), opportunistic infection, hypogammaglobulinaemia, cytopenia-related markers, and HLH/MAS.

The immune-effector domain included CRS, ICANS, broadly defined neurotoxicity, and hemophagocytic lymphohistiocytosis/macrophage activation syndrome (HLH/MAS). CRS and ICANS used core PT definitions. Broadly defined neurotoxicity combined ICANS with curated neurologic PTs and narrow encephalopathy or delirium terms. HLH/MAS was analyzed as a rare contextual phenotype and was not treated as interchangeable with CRS or ICANS (17, 18).

The infectious domain included an infection composite, a serious-infection proxy, pneumonia/lower respiratory infection, sepsis, COVID-19, CMV, herpes simplex or varicella-zoster virus infection, fungal infection, PJP, PML, and opportunistic-infection groupings. COVID-19 vaccination, screening, prophylaxis, and treatment terms were excluded. The serious-infection proxy incorporated pneumonia, sepsis, narrow opportunistic infection, CMV, fungal infection, PJP, and PML. Hypogammaglobulinaemia and cytopenia-related endpoints were immune-suppression context markers rather than infection events.

Exploratory PT-level analyses were secondary to the clinically prespecified domain analyses. Indication terms, refractory lymphoma terms, disease progression, therapeutic procedures, product-use terms, and other prespecified non-adverse-event concepts were removed from exploratory PT-level displays. This cleaning step was applied separately to FAERS and JADER.

### Clinical outcomes and report-level phenotype co-reporting

Reported outcomes included death, hospitalization, life-threatening events, disability, congenital anomaly, and other serious outcomes. A composite severe outcome was defined as any serious outcome recorded in FAERS. These fields describe report seriousness and do not establish that the study drug or selected phenotype caused the outcome.

At the report level, mutually exclusive core phenotypes were constructed from Boolean indicators for CRS, ICANS, and the infection composite: CRS only, ICANS only, infection only, CRS plus ICANS, CRS plus infection, ICANS plus infection, and CRS plus ICANS plus infection. HLH plus infection and narrow opportunistic infection were retained as non-mutually exclusive contextual phenotypes. For each phenotype, we summarized drug-specific prevalence and the proportions of reports containing death, hospitalization, or a severe outcome. These intersections represent co-reporting within the same case record and do not establish temporal, biological, or causal coupling.

### Time-to-onset analysis

TTO was calculated as the difference between the complete eight-digit report-level event date in the demographic file and the start date of the corresponding target drug in the therapy file. Only dates linked to the target-drug sequence were used. Values from 0 to 3,650 days were retained; negative values and reports without complete calculable dates were excluded. TTO was summarized by drug and endpoint using medians, interquartile ranges (IQRs), and four windows: 0–7, 8–30, 31–90, and more than 90 days. TTO reflected the interval between target-drug start date and the report-level event date among reports containing the endpoint; it was not adjudicated adverse-event-level onset and did not identify the dose most proximal to the event. No formal between-drug comparison of TTO was performed. Characteristics of reports with and without calculable TTO were compared using counts, percentages, and standardized differences.

### Disproportionality and statistical analysis

For each drug-endpoint pair, the crude ROR and 95% CI were calculated from a 2×2 table with a Haldane-Anscombe 0.5 correction applied to all cells (30, 31). Primary aRORs were estimated using separate logistic regression models in the B-cell lymphoma comparator cohort. The exposure was the specific study drug coded as primary suspect. The primary model adjusted for age group (<65, 65–74, ≥75, or unknown), sex, reporting region, B-cell lymphoma subgroup, and report year. These variables were prioritized because they precede or contextualize reporting more plausibly than treatment and management indicators. Reference categories were age <65 years, female sex, US reporting region, aggressive B-cell lymphoma, and report year 2022. A small ridge term was used for numerical stability; model convergence was recorded for every estimate.

All eligible reports were included; no sample-size calculation or sampling was performed. Missing age, sex, and region were retained as explicit categories, and no statistical imputation was used.

RORs quantify relative reporting odds, not incidence, causality, severity, or comparative treatment risk. Estimates with 95% CIs excluding 1 were described as higher or lower reporting odds, but interpretation prioritized effect size, precision, clinical coherence, and consistency across analyses. No formal multiplicity-adjusted confirmatory testing was performed because the domain analyses were prespecified and pharmacovigilance signals were treated as hypothesis-generating. Complete crude and primary adjusted domain outputs for the B-cell lymphoma comparator are provided in **Supplementary Table S5**.

Model-specification sensitivity analyses compared the primary model with an extended treatment-context model that additionally included report-level indicators for CAR T-cell therapy, Bruton tyrosine kinase inhibitor, anti-CD20 therapy, chemotherapy, and corticosteroid use, plus reduced extended models excluding corticosteroid, anti-CD20 therapy, or both. These indicators may represent prior therapy, regimen context, concomitant exposure, or post-event management and were therefore not treated as baseline confounders in the primary model. Restriction analyses assessed disease composition, reporting geography, and calendar effects using LBCL/DLBCL/HGBCL reports, non-FL reports, US-only reports, and a common 2024Q2-2026Q1 period. Comparator-exclusion analyses repeated crude, primary, and extended estimates after removing all primary-suspect study-drug reports from the B-cell lymphoma comparator. Adjusted models were fitted where data permitted; otherwise, crude estimates or descriptive counts were retained.

Exploratory PT-level analyses required at least three exposed reports. A conservative sensitivity definition required simultaneous positivity for four signal-detection criteria: lower 95% CI of the ROR greater than 1; proportional reporting ratio at least 2 with chi-square at least 4; lower 95% credibility limit of the information component (IC025) greater than 0; and lower empirical Bayes geometric mean bound (EB05) greater than 2 (30–33). These PT screens were secondary to the clinically prespecified domain analysis.

Analyses and figures were produced in R version 4.6.0 and cross-checked against independently exported source tables. All displayed cohort arithmetic, endpoint counts, regression estimates, phenotype intersections, and TTO summaries underwent programmed cross-artifact checks.

### Descriptive JADER contextualization

JADER data were obtained from the Pharmaceuticals and Medical Devices Agency public download (see Data availability statement). The downloaded JADER release covered reports from 2004 Q1 through 2025 Q4 and was accessed on 30 May 2026. The same target-drug dictionary (**Supplementary Table S2A**) and cleaned endpoint framework were applied where compatible with JADER structure. From 1,032,027 source reports, 2,691 target-drug matches were identified; retaining the latest suspected-drug version yielded 1,341 reports for descriptive analysis. Because JADER differs from FAERS in data structure, market availability, reporting practice, and available covariates, it was used only as an independent descriptive reporting environment. No pooled analysis, adjusted JADER disproportionality model, or formal cross-database statistical comparison was performed; JADER therefore could not validate FAERS signals.

## Results

### Cohort characteristics

The FAERS workflow identified 5,872 unique CD20×CD3 target-drug reports from 4,967,690 deduplicated reports. Restriction to primary-suspect exposure and B-cell lymphoma produced 2,879 reports: 1,463 (50.8%) for epcoritamab, 944 (32.8%) for glofitamab, and 472 (16.4%) for mosunetuzumab (**Figure 1A**). Epcoritamab and glofitamab reports predominantly involved LBCL/DLBCL/HGBCL, whereas follicular lymphoma accounted for 50.0% of mosunetuzumab reports (**Table 1** and **Figure 1B**). Age was unknown in 17.2%, 26.4%, and 21.8% of reports, respectively; among all reports, age 75 years or older was recorded in 37.5%, 15.3%, and 22.9%. Male sex was more frequently reported than female sex for each agent.

**Table 1:** Characteristics of FAERS primary-suspect CD20×CD3 bispecific antibody reports.

Characteristic	Epcoritamab (N = 1,463)	Glofitamab (N = 944)	Mosunetuzumab (N = 472)
Disease subgroup, n (%)			
LBCL/DLBCL/HGBCL	1,255 (85.8%)	779 (82.5%)	38 (8.1%)
FL	100 (6.8%)	19 (2.0%)	236 (50.0%)
Other/unspecified B-cell lymphoma	108 (7.4%)	146 (15.5%)	198 (41.9%)
Age group, n (%)			
<65 years	306 (20.9%)	341 (36.1%)	154 (32.6%)
65–74 years	357 (24.4%)	210 (22.2%)	107 (22.7%)
≥75 years	548 (37.5%)	144 (15.3%)	108 (22.9%)
Unknown	252 (17.2%)	249 (26.4%)	103 (21.8%)
Sex, n (%)			
Female	539 (36.8%)	322 (34.1%)	195 (41.3%)
Male	786 (53.7%)	504 (53.4%)	226 (47.9%)
Unknown	138 (9.4%)	118 (12.5%)	51 (10.8%)
Reporting region, n (%)			
US	175 (12.0%)	122 (12.9%)	193 (40.9%)
Non-US	1,288 (88.0%)	822 (87.1%)	279 (59.1%)
Outcome, n (%)			
Death	551 (37.7%)	334 (35.4%)	58 (12.3%)
Hospitalization	443 (30.3%)	276 (29.2%)	282 (59.7%)
Life-threatening	92 (6.3%)	42 (4.4%)	18 (3.8%)
Severe outcome	893 (61.0%)	589 (62.4%)	312 (66.1%)
Concomitant therapy, n (%)			
CAR-T	6 (0.4%)	8 (0.8%)	0 (0.0%)
BTK inhibitor	8 (0.5%)	13 (1.4%)	7 (1.5%)
Anti-CD20	86 (5.9%)	354 (37.5%)	30 (6.4%)
Chemotherapy	103 (7.0%)	167 (17.7%)	43 (9.1%)
Corticosteroid	478 (32.7%)	178 (18.9%)	205 (43.4%)

Data are n (%) unless otherwise indicated; percentages use the drug-specific number of reports as the denominator. Outcome and concomitant-therapy categories may overlap. LBCL, large B-cell lymphoma; DLBCL, diffuse large B-cell lymphoma; HGBCL, high-grade B-cell lymphoma; FL, follicular lymphoma; CAR-T, chimeric antigen receptor T-cell therapy; BTK, Bruton tyrosine kinase.

Death was recorded in 551 epcoritamab (37.7%), 334 glofitamab (35.4%), and 58 mosunetuzumab (12.3%) reports. Hospitalization was recorded in 443 (30.3%), 276 (29.2%), and 282 (59.7%), respectively. These unadjusted proportions reflect different reporting and indication contexts and were not used to rank drug safety. Selected treatment-context indicators also differed, most notably anti-CD20 co-reporting, which was present in 86 epcoritamab (5.9%), 354 glofitamab (37.5%), and 30 mosunetuzumab (6.4%) reports.

### Shared CRS phenotype and differential neurotoxicity reporting

CRS was the dominant immune-effector phenotype for all three agents (**Figures 2A, B**, **Table 2**). It was reported in 459 epcoritamab (31.4%), 253 glofitamab (26.8%), and 110 mosunetuzumab (23.3%) reports. Relative to other B-cell lymphoma reports, primary adjusted reporting odds were higher for epcoritamab (aROR 4.70, 95% CI 4.15 to 5.32), glofitamab (3.43, 2.94 to 4.00), and mosunetuzumab (6.23, 4.97 to 7.81).

**Figure 2:**
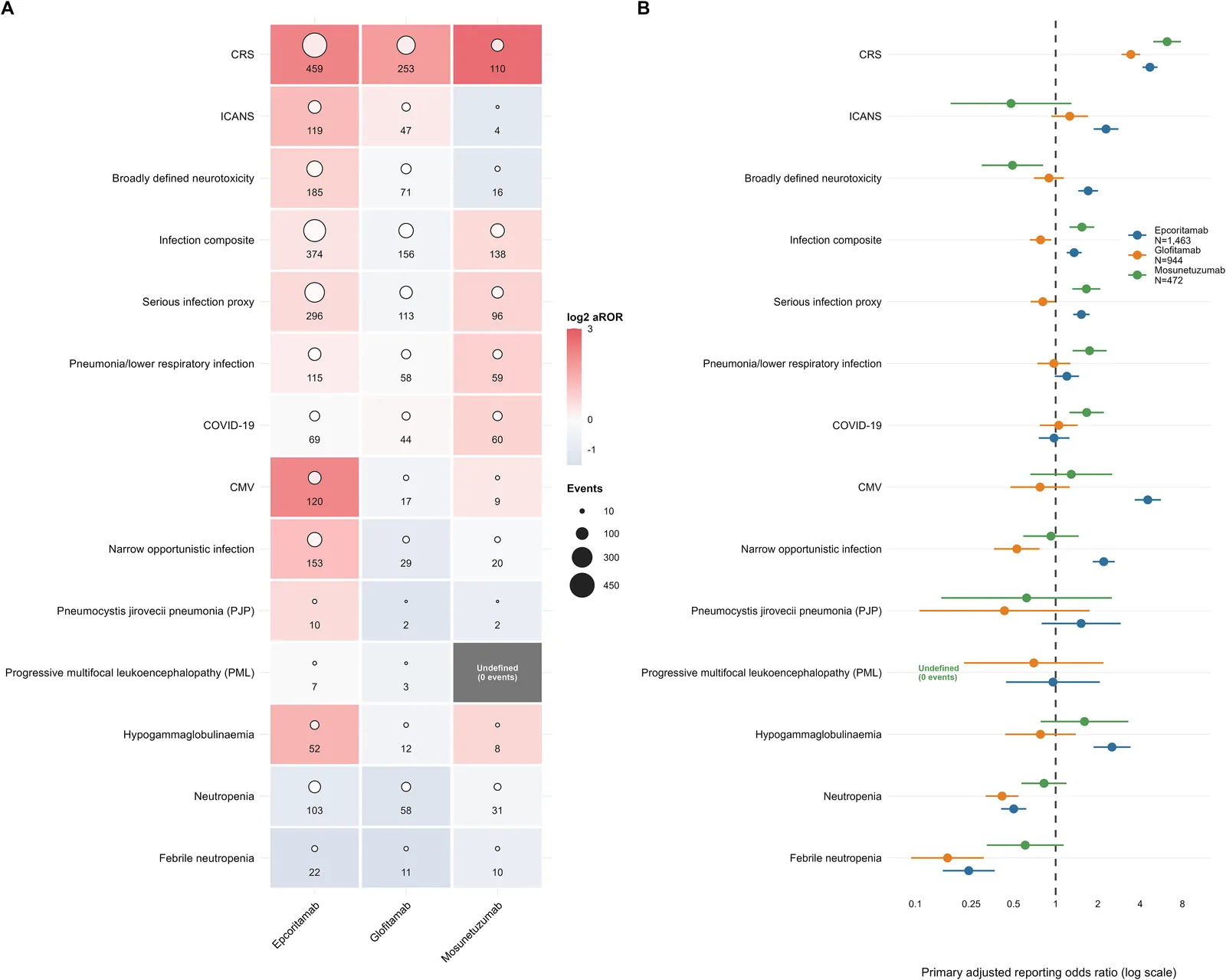
Primary immune-effector and infection-associated reporting patterns. **(A)** Heatmap of primary adjusted reporting odds ratios (aRORs) for label-informed toxicity domains among primary-suspect CD20×CD3 bispecific-antibody reports in the B-cell lymphoma-restricted FAERS cohort. Tile color represents log_2_ aROR (red, higher reporting odds; blue, lower reporting odds; white, aROR=1); circle area represents the number of exposed reports containing the endpoint, with the event count printed directly beneath each circle. Grey cells and text denote undefined adjusted estimates when no exposed event was reported. **(B)** Forest plot of the same primary aRORs and 95% CIs on a logarithmic scale. The primary model adjusted for age group, sex, reporting region, disease subgroup, and report year. The vertical dashed line indicates an aROR of 1.

**Table 2:** Selected primary and key secondary disproportionality results.

Endpoint	Events, n (%)	ROR (95% CI)	Primary aROR (95% CI)
Epcoritamab (N = 1,463)			
CRS	459 (31.4%)	6.28 (5.60-7.05)	4.70 (4.15-5.32)
ICANS	119 (8.1%)	2.99 (2.47-3.63)	2.28 (1.87-2.80)
Broadly defined neurotoxicity	185 (12.6%)	1.73 (1.48-2.02)	1.70 (1.45-2.01)
Infection composite	374 (25.6%)	1.44 (1.28-1.62)	1.35 (1.20-1.53)
Serious infection proxy	296 (20.2%)	1.70 (1.49-1.93)	1.52 (1.33-1.74)
Pneumonia/lower respiratory infection	115 (7.9%)	1.29 (1.06-1.56)	1.20 (0.99-1.47)
COVID-19	69 (4.7%)	0.85 (0.67-1.09)	0.97 (0.76-1.25)
CMV	120 (8.2%)	5.81 (4.77-7.07)	4.54 (3.66-5.62)
Narrow opportunistic infection	153 (10.5%)	2.36 (1.99-2.80)	2.20 (1.84-2.63)
Pneumocystis jirovecii pneumonia (PJP)	10 (0.7%)	1.44 (0.78-2.66)	1.52 (0.79-2.91)
Progressive multifocal leukoencephalopathy (PML)	7 (0.5%)	1.10 (0.53-2.27)	0.96 (0.44-2.06)
Glofitamab (N = 944)			
CRS	253 (26.8%)	4.83 (4.17-5.59)	3.43 (2.94-4.00)
ICANS	47 (5.0%)	1.72 (1.28-2.32)	1.26 (0.93-1.70)
Broadly defined neurotoxicity	71 (7.5%)	0.96 (0.75-1.22)	0.90 (0.70-1.15)
Infection composite	156 (16.5%)	0.82 (0.69-0.98)	0.78 (0.65-0.93)
Serious infection proxy	113 (12.0%)	0.90 (0.74-1.09)	0.81 (0.66-0.99)
Pneumonia/lower respiratory infection	58 (6.1%)	0.99 (0.76-1.29)	0.97 (0.74-1.27)
COVID-19	44 (4.7%)	0.85 (0.63-1.15)	1.05 (0.77-1.43)
CMV	17 (1.8%)	1.11 (0.69-1.79)	0.77 (0.48-1.26)
Narrow opportunistic infection	29 (3.1%)	0.63 (0.43-0.90)	0.53 (0.36-0.77)
Pneumocystis jirovecii pneumonia (PJP)	2 (0.2%)	0.52 (0.15-1.80)	0.43 (0.11-1.74)
Progressive multifocal leukoencephalopathy (PML)	3 (0.3%)	0.79 (0.27-2.26)	0.70 (0.22-2.20)
Mosunetuzumab (N = 472)			
CRS	110 (23.3%)	3.91 (3.15-4.85)	6.23 (4.97-7.81)
ICANS	4 (0.8%)	0.31 (0.12-0.78)	0.48 (0.18-1.29)
Broadly defined neurotoxicity	16 (3.4%)	0.42 (0.26-0.69)	0.49 (0.30-0.81)
Infection composite	138 (29.2%)	1.73 (1.42-2.11)	1.54 (1.25-1.88)
Serious infection proxy	96 (20.3%)	1.70 (1.36-2.13)	1.65 (1.31-2.08)
Pneumonia/lower respiratory infection	59 (12.5%)	2.17 (1.65-2.85)	1.74 (1.32-2.31)
COVID-19	60 (12.7%)	2.55 (1.94-3.35)	1.66 (1.25-2.21)
CMV	9 (1.9%)	1.20 (0.63-2.30)	1.29 (0.66-2.53)
Narrow opportunistic infection	20 (4.2%)	0.88 (0.57-1.38)	0.93 (0.59-1.46)
Pneumocystis jirovecii pneumonia (PJP)	2 (0.4%)	1.05 (0.30-3.65)	0.62 (0.15-2.52)
Progressive multifocal leukoencephalopathy (PML)	0 (0.0%)	0.22 (0.01-3.58)	Not estimable

Events are shown as n (%). The primary adjusted model included age group, sex, reporting region, B-cell lymphoma disease subgroup, and report year. Crude RORs used a Haldane–Anscombe 0.5 continuity correction.

ICANS was reported in 119 epcoritamab (8.1%), 47 glofitamab (5.0%), and 4 mosunetuzumab (0.8%) reports. Primary reporting odds were higher for epcoritamab (aROR 2.28, 95% CI 1.87 to 2.80). The glofitamab estimate was 1.26 (0.93 to 1.70), whereas extended treatment-context models produced larger estimates; it was therefore classified as covariate-sensitive. The sparse mosunetuzumab estimate did not indicate higher reporting (0.48, 0.18 to 1.29). Broadly defined neurotoxicity estimates were 1.70 (1.45 to 2.01), 0.90 (0.70 to 1.15), and 0.49 (0.30 to 0.81), respectively. These estimates describe reporting relative to the comparator and are not head-to-head neurotoxicity rates.

### Infection-associated reporting patterns

The infection composite was reported in 374 epcoritamab (25.6%), 156 glofitamab (16.5%), and 138 mosunetuzumab (29.2%) reports. Primary adjusted reporting odds were higher for epcoritamab (aROR 1.35, 95% CI 1.20 to 1.53) and mosunetuzumab (1.54, 1.25 to 1.88), whereas glofitamab had lower reporting odds (0.78, 0.65 to 0.93). Serious-infection proxy estimates were 1.52 (1.33 to 1.74), 0.81 (0.66 to 0.99), and 1.65 (1.31 to 2.08), respectively. Lower reporting odds do not establish lower infection incidence or lower clinical risk.

The composition of infection reporting differed by drug. Epcoritamab had higher reporting odds for CMV infection (aROR 4.54, 95% CI 3.66 to 5.62) and narrow opportunistic infection (2.20, 1.84 to 2.63). PJP and PML were uncommon, with 10 and 7 epcoritamab reports, respectively, and imprecise estimates. Mosunetuzumab had higher reporting odds for pneumonia/lower respiratory infection (1.74, 1.32 to 2.31) and COVID-19 (1.66, 1.25 to 2.21). Glofitamab did not show higher primary adjusted reporting odds for the selected specific infection endpoints.

### Report-level co-reporting of immune-effector and infectious phenotypes

Exact report-level intersections across CRS, ICANS, infection, opportunistic infection, and HLH are shown in **Figure 3A**. Among the seven mutually exclusive core phenotypes, 553 reports contained CRS only, 52 ICANS only, 465 infection only, 78 CRS plus ICANS, 163 CRS plus infection, 12 ICANS plus infection, and 28 CRS plus ICANS plus infection (**Figure 3B**, **Supplementary Table S7B**). An additional 202 reports met the narrow opportunistic-infection phenotype and 6 contained HLH plus infection; these categories were non-mutually exclusive. Drug-specific prevalences of these phenotypes are provided in **Supplementary Table S7C**.

**Figure 3:**
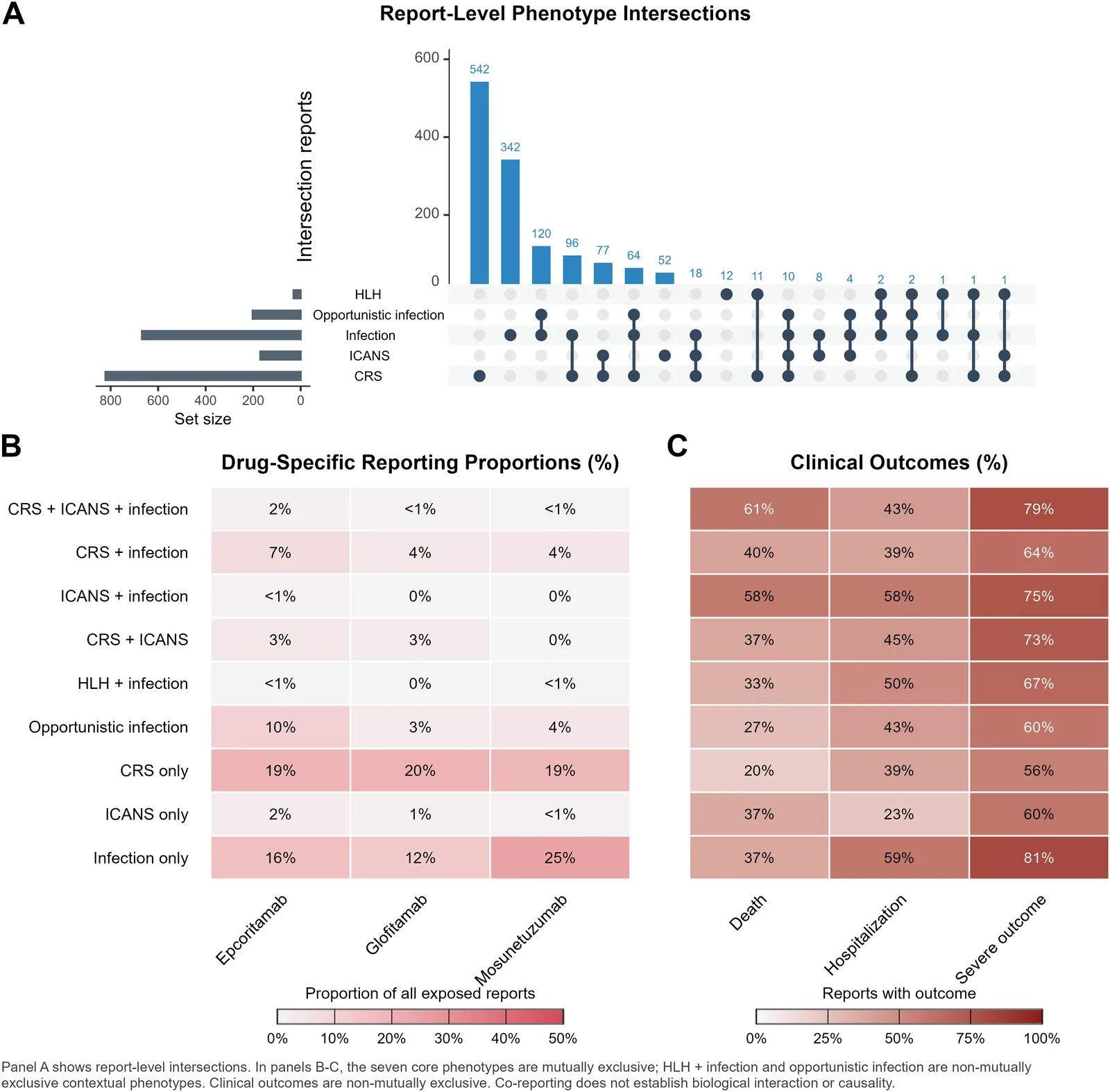
Report-level immune-effector and infection co-reporting patterns and clinical consequences. **(A)** UpSet analysis of report-level intersections among CRS, ICANS, infection composite, narrow opportunistic infection, and HLH in exposed FAERS reports. **(B)** Prevalence of prespecified phenotypes among all exposed reports for each drug. The seven CRS/ICANS/infection core phenotypes are mutually exclusive; HLH plus infection and narrow opportunistic infection are non-mutually exclusive contextual phenotypes. **(C)** Proportions of death, hospitalization, and severe outcomes among phenotype-positive reports. Outcomes are non-mutually exclusive.

Reports with overlapping phenotypes frequently contained serious outcomes (**Figure 3C**, **Supplementary Table S7D**). Among 28 triple-positive reports, death was recorded in 60.7%, hospitalization in 42.9%, and any severe outcome in 78.6%. Corresponding proportions for CRS plus infection were 39.9%, 39.3%, and 63.8%; for ICANS plus infection, they were 58.3%, 58.3%, and 75.0%. Infection-only reports contained death in 37.4%, hospitalization in 59.1%, and severe outcomes in 81.1%. These descriptive percentages may reflect differential coding completeness and case mix; they do not establish biological interaction, causal sequencing, or prognostic differences.

### Report-level timing of immune-effector and infection-associated endpoints

Complete dates sufficient for a primary TTO calculation were available for 613 of 1,463 epcoritamab reports (41.9%), 318 of 944 glofitamab reports (33.7%), and 289 of 472 mosunetuzumab reports (61.2%) (**Figures 4A, B**, **Supplementary Figure S3C**, and **Supplementary Table S7A**). Among reports with calculable dates, median CRS TTO was 8 days (IQR: 2–15) for epcoritamab and 1 day for glofitamab (0–2) and mosunetuzumab (1-10). ICANS medians were 12 days (5–15) and 1 day (0–2) for epcoritamab and glofitamab; only two mosunetuzumab ICANS reports had valid TTO. These drug-specific summaries were not formally compared and may reflect different step-up schedules, administration routes, and use of treatment-start rather than dose-level dates.

**Figure 4:**
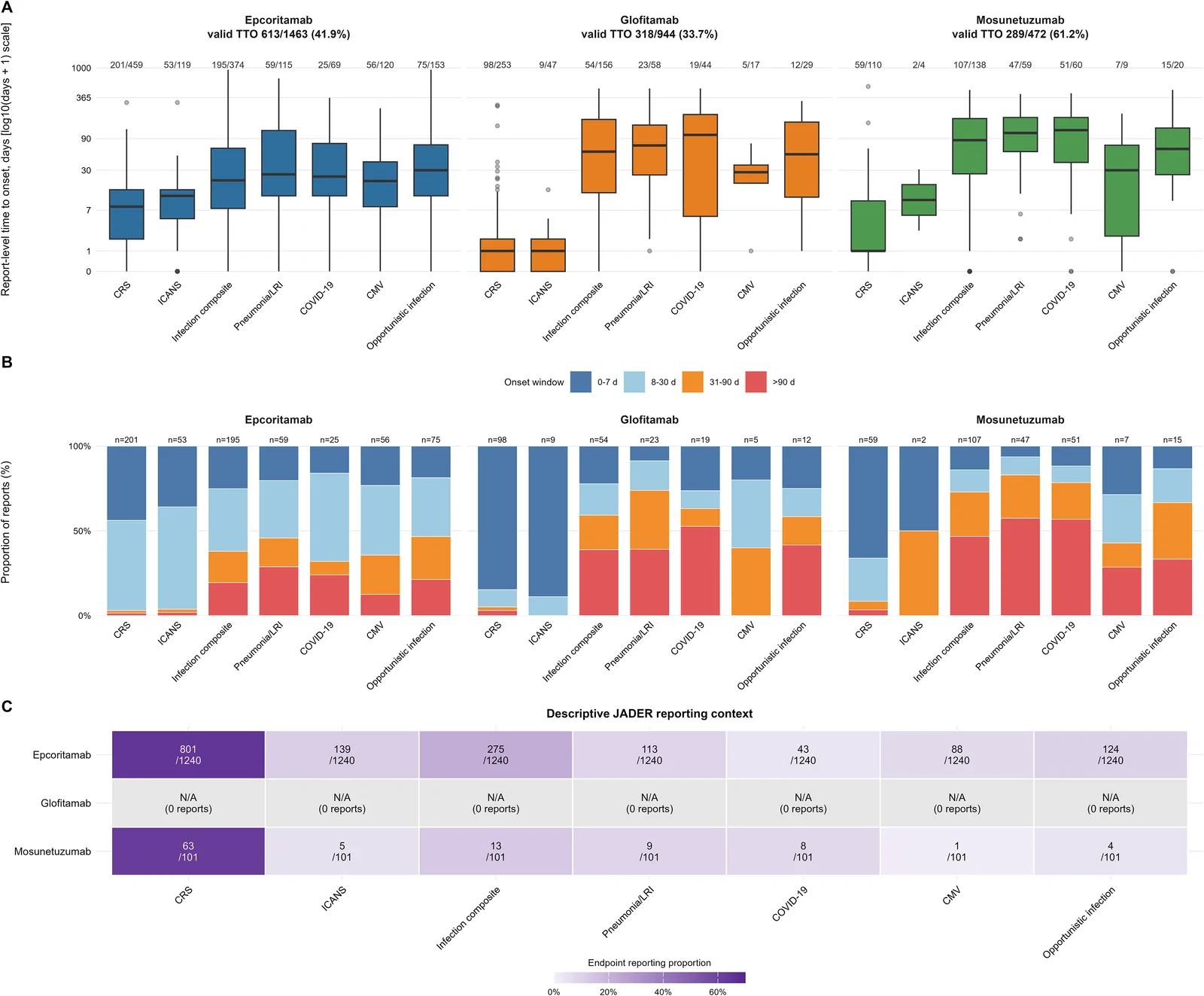
Time-to-onset profiles and descriptive JADER contextualization. **(A)** Report-level TTO distributions for seven prespecified endpoints by drug. Facet headings show reports with calculable TTO over all exposed reports: epcoritamab 613 of 1,463 (41.9%), glofitamab 318 of 944 (33.7%), and mosunetuzumab 289 of 472 (61.2%); endpoint-specific valid n/N values are printed above each distribution. TTO was calculated as DEMO.event_dt minus target-drug THER.start_dt, using complete eight-digit dates and retaining values from 0 to 3,650 days. The vertical scale is log_10_(days + 1). TTO was report-level, not dose-specific or adjudicated adverse-event-level onset, and no formal between-drug comparison was performed. **(B)** Onset windows categorized as 0–7, 8–30, 31–90, and >90 days among reports with calculable TTO. **(C)** Descriptive JADER reporting-context matrix for epcoritamab and mosunetuzumab. Cell labels show endpoint-positive reports over suspected-drug reports, and tile color maps the corresponding endpoint reporting proportion on a continuous percentage scale. Glofitamab was not evaluable because no suspected-drug reports were detected; mosunetuzumab had 101 reports.

Infection endpoints had broader and generally later report-level distributions. Median infection-composite TTO was 21 days (IQR: 7.5–64.5) for epcoritamab, 57.5 days (13.5–173.2) for glofitamab, and 85 days (26.5–178.5) for mosunetuzumab; pneumonia/lower respiratory infection medians were 26, 71, and 109 days. Reports with calculable TTO differed materially from those without calculable TTO in missing demographics, hospitalization, severe outcomes, calendar period, and, for mosunetuzumab, disease composition (**Supplementary Table S13**). TTO therefore describes a selected subset and supports temporal awareness rather than definitive drug-specific toxicity profiles.

### Sensitivity analyses, PT-level signals, and descriptive JADER contextualization

Model-specification analyses are summarized in **Supplementary Table S10** and **Supplementary Figure S4A**, whereas disease-, geography-, and calendar-restriction analyses are summarized in **Supplementary Table S11** and **Supplementary Figure S4B**. CRS was generally preserved across model specifications and major restrictions. Epcoritamab ICANS, CMV, and narrow opportunistic-infection reporting remained directionally consistent across model specifications. Glofitamab ICANS crossed 1 in the primary model but was higher in extended treatment-context models, demonstrating covariate sensitivity. Mosunetuzumab infection-composite, serious-infection, pneumonia/lower respiratory infection, and COVID-19 estimates were preserved across model specifications; infection-specific estimates attenuated or became unstable in US-only and common-calendar analyses. Aggressive-lymphoma and non-FL restrictions generally retained the CRS pattern, but sparse ICANS and CMV strata were not estimable for some drug-restriction combinations.

Excluding all primary-suspect CD20×CD3 reports from the comparator generally retained the qualitative CRS pattern and the main epcoritamab patterns, although estimates changed in magnitude and selected glofitamab and mosunetuzumab infection endpoints were attenuated or unstable (**Supplementary Table S12**). **Supplementary Figure S4C** summarizes the number of PT-level signals identified using the ROR+PRR rule and the conservative four-algorithm rule. After removal of indication, disease-progression, and other non-adverse-event terms, 436 PT-level drug-event rows met the minimum count threshold; 14 met all four conservative signal criteria (**Supplementary Figure S5**, **Supplementary Table S6**).

The JADER descriptive cohort contained 1,240 epcoritamab and 101 mosunetuzumab suspected-drug reports; no glofitamab reports were detected with the prespecified dictionary (**Figure 1C**). CRS was recorded in 801 epcoritamab (64.6%) and 63 mosunetuzumab (62.4%) reports. ICANS was recorded in 139 (11.2%) and 5 (5.0%), while the infection composite occurred in 275 (22.2%) and 13 (12.9%), respectively (**Figure 4C**, **Supplementary Figure S6**, **Supplementary Table S8**). The presence of broad CRS, ICANS, and infection categories in an independent reporting environment was descriptively concordant, but drug-specific proportions differed and were not statistically compared. Absence of glofitamab and the small mosunetuzumab denominator may reflect market uptake, reporting lag, or dictionary coverage and precluded validation of FAERS estimates.

**Table 3** contextualizes the drug-specific reporting patterns for clinical interpretation, with an extended label-informed matrix in **Supplementary Table S9**. CRS was shared, whereas non-CRS findings comprised epcoritamab ICANS, CMV, and opportunistic-infection reporting; covariate-sensitive glofitamab ICANS; and mosunetuzumab respiratory or viral infection reporting with geographic and calendar sensitivity. These patterns are intended to support phenotype-aware clinical awareness rather than comparative drug ranking.

**Table 3:** Drug-specific clinical contextualization of immune-effector and infection-associated reporting patterns.

Drug	Clinical context relevant to interpretation	Main postmarketing reporting pattern	Sensitivity-qualified interpretation for monitoring
Epcoritamab	Step-up dosing; recognized CRS, ICANS/neurologic toxicity, and serious infection concerns in treated B-cell lymphoma.	Shared CRS pattern; higher ICANS, CMV, and narrow opportunistic-infection reporting in the primary analysis.	Supports early CRS/ICANS vigilance and sustained viral/opportunistic-infection awareness beyond step-up dosing; ICANS, CMV, and opportunistic-infection directions were consistent across model specifications.
Glofitamab	Step-up dosing and anti-CD20 pretreatment/regimen context may influence reporting and adjustment.	Shared CRS pattern; ICANS CI crossed 1 in the primary model but was higher in extended treatment-context models; infection-composite reporting odds were lower in the primary model.	Supports early immune-effector toxicity vigilance. ICANS is a covariate-sensitive reporting pattern, not a comparative neurotoxicity ranking; lower infection-composite reporting odds do not imply lower infection incidence.
Mosunetuzumab	FL-heavy report composition and reporting-region/calendar differences are important interpretive constraints.	Shared CRS pattern; higher pneumonia/LRI and COVID-19 reporting in the primary analysis; ICANS sparse.	Supports early CRS awareness and continued respiratory/viral infection assessment, but infection-specific findings require caution because selected estimates attenuated or became unstable in US-only and common-calendar analyses.

Interpretations summarize postmarketing reporting patterns.

## Discussion

The principal contribution of this label-anchored analysis is not the re-identification of CRS as an expected class toxicity, but the characterization of report-level immune-effector and infection co-reporting, drug-specific reporting patterns, and TTO distributions relevant to clinical assessment of CD20×CD3 bispecific-antibody toxicity in B-cell lymphoma. CRS was shared and generally preserved across model specifications and major restrictions. Non-CRS findings were more conditional: epcoritamab showed higher ICANS, CMV, and opportunistic-infection reporting; glofitamab ICANS was covariate-sensitive; and mosunetuzumab respiratory and viral infection estimates were sensitive to reporting geography and calendar period. These distinctions are reporting patterns, not comparative safety rankings.

The report-level timing pattern is compatible with, but does not prove, an early immune-effector phase followed by continued infection-associated reporting. Early CRS and ICANS reporting is clinically consistent with CD3-mediated activation during step-up treatment, whereas broader infection distributions may arise from B-cell depletion, cytopenias, prior therapy, immunosuppressive management, lymphoma-related immune dysfunction, and longer opportunity for follow-up (12–16). Because FAERS does not provide complete longitudinal treatment or microbiologic data, neither temporal ordering nor mechanism can be inferred from these observations.

The shared CRS reporting pattern was clinically coherent with established CD3-engager toxicity (1, 2, 12, 13). Its consistency across disease, geography, calendar, model-specification, and comparator-exclusion analyses made it the most stable finding. Median report-level TTO was 1 day for glofitamab and mosunetuzumab CRS and 8 days for epcoritamab. Different step-up schedules, administration routes, documentation practices, and use of treatment-start rather than dose-level dates limit interpretation of this contrast. No formal between-drug test was performed, and the result does not indicate intrinsically earlier toxicity for one agent (9–12).

ICANS reporting differed more markedly. Epcoritamab had higher reporting odds in the primary model. For glofitamab, the primary CI crossed 1 while extended models containing treatment-context indicators yielded larger estimates. This discrepancy suggests sensitivity to disease, reporting, pretreatment, regimen, or post-event management variables and precludes a simple drug-specific conclusion. Mosunetuzumab had only four ICANS reports. Broadly defined neurotoxicity captured nonspecific neurologic coding but similarly cannot substitute for adjudicated ASTCT-grade ICANS (13, 17). These observations support attention to neurologic change without establishing comparative neurotoxicity.

Infection-associated results require similarly qualified interpretation. Epcoritamab CMV and narrow opportunistic-infection reporting remained directionally consistent across model specifications. Mosunetuzumab pneumonia/lower respiratory infection and COVID-19 had higher reporting odds in the primary model, but selected estimates attenuated or became unstable in US-only and common-calendar analyses. The FL-heavy mosunetuzumab composition did not fully explain the pattern in non-FL analyses, yet differences in disease indication, market maturity, prior therapy, prophylaxis, and follow-up remain plausible sources of channeling and reporting bias. The established follicular-lymphoma treatment context of mosunetuzumab heightens this concern (34). Clinical and observational evidence supports infection vigilance after bispecific therapy (12–16, 35, 36), but these data do not estimate comparative infection risk.

The co-reporting analysis addresses a clinically important diagnostic overlap. Fever, hypotension, hypoxia, encephalopathy, cytopenias, and inflammatory-marker elevation can cure recorded with CRS, infection, ICANS, or HLH-like syndromes. Reports containing multiple domains frequently included serious outcomes, but more severe reports may also receive more complete adverse-event coding. Thus, co-reporting does not establish biological interaction, temporal order, or causal sequencing. It supports considering infection in parallel when evaluating suspected immune-effector toxicity, guided by the individual presentation rather than by a fixed algorithm derived from these data.

The TTO findings provide context rather than comparative timing evidence. Reports containing infection endpoints had broader and later distributions than CRS reports, supporting continued clinical awareness beyond step-up dosing. However, only 33.7%–61.2% of drug reports had calculable TTO, and the valid subset differed from the remainder in demographics, hospitalization, serious outcomes, calendar period, and disease composition. TTO also used treatment-start rather than dose-level dates. The observed distributions should therefore guide awareness, not define optimal surveillance duration or deterministic cutoffs. Prophylaxis, immunoglobulin monitoring, and infection evaluation should remain individualized according to existing guidance and patient context (12–14).

JADER supplied an independent reporting environment but only descriptive contextualization. Broad CRS, ICANS, and infection categories were present for epcoritamab and mosunetuzumab, yet proportions differed from FAERS and were not formally tested. Glofitamab was not detected, and only 101 mosunetuzumab reports were available. Market availability, uptake, reporting lag, dictionary coverage, and database structure may explain both concordant and discordant observations. JADER therefore neither replicated the adjusted FAERS models nor supported confirmatory inference.

Study strengths include current label-informed endpoint definitions, a B-cell lymphoma comparator, removal of indication and progression terms from PT displays, explicit report-level co-reporting, TTO completeness analysis, and sensitivity analyses addressing model specification, disease mix, geography, calendar time, and comparator construction. The primary model deliberately prioritized demographic, disease, geographic, and calendar variables; treatment-context indicators were moved to extended sensitivity models because they may be downstream of toxicity or reflect regimen context. **Table 3** provides a drug-level clinical interpretation framework, whereas **Supplementary Table S1** defines endpoint construction and analysis tiers.

Sensitivity analyses qualified the consistency of the non-CRS findings. Glofitamab ICANS was covariate-sensitive. Mosunetuzumab infection-specific estimates were less stable in US-only and common-calendar analyses, and several ICANS or CMV strata were too sparse for interpretation. Comparator exclusion generally preserved the shared CRS pattern but changed estimate magnitudes and attenuated selected infection endpoints. These results reinforce that the analysis identifies reporting patterns, not comparative drug-safety rankings.

Several limitations are fundamental. FAERS and JADER are subject to under-reporting, selective and incomplete follow-up, missing denominators, stimulated reporting, and notoriety bias (19, 20). Label-anchored endpoints improve clinical coherence but may preferentially recapture well-publicized toxicities. Market maturity and exposure differed across agents, and line of therapy, dose-level step-up schedule, prophylaxis, immunoglobulin replacement, microbiology, and cumulative prior treatment were unavailable or incomplete. Indication and treatment channeling remain possible despite lymphoma restriction; the FL-heavy mosunetuzumab reports illustrate this concern. RORs cannot estimate incidence, attributable effects, causality, or comparative treatment risk.

Treatment-context indicators may represent prior therapy, concomitant exposure, regimen design, or post-event management; adjusting for corticosteroid or anti-CD20 therapy may therefore introduce overadjustment. Disease composition, geography, and calendar period influenced selected non-CRS estimates, and some restricted strata were sparse. MedDRA phenotypes were not adjudicated diagnoses, broad composites traded specificity for sensitivity, outcome fields were selectively completed, and co-reporting may reflect reporting density. TTO was report-level, substantially missing, selected, and not dose-specific or adverse-event-level onset. JADER was descriptive contextualization, not validation. Prospective registries should capture dose-level timing, ASTCT grading, microbiologic adjudication, prophylaxis, immunoglobulin and lymphocyte recovery, prior therapies, and longitudinal outcomes.

## Conclusions

CD20×CD3 bispecific antibodies shared a generally stable CRS reporting pattern in B-cell lymphoma, whereas neurotoxicity and infection-associated findings were more drug- and analysis-dependent. Report-level co-reporting supports considering inflammatory toxicity and infection in parallel when presentations overlap, and broader infection-associated TTO distributions support continued awareness beyond step-up dosing. Clinical monitoring and prophylaxis should nevertheless follow established guidance and individual patient context. These spontaneous-reporting findings are hypothesis-generating and should not be interpreted as incidence estimates, causal effects, biological interaction, or comparative drug-safety rankings.

## Data Availability

The raw data supporting the conclusions of this article will be made available by the authors, without undue reservation.
