# Supplementary material for: Immune-effector and infection-associated reporting patterns of CD20×CD3 bispecific antibodies in B-cell lymphoma: a label-anchored FAERS analysis with descriptive JADER contextualization
Source: Front Immunol. 2026 Oct 2;17:1920122. doi: 10.3389/fimmu.2026.1920122 (PMC13672536; doi:10.3389/fimmu.2026.1920122)
Supplement: Supplementary file 1 [file DataSheet1.pdf]

Supplementary Figure Legends

Supplementary Figure S1. FDA label-anchored framework for immune-effector and infection-associated toxicity phenotyping. (A) Label-derived safety anchors for epcoritamab, glofitamab, and mosunetuzumab. (B) Prespecified clinical endpoint families covering immune-effector, infectious, and hematologic toxicities. (C) Label-informed working hypotheses used to guide drug-specific phenotyping.

Supplementary Figure S2. Detailed FAERS analysis and descriptive JADER contextualization workflow. The FAERS pathway provides the detailed workflow underlying Figure 1A, including deduplication, target-drug ascertainment, primary-suspect restriction, and construction of the final exposed cohort. Comparator and extraction-audit insets are descriptive checks rather than filtering steps. The JADER pathway provides descriptive contextualization.

Supplementary Figure S3. Reporting trends and date/time-to-onset availability. (A) Quarterly report counts by drug based on the initial FDA receipt date. (B) Quarterly counts of CRS, ICANS, and infection reports by drug using a shared y-axis. (C) Proportions of reports with complete event dates, target-drug start dates, and calculable time-to-onset (TTO) values.

Supplementary Figure S4. Sensitivity analyses of disproportionality signals. (A) Primary and extended model-specification estimates; cell colors represent log2(aROR), and text shows the corresponding aROR. (B) Disease-, geography-, and calendar-restricted analyses classified relative to the full-cohort primary model as preserved, attenuated, or unstable. (C) PT-level positive signal counts under the ROR+PRR rule and the conservative four-algorithm rule.

Supplementary Figure S5. PT-level pharmacovigilance signal landscape. Drug-specific PT-level landscape among terms with at least three exposed reports after exclusion of indication, disease-progression, and other prespecified non-adverse-event terms. The x-axis shows log2(ROR), the y-axis shows exposed-event report counts on a logarithmic scale, point size reflects report count, and colors denote prespecified clinical categories. Labels identify the highest-scoring PTs according to the prespecified visualization rule.

Supplementary Figure S6. JADER reaction-term details. Top cleaned JADER reaction terms for epcoritamab and mosunetuzumab, ranked by unique latest suspected-drug reports. Lymphoma indications and disease-progression terms were excluded. Glofitamab was not detected in JADER.

Abbreviations: aROR, adjusted reporting odds ratio; CRS, cytokine release syndrome; FAERS, FDA Adverse Event Reporting System; ICANS, immune effector cell-associated neurotoxicity syndrome; JADER, Japanese Adverse Drug Event Report database; PRR, proportional reporting ratio; PT, preferred term; ROR, reporting odds ratio; TTO, time to onset.

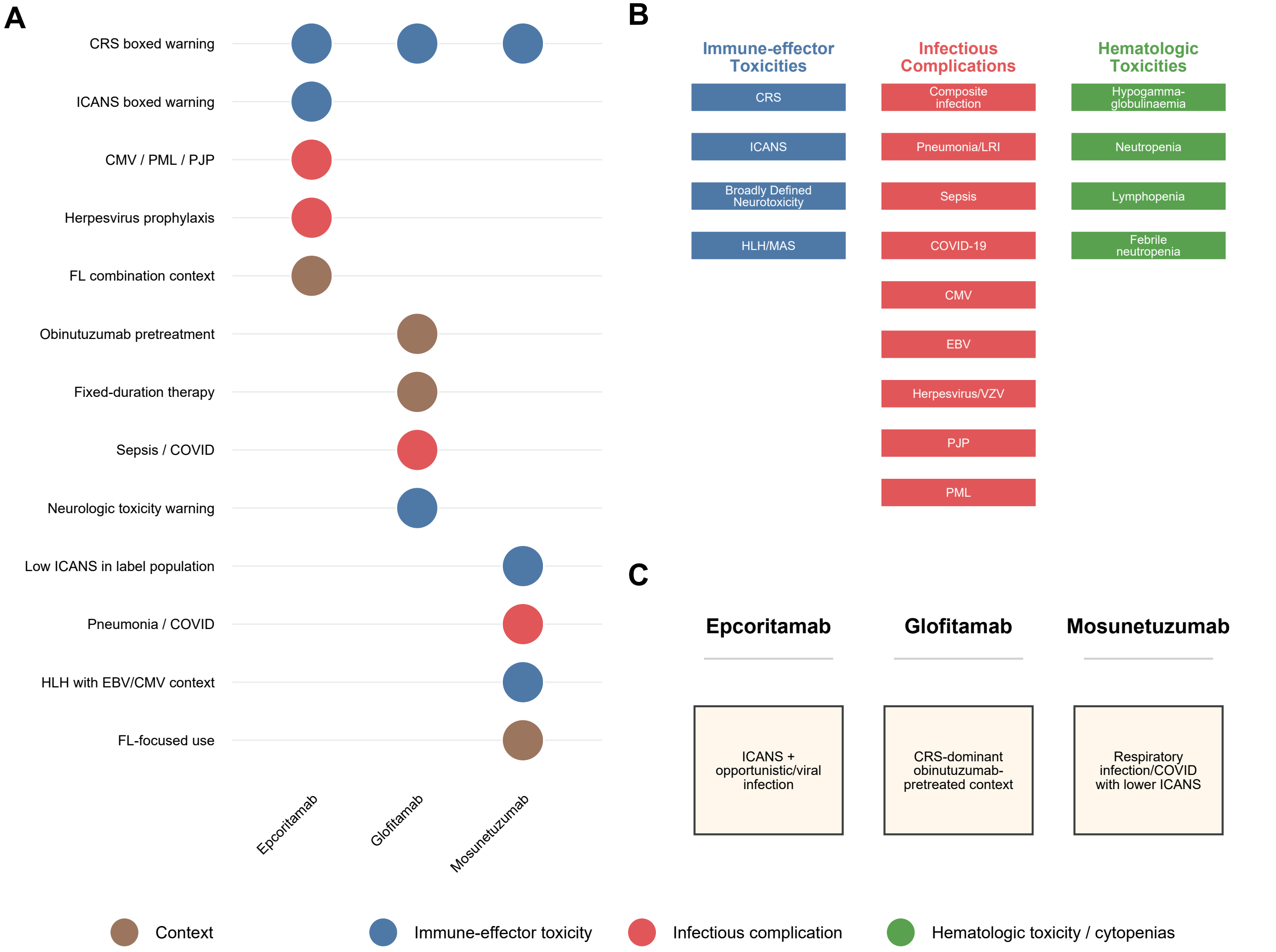

A
CRS boxed warning
ICANS boxed warning
CMV / PML / PJP
Herpesvirus prophylaxis
FL combination context
Obinutuzumab pretreatment
Fixed-duration therapy
Sepsis / COVID
Neurologic toxicity warning
Low ICANS in label population
Pneumonia / COVID
HLH with EBV/CMV context
FL-focused use
Epcoritamab
Glofitamab
Mosunetuzumab
B
Immune-effector Toxicities
CRS
ICANS
Broadly Defined Neurotoxicity
HLH/MAS
Infectious Complications
Composite infection
Pneumonia/LRI
Sepsis
COVID-19
CMV
EBV
Herpesvirus/VZV
PJP
PML
Hematologic Toxicities
Hypogamma-globulinaemia
Neutropenia
Lymphopenia
Febrile neutropenia
C
Epcoritamab
ICANS + opportunistic/viral infection
Glofitamab
CRS-dominant obinutuzumab-pretreated context
Mosunetuzumab
Respiratory infection/COVID with lower ICANS
Context
Immune-effector toxicity
Infectious complication
Hematologic toxicity / cytopenias

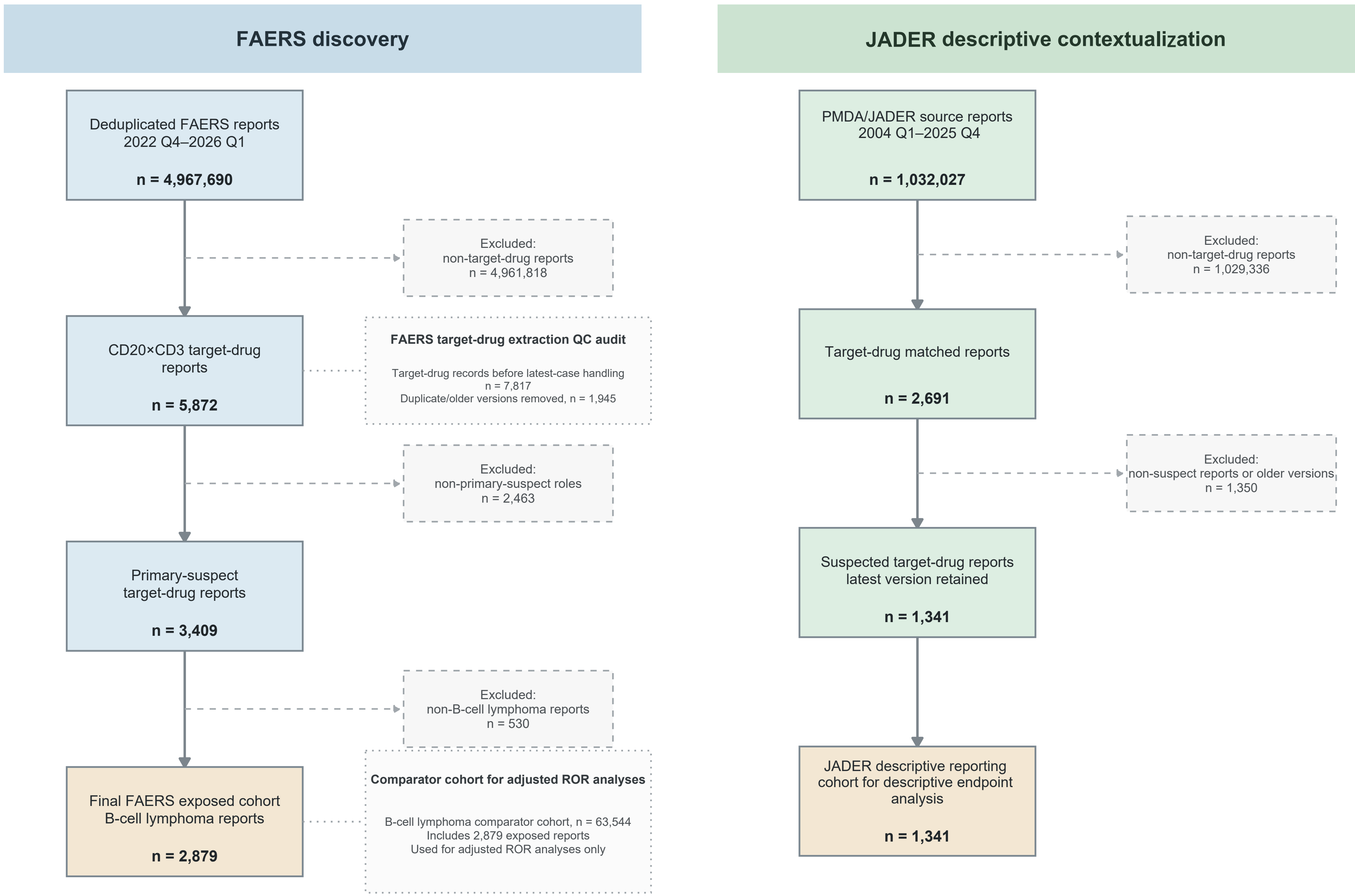

Endpoint analyses used the final FAERS exposed cohort; time-to-onset analyses were restricted to reports with calculable onset dates.

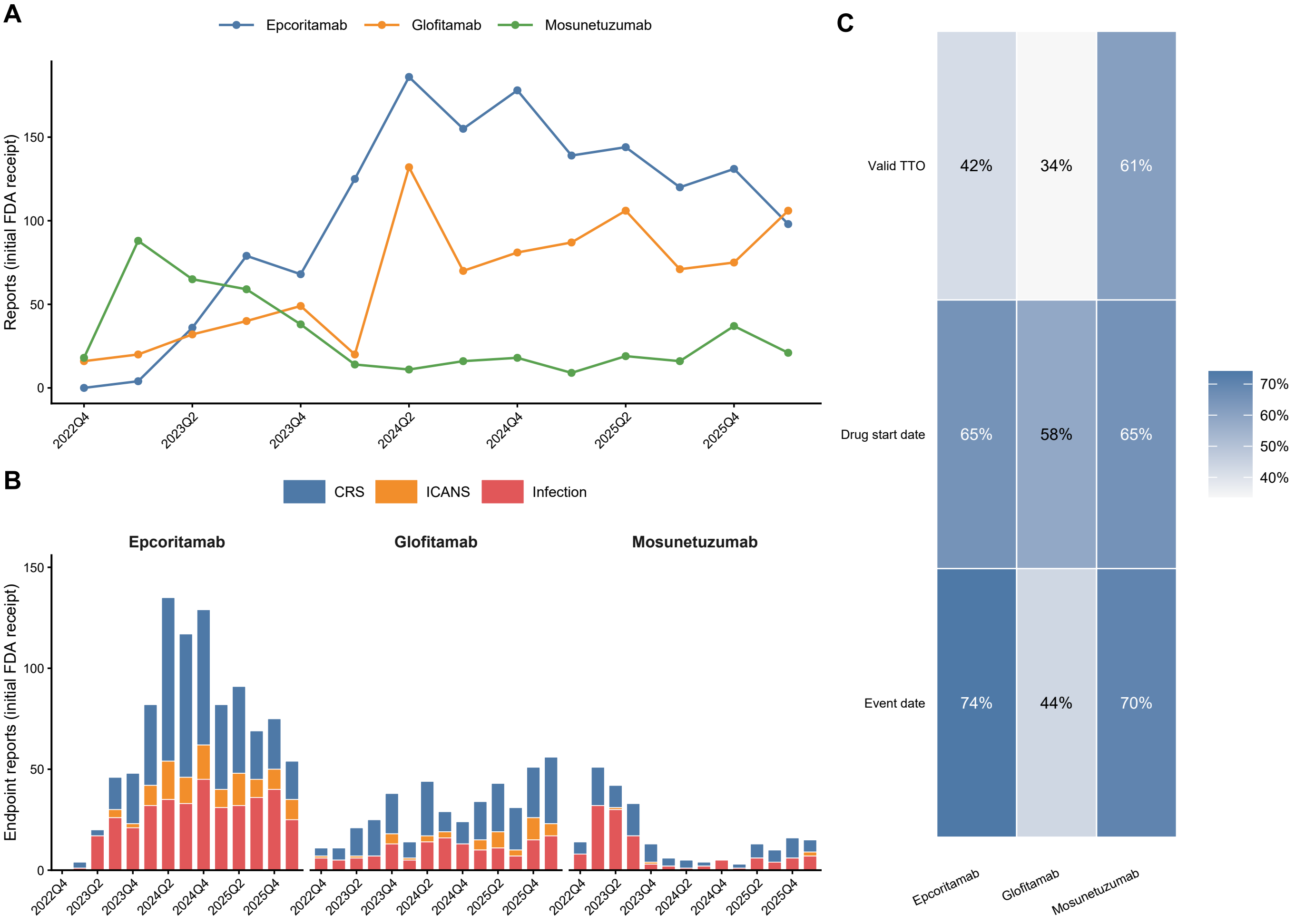
A
Epcoritamab
Glofitamab
Mosunetuzumab
Reports (initial FDA receipt)
0
50
100
150
2022Q4
2023Q2
2023Q4
2024Q2
2024Q4
2025Q2
2025Q4
B
CRS
ICANS
Infection
Epcoritamab
Glofitamab
Mosunetuzumab
Endpoint reports (initial FDA receipt)
C
Valid TTO
Drug start date
Event date
42%
34%
61%
65%
58%
65%
74%
44%
70%
70%
60%
50%
40%

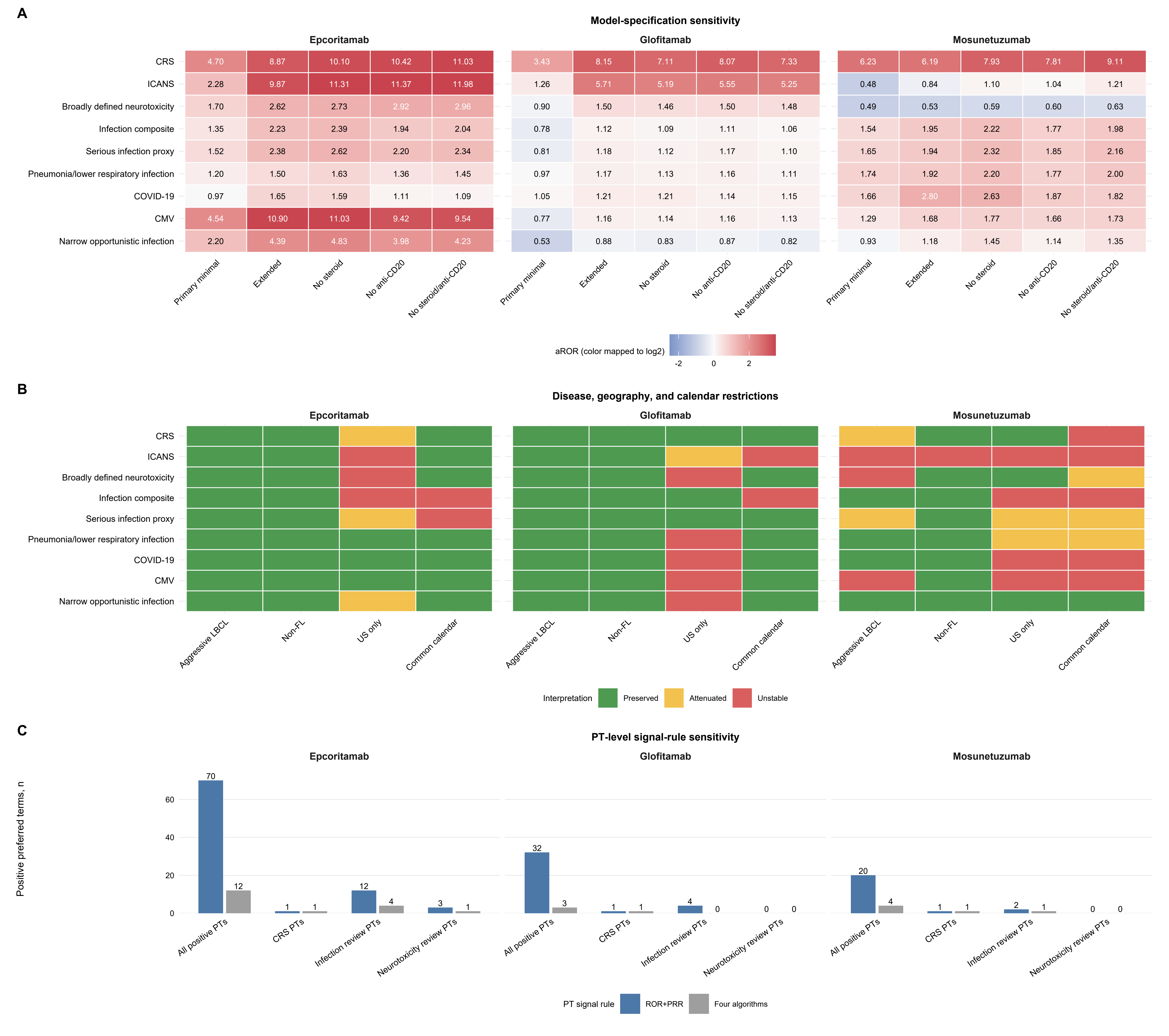
A
Model-specification sensitivity
Epcoritamab
Glofitamab
Mosunetuzumab
CRS 4.70 8.87 10.10 10.42 11.03 3.43 8.15 7.11 8.07 7.33 6.23 6.19 7.93 7.81 9.11
ICANS 2.28 9.87 11.31 11.37 11.98 1.26 5.71 5.19 5.55 5.25 0.48 0.84 1.10 1.04 1.21
Broadly defined neurotoxicity 1.70 2.62 2.73 2.92 2.96 0.90 1.50 1.46 1.50 1.48 0.49 0.53 0.59 0.60 0.63
Infection composite 1.35 2.23 2.39 1.94 2.04 0.78 1.12 1.09 1.11 1.06 1.54 1.95 2.22 1.77 1.98
Serious infection proxy 1.52 2.38 2.62 2.20 2.34 0.81 1.18 1.12 1.17 1.10 1.65 1.94 2.32 1.85 2.16
Pneumonia/lower respiratory infection 1.20 1.50 1.63 1.36 1.45 0.97 1.17 1.13 1.16 1.11 1.74 1.92 2.20 1.77 2.00
COVID-19 0.97 1.65 1.59 1.11 1.09 1.05 1.21 1.21 1.14 1.15 1.66 2.80 2.63 1.87 1.82
CMV 4.54 10.90 11.03 9.42 9.54 0.77 1.16 1.14 1.16 1.13 1.29 1.68 1.77 1.66 1.73
Narrow opportunistic infection 2.20 4.39 4.83 3.98 4.23 0.53 0.88 0.83 0.87 0.82 0.93 1.18 1.45 1.14 1.35
Primary minimal
Extended
No steroid
No anti-CD20
No steroid/anti-CD20
aROR (color mapped to log2)
-2
0
2
B
Disease, geography, and calendar restrictions
Epcoritamab
Glofitamab
Mosunetuzumab
CRS
ICANS
Broadly defined neurotoxicity
Infection composite
Serious infection proxy
Pneumonia/lower respiratory infection
COVID-19
CMV
Narrow opportunistic infection
Aggressive LBCL
Non-FL
US only
Common calendar
Interpretation
Preserved
Attenuated
Unstable
C
PT-level signal-rule sensitivity
Epcoritamab
Glofitamab
Mosunetuzumab
Positive preferred terms, n
0
20
40
60
70 12 1 1 12 4 3 1
32 3 1 1 4 0 0 0
20 4 1 1 2 1 0 0
All positive PTs
CRS PTs
Infection review PTs
Neurotoxicity review PTs
PT signal rule
ROR+PRR
Four algorithms

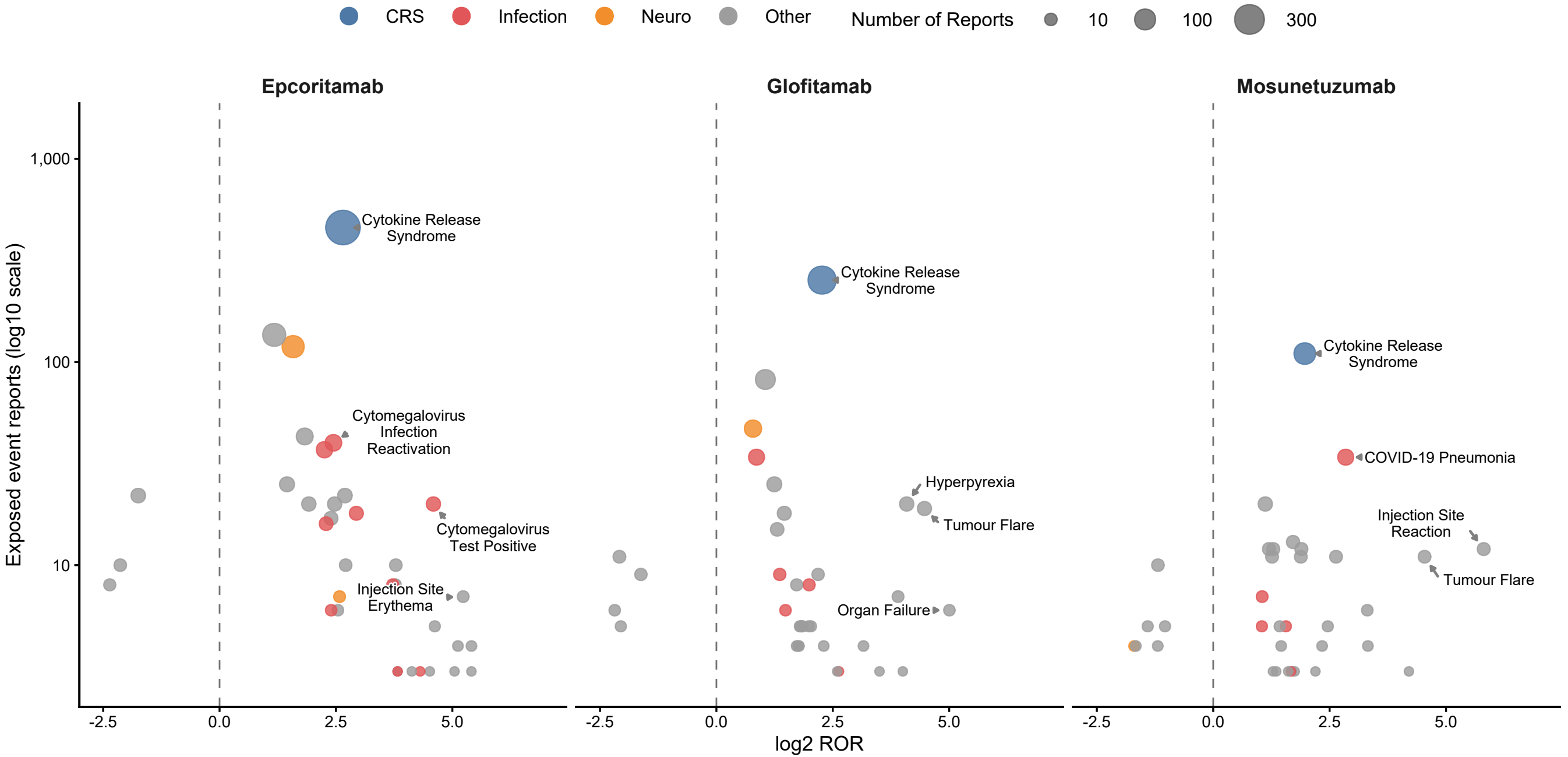

Note: Labels denote the four highest-scoring PTs per drug, ranked by |log2 ROR| x log10(exposed event reports + 1), after exclusion of non-AE indication/progression/administrative/outcome terms.

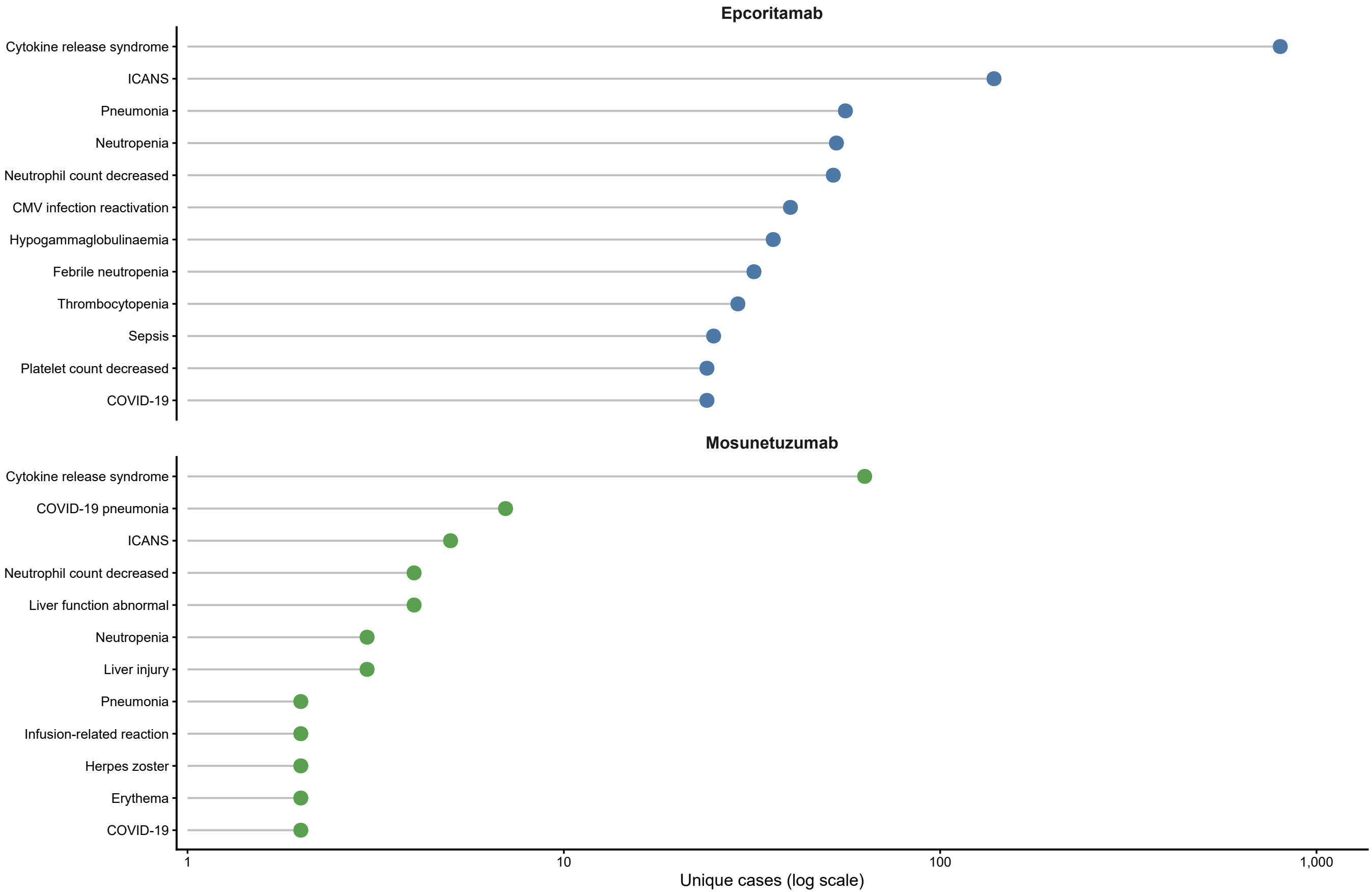
Epcoritamab
Cytokine release syndrome
ICANS
Pneumonia
Neutropenia
Neutrophil count decreased
CMV infection reactivation
Hypogammaglobulinaemia
Febrile neutropenia
Thrombocytopenia
Sepsis
Platelet count decreased
COVID-19
Mosunetuzumab
Cytokine release syndrome
COVID-19 pneumonia
ICANS
Neutrophil count decreased
Liver function abnormal
Neutropenia
Liver injury
Pneumonia
Infusion-related reaction
Herpes zoster
Erythema
COVID-19
1
10
100
1,000
Unique cases (log scale)

Supplementary Table S1. Endpoint categories and analysis tiers.

Endpoint Category	Rationale (FDA Label Anchor)	Evaluated MedDRA Endpoints	Analysis Tier
Immune-Effector Toxicity	CRS and ICANS boxed warnings	CRS, ICANS, Broadly Defined Neurotoxicity, HLH/MAS	Primary / Key Secondary
Infection & Opportunistic Events	Broad serious and opportunistic infection warnings	Composite infection, pneumonia/LRI, sepsis, COVID-19, CMV, HSV/VZV, PJP, PML	Primary / Key Secondary
Immune-Suppression Markers	Expected mechanism of action / Cytopenia warnings	Hypogammaglobulinaemia, neutropenia, lymphopenia, febrile neutropenia	Secondary

Notes: Endpoint categories were defined based on FDA label-anchored safety domains and mapped to evaluated MedDRA endpoints. Analysis tiers indicate whether endpoints were used for primary, key secondary, or secondary analyses.

Abbreviations: CRS, cytokine release syndrome; ICANS, immune effector cell-associated neurotoxicity syndrome; HLH/MAS, haemophagocytic lymphohistiocytosis/macrophage activation syndrome; LRI, lower respiratory infection; CMV, cytomegalovirus; HSV/VZV, herpes simplex virus/varicella-zoster virus; PJP, Pneumocystis jirovecii pneumonia; PML, progressive multifocal leukoencephalopathy; MedDRA, Medical Dictionary for Regulatory Activities; TTO, time to onset; ROR, reporting odds ratio.

Supplementary Table S2A. Drug search strategy used for JADER database queries

Generic Name	Brand Name(s)	JADER Database Search Strategy
Epcoritamab	EPKINLY	EPCORITAMAB; EPKINLY; epcoritamab (including Japanese brand/generic equivalents)
Glofitamab	COLUMVI	GLOFITAMAB; COLUMVI; glofitamab (including Japanese brand/generic equivalents)
Mosunetuzumab	LUNSUMIO	MOSUNETUZUMAB; LUNSUMIO; mosunetuzumab (including Japanese brand/generic equivalents)

Abbreviation: JADER, Japanese Adverse Drug Event Report.
Note: Search terms include generic names, brand names, and Japanese brand/generic equivalents where applicable.

Supplementary Table S2B. Disease cohort search concepts for lymphoma identification

Disease Cohort (Analysis Label)	Included Search Concepts (MedDRA / FAERS terms)
LBCL/DLBCL/high-grade B-cell lymphoma	DLBCL, large B-cell lymphoma, high-grade B-cell lymphoma, aggressive B-cell lymphoma
Follicular lymphoma	Follicular lymphoma, FL
Other or unspecified B-cell lymphoma/NHL	B-cell lymphoma, non-Hodgkin lymphoma, lymphoma unspecified

Abbreviations: CD20×CD3, CD20/CD3 bispecific antibody; DLBCL, diffuse large B-cell lymphoma; FAERS, FDA Adverse Event Reporting System; FL, follicular lymphoma; LBCL, large B-cell lymphoma; MedDRA, Medical Dictionary for Regulatory Activities; NHL, non-Hodgkin lymphoma.

Note: Search concepts are presented as provided in the source table and were used to define disease-cohort labels for the FAERS analysis.

Supplementary Table S3. Safety endpoint definitions and MedDRA search strategy

Safety Endpoint Domain	MedDRA Strategy Source	Included Preferred Terms (PTs) / Search Definition
**CRS**	Core PT	Cytokine release syndrome
**ICANS**	Core PT	Immune effector cell-associated neurotoxicity syndrome
**Broadly Defined Neurotoxicity**	Core / Curated PTs / SMQ	ICANS core PT plus neurotoxicity, encephalopathy, confusion, aphasia, seizure, and delirium curated PTs; along with noninfectious encephalopathy/delirium SMQ (narrow)
**Pneumonia**	SMQ narrow	Infective pneumonia (SMQ), narrow scope
**Sepsis**	SMQ narrow	Sepsis (SMQ), narrow scope
**COVID-19**	SMQ narrow (Cleaned/Curated)	COVID-19 (SMQ), narrow scope, excluding immunisation, prophylaxis, screening, treatment, and vaccine PTs
**Opportunistic Infection (Narrow)**	SMQ narrow	Opportunistic infections (SMQ), narrow scope
**Opportunistic Infection (Any)**	SMQ broad/narrow	Opportunistic infections (SMQ), narrow or broad scope
**CMV**	Curated PT Regex	Cytomegalovirus / CMV terms
**HSV/VZV**	Curated PT Regex	Herpes, zoster, and varicella terms
**Fungal Infection**	Curated PT Regex	Fungal, Candida, Aspergillus, Mucor, and Cryptococcus terms
**PJP**	Curated PT Regex	Pneumocystis / PJP terms
**PML**	Curated PT Regex	Progressive multifocal leukoencephalopathy / PML terms
**Infection Composite**	Composite Endpoint	Pneumonia, sepsis, COVID-19, opportunistic infection, CMV, HSV/VZV, fungal infection, PJP, or PML
**Serious Infection Proxy**	Composite Endpoint	Pneumonia, sepsis, opportunistic infection (narrow), CMV, fungal infection, PJP, or PML

Note: PT, Preferred Term; SMQ, Standardised MedDRA Query; CRS, cytokine release syndrome; ICANS, immune effector cell-associated neurotoxicity syndrome; CMV, cytomegalovirus; HSV, herpes simplex virus; VZV, varicella-zoster virus; PJP, Pneumocystis jirovecii pneumonia; PML, progressive multifocal leukoencephalopathy.

Supplementary Table S4. Reporting odds ratios of safety endpoints by comparator cohort.

Drug	Safety Endpoint	Drug Exposure (Events / Total)	Comparator Exposure (Events / Total)	ROR (95% CI)	Signal Detected
Full FAERS Database					
Epcoritamab	CRS	501 / 1713	11284 / 4965977	181.60 (163.40-201.80)	Yes
	ICANS	136 / 1713	3959 / 4965977	108.40 (90.79-129.50)	Yes
	Broadly Defined Neurotoxicity	195 / 1713	243845 / 4965977	2.49 (2.15-2.89)	Yes
	Infection Composite	474 / 1713	537797 / 4965977	3.15 (2.83-3.50)	Yes
	Serious Infection	337 / 1713	181305 / 4965977	6.47 (5.74-7.29)	Yes
	Pneumonia/LRI	136 / 1713	135539 / 4965977	3.08 (2.59-3.67)	Yes
	Sepsis	85 / 1713	44109 / 4965977	5.86 (4.71-7.28)	Yes
	COVID-19	75 / 1713	109721 / 4965977	2.04 (1.62-2.57)	Yes
	Viral/Opportunistic Infection	145 / 1713	36543 / 4965977	12.51 (10.56-14.83)	Yes
	Fungal Infection	24 / 1713	23815 / 4965977	3.01 (2.02-4.49)	Yes
	PJP	10 / 1713	3737 / 4965977	8.18 (4.46-15.02)	Yes
	HBV Reactivation	2 / 1713	941 / 4965977	7.70 (2.22-26.68)	No
	PML	7 / 1713	1844 / 4965977	11.83 (5.76-24.27)	Yes
	Hypogammaglobulinaemia	52 / 1713	4605 / 4965977	34.04 (25.82-44.87)	Yes
	Neutropenia	109 / 1713	66729 / 4965977	5.01 (4.13-6.08)	Yes
	Lymphopenia	13 / 1713	8817 / 4965977	4.46 (2.61-7.63)	Yes
	Febrile Neutropenia	26 / 1713	18156 / 4965977	4.28 (2.92-6.28)	Yes
Glofitamab	CRS	313 / 1157	11472 / 4966533	160.30 (140.70-182.80)	Yes
	ICANS	54 / 1157	4041 / 4966533	60.64 (46.12-79.74)	Yes
	Broadly Defined Neurotoxicity	84 / 1157	243956 / 4966533	1.52 (1.22-1.90)	Yes
	Infection Composite	254 / 1157	538017 / 4966533	2.32 (2.02-2.67)	Yes
	Serious Infection	139 / 1157	181503 / 4966533	3.61 (3.02-4.31)	Yes
	Pneumonia/LRI	78 / 1157	135597 / 4966533	2.59 (2.06-3.26)	Yes
	Sepsis	42 / 1157	44152 / 4966533	4.25 (3.13-5.77)	Yes
	COVID-19	67 / 1157	109729 / 4966533	2.74 (2.14-3.50)	Yes
	Viral/Opportunistic Infection	33 / 1157	36655 / 4966533	4.01 (2.84-5.65)	Yes
	Fungal Infection	22 / 1157	23817 / 4966533	4.11 (2.71-6.24)	Yes
	PJP	4 / 1157	3743 / 4966533	5.17 (2.05-13.06)	Yes
	HBV Reactivation	0 / 1157	943 / 4966533	2.27 (0.14-36.40)	No
	PML	4 / 1157	1847 / 4966533	10.48 (4.15-26.49)	Yes
	Hypogammaglobulinaemia	14 / 1157	4643 / 4966533	13.55 (8.07-22.76)	Yes
	Neutropenia	94 / 1157	66744 / 4966533	6.52 (5.29-8.05)	Yes
	Lymphopenia	6 / 1157	8824 / 4966533	3.17 (1.47-6.86)	Yes
	Febrile Neutropenia	20 / 1157	18162 / 4966533	4.91 (3.17-7.60)	Yes
Mosunetuzumab	CRS	122 / 539	11663 / 4967151	124.70 (101.80-152.60)	Yes
	ICANS	5 / 539	4090 / 4967151	12.48 (5.39-28.94)	Yes
	Broadly Defined Neurotoxicity	19 / 539	244021 / 4967151	0.72 (0.46-1.14)	No
	Infection Composite	162 / 539	538109 / 4967151	3.54 (2.95-4.26)	Yes

Drug	Safety Endpoint	Drug Exposure (Events / Total)	Comparator Exposure (Events / Total)	ROR (95% CI)	Signal Detected
	Serious Infection	100 / 539	181542 / 4967151	6.03 (4.85-7.49)	Yes
	Pneumonia/LRI	63 / 539	135612 / 4967151	4.75 (3.65-6.17)	Yes
	Sepsis	23 / 539	44171 / 4967151	5.07 (3.35-7.67)	Yes
	COVID-19	62 / 539	109734 / 4967151	5.79 (4.45-7.54)	Yes
	Viral/Opportunistic Infection	20 / 539	36668 / 4967151	5.31 (3.41-8.25)	Yes
	Fungal Infection	10 / 539	23829 / 4967151	4.11 (2.23-7.58)	Yes
	PJP	2 / 539	3745 / 4967151	6.16 (1.78-21.36)	No
	HBV Reactivation	1 / 539	942 / 4967151	14.68 (2.95-72.98)	No
	PML	0 / 539	1851 / 4967151	2.48 (0.15-39.80)	No
	Hypogammaglobulinaemia	9 / 539	4648 / 4967151	19.12 (10.06-36.34)	Yes
	Neutropenia	32 / 539	66806 / 4967151	4.70 (3.29-6.70)	Yes
	Lymphopenia	1 / 539	8829 / 4967151	1.56 (0.32-7.77)	No
	Febrile Neutropenia	10 / 539	18172 / 4967151	5.40 (2.93-9.95)	Yes
B-cell Lymphoma Sub-cohort					
Epcoritamab	CRS	459 / 1463	4213 / 62055	6.28 (5.60-7.04)	Yes
	ICANS	119 / 1463	1791 / 62055	2.99 (2.46-3.63)	Yes
	Broadly Defined Neurotoxicity	171 / 1463	4697 / 62055	1.62 (1.38-1.91)	Yes
	Infection Composite	414 / 1463	13702 / 62055	1.39 (1.24-1.56)	Yes
	Serious Infection	302 / 1463	8261 / 62055	1.70 (1.49-1.93)	Yes
	Pneumonia/LRI	123 / 1463	4075 / 62055	1.31 (1.09-1.58)	Yes
	Sepsis	71 / 1463	2879 / 62055	1.05 (0.83-1.34)	No
	COVID-19	66 / 1463	3282 / 62055	0.85 (0.67-1.09)	No
	Viral/Opportunistic Infection	134 / 1463	1496 / 62055	4.09 (3.40-4.92)	Yes
	Fungal Infection	23 / 1463	1055 / 62055	0.94 (0.62-1.42)	No
	PJP	10 / 1463	310 / 62055	1.44 (0.78-2.66)	No
	HBV Reactivation	2 / 1463	258 / 62055	0.41 (0.12-1.42)	No
	PML	7 / 1463	290 / 62055	1.09 (0.53-2.26)	No
	Hypogammaglobulinaemia	52 / 1463	674 / 62055	3.38 (2.54-4.50)	Yes
	Neutropenia	103 / 1463	6421 / 62055	0.66 (0.54-0.81)	No
	Lymphopenia	12 / 1463	737 / 62055	0.72 (0.41-1.25)	No
	Febrile Neutropenia	22 / 1463	3089 / 62055	0.30 (0.20-0.45)	No
Glofitamab	CRS	253 / 944	4419 / 62574	4.82 (4.16-5.59)	Yes
	ICANS	47 / 944	1863 / 62574	1.72 (1.28-2.32)	Yes
	Broadly Defined Neurotoxicity	71 / 944	4797 / 62574	0.99 (0.77-1.26)	No
	Infection Composite	195 / 944	13921 / 62574	0.91 (0.78-1.07)	No
	Serious Infection	113 / 944	8450 / 62574	0.87 (0.72-1.06)	No
	Pneumonia/LRI	66 / 944	4132 / 62574	1.07 (0.83-1.38)	No
	Sepsis	37 / 944	2913 / 62574	0.85 (0.61-1.18)	No
	COVID-19	44 / 944	3304 / 62574	0.89 (0.66-1.20)	No
	Viral/Opportunistic Infection	29 / 944	1601 / 62574	1.23 (0.85-1.78)	No
	Fungal Infection	14 / 944	1064 / 62574	0.90 (0.53-1.52)	No

Drug	Safety Endpoint	Drug Exposure (Events / Total)	Comparator Exposure (Events / Total)	ROR (95% CI)	Signal Detected
	PJP	2 / 944	318 / 62574	0.52 (0.15-1.80)	No
	HBV Reactivation	0 / 944	260 / 62574	0.13 (0.01-2.03)	No
	PML	3 / 944	294 / 62574	0.79 (0.27-2.26)	No
	Hypogammaglobulinaemia	12 / 944	714 / 62574	1.16 (0.66-2.04)	No
	Neutropenia	58 / 944	6466 / 62574	0.57 (0.44-0.75)	No
	Lymphopenia	6 / 944	743 / 62574	0.58 (0.27-1.25)	No
	Febrile Neutropenia	11 / 944	3100 / 62574	0.24 (0.13-0.42)	No
Mosunetuzumab	CRS	110 / 472	4562 / 63046	3.91 (3.15-4.84)	Yes
	ICANS	4 / 472	1906 / 63046	0.31 (0.12-0.78)	No
	Broadly Defined Neurotoxicity	16 / 472	4852 / 63046	0.43 (0.27-0.71)	No
	Infection Composite	152 / 472	13964 / 63046	1.67 (1.38-2.03)	Yes
	Serious Infection	96 / 472	8467 / 63046	1.65 (1.32-2.07)	Yes
	Pneumonia/LRI	60 / 472	4138 / 63046	2.09 (1.59-2.74)	Yes
	Sepsis	22 / 472	2928 / 63046	1.02 (0.67-1.57)	No
	COVID-19	60 / 472	3288 / 63046	2.67 (2.03-3.50)	Yes
	Viral/Opportunistic Infection	18 / 472	1612 / 63046	1.55 (0.97-2.48)	No
	Fungal Infection	9 / 472	1069 / 63046	1.19 (0.62-2.26)	No
	PJP	2 / 472	318 / 63046	1.05 (0.30-3.64)	No
	HBV Reactivation	1 / 472	259 / 63046	0.77 (0.15-3.84)	No
	PML	0 / 472	297 / 63046	0.22 (0.01-3.58)	No
	Hypogammaglobulinaemia	8 / 472	718 / 63046	1.59 (0.80-3.14)	No
	Neutropenia	31 / 472	6493 / 63046	0.62 (0.43-0.89)	No
	Lymphopenia	1 / 472	748 / 63046	0.27 (0.05-1.32)	No
	Febrile Neutropenia	10 / 472	3101 / 63046	0.44 (0.24-0.81)	No

Data are presented as events/total reports. ROR, reporting odds ratio; CI, confidence interval; CRS, cytokine release syndrome; ICANS, immune effector cell-associated neurotoxicity syndrome; LRI, lower respiratory infection; PJP, Pneumocystis jirovecii pneumonia; HBV, hepatitis B virus; PML, progressive multifocal leukoencephalopathy; FAERS, FDA Adverse Event Reporting System. Signal detected indicates whether the prespecified disproportionality criteria were met.

Supplementary Table S5

Endpoint	Events, n (%)	Crude ROR (95% CI)	Primary aROR (95% CI)
**Epcoritamab (N = 1463)**			
CRS	459 (31.4%)	6.28 (5.60–7.05)	4.70 (4.15–5.32)
ICANS	119 (8.1%)	2.99 (2.47–3.63)	2.28 (1.87–2.80)
Broadly defined neurotoxicity	185 (12.6%)	1.73 (1.48–2.02)	1.70 (1.45–2.01)
Infection composite	374 (25.6%)	1.44 (1.28–1.62)	1.35 (1.20–1.53)
Serious infection proxy	296 (20.2%)	1.70 (1.49–1.93)	1.52 (1.33–1.74)
Pneumonia/lower respiratory infection	115 (7.9%)	1.29 (1.06–1.56)	1.20 (0.99–1.47)
COVID-19	69 (4.7%)	0.85 (0.67–1.09)	0.97 (0.76–1.25)
CMV	120 (8.2%)	5.81 (4.77–7.07)	4.54 (3.66–5.62)
Narrow opportunistic infection	153 (10.5%)	2.36 (1.99–2.80)	2.20 (1.84–2.63)
Pneumocystis jirovecii pneumonia (PJP)	10 (0.7%)	1.44 (0.78–2.66)	1.52 (0.79–2.91)
Progressive multifocal leukoencephalopathy (PML)	7 (0.5%)	1.10 (0.53–2.27)	0.96 (0.44–2.06)
Hypogammaglobulinaemia	52 (3.6%)	3.39 (2.54–4.51)	2.52 (1.86–3.41)
Neutropenia	103 (7.0%)	0.66 (0.54–0.81)	0.50 (0.41–0.62)
Febrile neutropenia	22 (1.5%)	0.30 (0.20–0.45)	0.24 (0.16–0.37)
**Glofitamab (N = 944)**			
CRS	253 (26.8%)	4.83 (4.17–5.59)	3.43 (2.94–4.00)
ICANS	47 (5.0%)	1.72 (1.28–2.32)	1.26 (0.93–1.70)
Broadly defined neurotoxicity	71 (7.5%)	0.96 (0.75–1.22)	0.90 (0.70–1.15)
Infection composite	156 (16.5%)	0.82 (0.69–0.98)	0.78 (0.65–0.93)
Serious infection proxy	113 (12.0%)	0.90 (0.74–1.09)	0.81 (0.66–0.99)
Pneumonia/lower respiratory infection	58 (6.1%)	0.99 (0.76–1.29)	0.97 (0.74–1.27)
COVID-19	44 (4.7%)	0.85 (0.63–1.15)	1.05 (0.77–1.43)
CMV	17 (1.8%)	1.11 (0.69–1.79)	0.77 (0.48–1.26)
Narrow opportunistic infection	29 (3.1%)	0.63 (0.43–0.90)	0.53 (0.36–0.77)
Pneumocystis jirovecii pneumonia (PJP)	2 (0.2%)	0.52 (0.15–1.80)	0.43 (0.11–1.74)
Progressive multifocal leukoencephalopathy (PML)	3 (0.3%)	0.79 (0.27–2.26)	0.70 (0.22–2.20)
Hypogammaglobulinaemia	12 (1.3%)	1.16 (0.66–2.04)	0.78 (0.44–1.39)
Neutropenia	58 (6.1%)	0.57 (0.44–0.75)	0.41 (0.32–0.54)
Febrile neutropenia	11 (1.2%)	0.24 (0.13–0.42)	0.17 (0.09–0.31)
**Mosunetuzumab (N = 472)**			
CRS	110 (23.3%)	3.91 (3.15–4.85)	6.23 (4.97–7.81)
ICANS	4 (0.8%)	0.31 (0.12–0.78)	0.48 (0.18–1.29)
Broadly defined neurotoxicity	16 (3.4%)	0.42 (0.26–0.69)	0.49 (0.30–0.81)
Infection composite	138 (29.2%)	1.73 (1.42–2.11)	1.54 (1.25–1.88)
Serious infection proxy	96 (20.3%)	1.70 (1.36–2.13)	1.65 (1.31–2.08)
Pneumonia/lower respiratory infection	59 (12.5%)	2.17 (1.65–2.85)	1.74 (1.32–2.31)
COVID-19	60 (12.7%)	2.55 (1.94–3.35)	1.66 (1.25–2.21)
CMV	9 (1.9%)	1.20 (0.63–2.30)	1.29 (0.66–2.53)
Narrow opportunistic infection	20 (4.2%)	0.88 (0.57–1.38)	0.93 (0.59–1.46)
Pneumocystis jirovecii pneumonia (PJP)	2 (0.4%)	1.05 (0.30–3.65)	0.62 (0.15–2.52)
Progressive multifocal leukoencephalopathy (PML)	0 (0.0%)	0.22 (0.01–3.58)	Not estimable
Hypogammaglobulinaemia	8 (1.7%)	1.59 (0.80–3.14)	1.60 (0.78–3.29)
Neutropenia	31 (6.6%)	0.62 (0.43–0.89)	0.83 (0.57–1.19)
Febrile neutropenia	10 (2.1%)	0.44 (0.24–0.81)	0.61 (0.32–1.14)

Supplementary Table S6. PT-level disproportionality results after exclusion of indication, disease-progression, administrative, and outcome terms.

Drug	Preferred Term (PT)	Drug Exposure (Events / Total)	Comparator Exposure (Events / Total)	ROR (95% CI)	PRR (Chi-square)	IC (IC025)	EBGM (EB05)	Four-algorithm positive signal
Epcoritamab	Pyrexia	136 / 1463	2706 / 62055	2.25 (1.88-2.70)	2.13 (81.45)	1.05 (0.62)	2.07 (1.62)	No
	Cytokine Release Syndrome	459 / 1463	4214 / 62055	6.28 (5.60-7.04)	4.62 (1267.30)	2.09 (1.78)	4.25 (3.56)	Yes
	Sepsis	45 / 1463	1334 / 62055	1.46 (1.08-1.97)	1.43 (5.77)	0.50 (-0.15)	1.41 (0.97)	No
	Hypophagia	4 / 1463	88 / 62055	2.16 (0.84-5.57)	1.93 (1.71)	0.78 (-1.42)	1.72 (0.48)	No
	Bronchitis	4 / 1463	87 / 62055	2.18 (0.84-5.64)	1.95 (1.77)	0.79 (-1.41)	1.73 (0.48)	No
	Febrile Neutropenia	22 / 1463	3089 / 62055	0.30 (0.20-0.45)	0.30 (37.03)	-1.68 (-2.36)	0.31 (0.21)	No
	Hypotension	37 / 1463	802 / 62055	2.01 (1.44-2.80)	1.96 (16.77)	0.92 (0.13)	1.89 (1.20)	No
	Acute Kidney Injury	9 / 1463	1092 / 62055	0.36 (0.19-0.69)	0.35 (10.99)	-1.45 (-2.52)	0.37 (0.20)	No
	Abdominal Pain	22 / 1463	515 / 62055	1.86 (1.22-2.85)	1.81 (7.74)	0.81 (-0.18)	1.75 (0.98)	No
	Tachycardia	9 / 1463	312 / 62055	1.29 (0.68-2.47)	1.22 (0.36)	0.27 (-1.09)	1.20 (0.55)	No
	Hypoxia	21 / 1463	354 / 62055	2.59 (1.67-4.02)	2.52 (18.22)	1.23 (0.12)	2.35 (1.23)	No
	Chills	7 / 1463	624 / 62055	0.51 (0.25-1.04)	0.48 (4.04)	-1.00 (-2.27)	0.50 (0.24)	No
	White Blood Cell Count Decreased	18 / 1463	1362 / 62055	0.57 (0.36-0.91)	0.56 (6.26)	-0.80 (-1.63)	0.57 (0.35)	No
	Pneumonia	48 / 1463	2190 / 62055	0.94 (0.70-1.25)	0.93 (0.26)	-0.10 (-0.67)	0.93 (0.67)	No
	Confusional State	12 / 1463	394 / 62055	1.35 (0.77-2.37)	1.29 (0.77)	0.34 (-0.86)	1.27 (0.63)	No
	Cytomegalovirus Infection Reactivation	40 / 1463	322 / 62055	5.45 (3.91-7.58)	5.27 (123.80)	2.20 (1.15)	4.58 (2.49)	Yes
	Covid-19 Pneumonia	15 / 1463	701 / 62055	0.94 (0.56-1.55)	0.91 (0.14)	-0.13 (-1.13)	0.91 (0.51)	No
	Bronchopulmonary Aspergillosis	3 / 1463	242 / 62055	0.61 (0.21-1.76)	0.53 (1.27)	-0.81 (-2.71)	0.57 (0.19)	No
	Hyponatraemia	11 / 1463	277 / 62055	1.76 (0.97-3.19)	1.68 (2.96)	0.69 (-0.66)	1.61 (0.74)	No
	Dehydration	4 / 1463	290 / 62055	0.66 (0.26-1.67)	0.58 (1.17)	-0.69 (-2.39)	0.62 (0.23)	No
	Blood Pressure Decreased	7 / 1463	141 / 62055	2.25 (1.08-4.71)	2.11 (3.88)	0.94 (-0.82)	1.92 (0.69)	No
	Pseudomonal Sepsis	3 / 1463	51 / 62055	2.88 (0.98-8.53)	2.50 (2.54)	1.00 (-1.62)	2.01 (0.44)	No
	Pneumonia Bacterial	16 / 1463	144 / 62055	4.88 (2.93-8.15)	4.71 (42.23)	1.98 (0.43)	3.94 (1.60)	No
	Fatigue	23 / 1463	1881 / 62055	0.52 (0.35-0.79)	0.52 (10.46)	-0.92 (-1.64)	0.53 (0.35)	No
	Back Pain	9 / 1463	355 / 62055	1.13 (0.59-2.16)	1.07 (0.05)	0.10 (-1.22)	1.07 (0.50)	No
	Tumour Lysis Syndrome	14 / 1463	601 / 62055	1.02 (0.61-1.73)	0.99 (0.00)	-0.02 (-1.06)	0.99 (0.54)	No
	Asthenia	15 / 1463	1111 / 62055	0.59 (0.35-0.97)	0.57 (4.80)	-0.77 (-1.68)	0.59 (0.35)	No
	Diarrhoea	10 / 1463	1906 / 62055	0.23 (0.12-0.42)	0.22 (27.86)	-2.09 (-3.06)	0.23 (0.13)	No
	Infection	29 / 1463	1283 / 62055	0.97 (0.67-1.41)	0.96 (0.05)	-0.06 (-0.79)	0.96 (0.63)	No
	Vomiting	11 / 1463	941 / 62055	0.51 (0.29-0.92)	0.50 (5.66)	-0.96 (-1.99)	0.51 (0.28)	No
	Progressive Multifocal Leukoencephalopathy	7 / 1463	290 / 62055	1.09 (0.53-2.26)	1.02 (0.00)	0.03 (-1.44)	1.02 (0.43)	No
	Somnolence	7 / 1463	245 / 62055	1.30 (0.63-2.69)	1.21 (0.25)	0.25 (-1.28)	1.19 (0.49)	No
	Pneumonia Cytomegaloviral	6 / 1463	52 / 62055	5.27 (2.33-11.92)	4.89 (16.68)	1.82 (-0.54)	3.54 (0.90)	No
	Ventricular Tachycardia	3 / 1463	72 / 62055	2.05 (0.70-6.00)	1.77 (0.96)	0.65 (-1.77)	1.57 (0.38)	No
	Atrial Fibrillation	14 / 1463	690 / 62055	0.89 (0.53-1.50)	0.86 (0.31)	-0.20 (-1.22)	0.87 (0.48)	No
	Tumour Flare	10 / 1463	68 / 62055	6.54 (3.41-12.54)	6.24 (38.39)	2.19 (0.13)	4.57 (1.38)	No
	Sinusitis	3 / 1463	159 / 62055	0.93 (0.32-2.69)	0.80 (0.15)	-0.27 (-2.32)	0.83 (0.25)	No
	Anaemia	27 / 1463	1834 / 62055	0.63 (0.43-0.92)	0.62 (6.19)	-0.66 (-1.35)	0.63 (0.42)	No
	Neutropenia	38 / 1463	2802 / 62055	0.57 (0.41-0.79)	0.57 (12.31)	-0.78 (-1.35)	0.58 (0.42)	No
	Covid-19	51 / 1463	2544 / 62055	0.85 (0.64-1.13)	0.85 (1.37)	-0.23 (-0.76)	0.85 (0.62)	No
	Dizziness	5 / 1463	572 / 62055	0.41 (0.17-0.94)	0.37 (5.34)	-1.33 (-2.75)	0.40 (0.17)	No
	Disorientation	5 / 1463	118 / 62055	1.97 (0.84-4.64)	1.80 (1.70)	0.72 (-1.24)	1.65 (0.53)	No
	Septic Shock	16 / 1463	939 / 62055	0.74 (0.45-1.21)	0.72 (1.70)	-0.45 (-1.36)	0.73 (0.43)	No
	Urinary Tract Infection	8 / 1463	485 / 62055	0.74 (0.38-1.46)	0.70 (1.02)	-0.48 (-1.75)	0.72 (0.34)	No
	Pneumonitis	5 / 1463	257 / 62055	0.91 (0.39-2.11)	0.82 (0.18)	-0.25 (-1.89)	0.84 (0.33)	No
	Immune Effector Cell-Associated	119 / 1463	1791 / 62055	2.99 (2.46-3.63)	2.82 (135.00)	1.43 (0.93)	2.69 (2.01)	Yes

Drug	Preferred Term (PT)	Drug Exposure (Events / Total)	Comparator Exposure (Events / Total)	ROR (95% CI)	PRR (Chi-square)	IC (IC025)	EBGM (EB05)	Four-algorithm positive signal
	Neurotoxicity Syndrome							
	Pleural Effusion	18 / 1463	460 / 62055	1.71 (1.07-2.73)	1.66 (4.58)	0.69 (-0.38)	1.61 (0.87)	No
	Acute Respiratory Failure	3 / 1463	197 / 62055	0.75 (0.26-2.16)	0.65 (0.57)	-0.55 (-2.51)	0.69 (0.22)	No
	Multiple Organ Dysfunction Syndrome	9 / 1463	533 / 62055	0.75 (0.40-1.43)	0.72 (1.00)	-0.45 (-1.66)	0.73 (0.36)	No
	Myelodysplastic Syndrome	3 / 1463	568 / 62055	0.26 (0.09-0.74)	0.22 (8.09)	-1.96 (-3.66)	0.26 (0.10)	No
	Enterovirus Infection	3 / 1463	27 / 62055	5.41 (1.77-16.46)	4.71 (7.90)	1.55 (-1.44)	2.94 (0.51)	No
	Respiratory Tract Infection	4 / 1463	159 / 62055	1.20 (0.47-3.06)	1.07 (0.02)	0.08 (-1.83)	1.06 (0.35)	No
	Therapy Non-Responder	4 / 1463	541 / 62055	0.35 (0.14-0.89)	0.31 (6.02)	-1.54 (-3.08)	0.34 (0.14)	No
	Blood Lactate Dehydrogenase Increased	26 / 1463	568 / 62055	1.99 (1.35-2.95)	1.94 (11.46)	0.90 (-0.03)	1.87 (1.09)	No
	C-Reactive Protein Increased	25 / 1463	400 / 62055	2.73 (1.82-4.09)	2.65 (24.36)	1.31 (0.27)	2.48 (1.35)	No
	Bacteraemia	13 / 1463	235 / 62055	2.44 (1.41-4.24)	2.35 (9.55)	1.12 (-0.25)	2.17 (0.98)	No
	Aphasia	3 / 1463	136 / 62055	1.09 (0.38-3.15)	0.94 (0.01)	-0.08 (-2.19)	0.95 (0.28)	No
	Haemophagocytic Lymphohistiocytosis	19 / 1463	366 / 62055	2.27 (1.44-3.59)	2.20 (11.92)	1.06 (-0.07)	2.08 (1.08)	No
	Hypertransaminasaemia	3 / 1463	131 / 62055	1.13 (0.39-3.27)	0.97 (0.00)	-0.04 (-2.16)	0.98 (0.28)	No
	Rash	15 / 1463	1294 / 62055	0.50 (0.30-0.83)	0.49 (7.96)	-0.98 (-1.86)	0.51 (0.30)	No
	Injection Site Reaction	10 / 1463	32 / 62055	13.79 (6.86-27.70)	13.26 (86.38)	2.84 (0.35)	7.16 (1.68)	No
	Neurotoxicity	12 / 1463	757 / 62055	0.70 (0.40-1.22)	0.67 (1.91)	-0.54 (-1.58)	0.69 (0.38)	No
	Cytomegalovirus Infection	37 / 1463	340 / 62055	4.76 (3.39-6.70)	4.62 (95.08)	2.03 (0.99)	4.08 (2.23)	Yes
	Pneumocystis Jirovecii Pneumonia	10 / 1463	272 / 62055	1.64 (0.88-3.04)	1.56 (1.94)	0.59 (-0.79)	1.50 (0.67)	No
	Pseudomonas Infection	6 / 1463	113 / 62055	2.43 (1.10-5.38)	2.25 (3.97)	1.00 (-0.92)	2.01 (0.66)	No
	Fall	14 / 1463	491 / 62055	1.25 (0.74-2.12)	1.21 (0.50)	0.26 (-0.84)	1.20 (0.63)	No
	Skin Infection	3 / 1463	71 / 62055	2.08 (0.71-6.08)	1.79 (1.01)	0.67 (-1.76)	1.59 (0.39)	No
	Chronic Obstructive Pulmonary Disease	3 / 1463	75 / 62055	1.97 (0.67-5.75)	1.70 (0.83)	0.61 (-1.79)	1.52 (0.38)	No
	Pneumonia Viral	3 / 1463	55 / 62055	2.68 (0.91-7.89)	2.31 (2.12)	0.93 (-1.65)	1.91 (0.43)	No
	Cytomegalovirus Viraemia	18 / 1463	103 / 62055	7.66 (4.66-12.60)	7.41 (85.16)	2.49 (0.80)	5.63 (2.10)	Yes
	Shock	3 / 1463	91 / 62055	1.62 (0.56-4.73)	1.40 (0.33)	0.39 (-1.91)	1.31 (0.34)	No
	Pulmonary Embolism	3 / 1463	311 / 62055	0.47 (0.17-1.36)	0.41 (2.55)	-1.14 (-2.96)	0.45 (0.16)	No
	Platelet Count Decreased	42 / 1463	1315 / 62055	1.38 (1.01-1.88)	1.35 (3.86)	0.42 (-0.24)	1.34 (0.91)	No
	Blood Creatinine Increased	8 / 1463	265 / 62055	1.36 (0.69-2.70)	1.28 (0.48)	0.32 (-1.13)	1.25 (0.54)	No
	Escherichia Infection	5 / 1463	100 / 62055	2.33 (0.98-5.49)	2.12 (2.83)	0.91 (-1.13)	1.89 (0.57)	No
	Pneumonia Fungal	5 / 1463	142 / 62055	1.64 (0.70-3.85)	1.49 (0.79)	0.50 (-1.37)	1.42 (0.48)	No
	Interstitial Lung Disease	6 / 1463	373 / 62055	0.74 (0.34-1.60)	0.68 (0.88)	-0.51 (-1.95)	0.70 (0.30)	No
	Staphylococcal Infection	5 / 1463	146 / 62055	1.59 (0.68-3.74)	1.45 (0.68)	0.47 (-1.39)	1.38 (0.47)	No
	Dyspnoea	11 / 1463	1042 / 62055	0.46 (0.26-0.83)	0.45 (7.54)	-1.11 (-2.12)	0.47 (0.26)	No
	Inappropriate Schedule Of Product Administration	22 / 1463	149 / 62055	6.46 (4.14-10.10)	6.26 (85.01)	2.34 (0.87)	5.07 (2.16)	Yes
	Pancreatitis	3 / 1463	123 / 62055	1.20 (0.41-3.48)	1.03 (0.00)	0.04 (-2.11)	1.03 (0.29)	No
	Depressed Level Of Consciousness	7 / 1463	90 / 62055	3.53 (1.67-7.44)	3.30 (10.42)	1.46 (-0.54)	2.74 (0.86)	No
	Staphylococcal Bacteraemia	5 / 1463	117 / 62055	1.99 (0.84-4.68)	1.81 (1.75)	0.73 (-1.23)	1.66 (0.53)	No
	Oxygen Saturation Decreased	8 / 1463	229 / 62055	1.57 (0.79-3.13)	1.48 (1.22)	0.51 (-1.00)	1.43 (0.59)	No
	Headache	11 / 1463	647 / 62055	0.75 (0.42-1.35)	0.72 (1.18)	-0.45 (-1.54)	0.73 (0.39)	No
	Gastrointestinal Haemorrhage	9 / 1463	290 / 62055	1.39 (0.73-2.66)	1.32 (0.67)	0.36 (-1.02)	1.29 (0.57)	No
	Oedema	5 / 1463	139 / 62055	1.67 (0.71-3.93)	1.53 (0.88)	0.53 (-1.36)	1.44 (0.48)	No
	Productive Cough	3 / 1463	108 / 62055	1.37 (0.47-3.97)	1.18 (0.08)	0.20 (-2.02)	1.15 (0.32)	No
	Swelling	7 / 1463	131 / 62055	2.42 (1.16-5.07)	2.27 (4.71)	1.03 (-0.77)	2.04 (0.72)	No
	Hypoaesthesia	4 / 1463	206 / 62055	0.92 (0.36-2.35)	0.82 (0.15)	-0.25 (-2.06)	0.84 (0.29)	No
	Neutrophil Count Decreased	48 / 1463	1025 / 62055	2.04 (1.52-2.73)	1.99 (22.84)	0.94 (0.25)	1.92 (1.28)	No
	Nausea	14 / 1463	1475 / 62055	0.41 (0.24-0.69)	0.40 (12.59)	-1.26 (-2.15)	0.42 (0.25)	No
	Loss Of Consciousness	6 / 1463	177 / 62055	1.55 (0.71-3.41)	1.44 (0.78)	0.46 (-1.25)	1.38 (0.51)	No

Drug	Preferred Term (PT)	Drug Exposure (Events / Total)	Comparator Exposure (Events / Total)	ROR (95% CI)	PRR (Chi-square)	IC (IC025)	EBGM (EB05)	Four-algorithm positive signal
	Rhinovirus Infection	4 / 1463	74 / 62055	2.56 (0.99-6.65)	2.29 (2.77)	0.97 (-1.32)	1.96 (0.52)	No
	Pain In Extremity	5 / 1463	271 / 62055	0.86 (0.37-2.00)	0.78 (0.30)	-0.32 (-1.94)	0.80 (0.31)	No
	Myalgia	4 / 1463	243 / 62055	0.78 (0.31-1.99)	0.70 (0.52)	-0.46 (-2.21)	0.73 (0.26)	No
	White Blood Cell Count Increased	3 / 1463	146 / 62055	1.01 (0.35-2.93)	0.87 (0.06)	-0.17 (-2.25)	0.89 (0.27)	No
	Lymphadenopathy	11 / 1463	454 / 62055	1.07 (0.60-1.93)	1.03 (0.01)	0.04 (-1.15)	1.03 (0.51)	No
	Adrenal Insufficiency	3 / 1463	78 / 62055	1.89 (0.65-5.53)	1.63 (0.71)	0.56 (-1.81)	1.48 (0.37)	No
	Hypertension	7 / 1463	445 / 62055	0.71 (0.34-1.47)	0.67 (1.15)	-0.54 (-1.88)	0.69 (0.32)	No
	Cytopenia	3 / 1463	641 / 62055	0.23 (0.08-0.66)	0.20 (9.76)	-2.13 (-3.81)	0.23 (0.09)	No
	Cardiac Failure	12 / 1463	474 / 62055	1.12 (0.64-1.97)	1.07 (0.06)	0.10 (-1.05)	1.07 (0.55)	No
	Toxicity To Various Agents	7 / 1463	549 / 62055	0.58 (0.28-1.19)	0.54 (2.72)	-0.83 (-2.12)	0.56 (0.27)	No
	General Physical Health Deterioration	18 / 1463	451 / 62055	1.75 (1.09-2.79)	1.69 (4.95)	0.71 (-0.36)	1.64 (0.88)	No
	Pleurisy	3 / 1463	3 / 62055	42.49 (9.65-187.10)	42.42 (60.67)	2.46 (-1.39)	5.48 (0.58)	No
	Chest Pain	3 / 1463	226 / 62055	0.65 (0.23-1.88)	0.56 (1.01)	-0.72 (-2.64)	0.61 (0.20)	No
	Constipation	12 / 1463	734 / 62055	0.72 (0.41-1.26)	0.69 (1.62)	-0.50 (-1.54)	0.71 (0.39)	No
	Cellulitis	4 / 1463	219 / 62055	0.87 (0.34-2.21)	0.78 (0.26)	-0.33 (-2.11)	0.80 (0.28)	No
	Blood Urea Increased	3 / 1463	32 / 62055	4.57 (1.52-13.79)	3.98 (6.11)	1.42 (-1.48)	2.68 (0.50)	No
	Procalcitonin Increased	3 / 1463	42 / 62055	3.50 (1.17-10.41)	3.03 (3.81)	1.19 (-1.55)	2.28 (0.46)	No
	Small Intestinal Perforation	3 / 1463	47 / 62055	3.13 (1.05-9.28)	2.71 (3.04)	1.08 (-1.59)	2.12 (0.45)	No
	Influenza	7 / 1463	250 / 62055	1.27 (0.61-2.63)	1.19 (0.20)	0.22 (-1.30)	1.17 (0.48)	No
	Blood Glucose Increased	4 / 1463	114 / 62055	1.67 (0.65-4.28)	1.49 (0.62)	0.48 (-1.58)	1.40 (0.42)	No
	Haemoglobin Decreased	4 / 1463	472 / 62055	0.40 (0.16-1.02)	0.36 (4.56)	-1.35 (-2.92)	0.39 (0.16)	No
	Renal Impairment	10 / 1463	372 / 62055	1.20 (0.65-2.21)	1.14 (0.17)	0.17 (-1.10)	1.13 (0.54)	No
	Diverticulitis	3 / 1463	61 / 62055	2.42 (0.82-7.10)	2.09 (1.62)	0.83 (-1.69)	1.77 (0.41)	No
	Disseminated Intravascular Coagulation	6 / 1463	114 / 62055	2.41 (1.09-5.33)	2.23 (3.88)	0.99 (-0.92)	1.99 (0.65)	No
	Altered State Of Consciousness	7 / 1463	53 / 62055	5.97 (2.77-12.84)	5.60 (23.40)	2.00 (-0.31)	3.98 (1.04)	No
	Blood Pressure Increased	3 / 1463	195 / 62055	0.76 (0.26-2.19)	0.65 (0.55)	-0.53 (-2.50)	0.69 (0.22)	No
	Seizure	4 / 1463	292 / 62055	0.65 (0.26-1.65)	0.58 (1.20)	-0.70 (-2.40)	0.61 (0.23)	No
	Aspartate Aminotransferase Increased	12 / 1463	422 / 62055	1.26 (0.71-2.21)	1.21 (0.41)	0.25 (-0.93)	1.19 (0.60)	No
	Hyperkalaemia	4 / 1463	95 / 62055	2.00 (0.78-5.16)	1.79 (1.33)	0.69 (-1.46)	1.62 (0.46)	No
	Ascites	8 / 1463	191 / 62055	1.89 (0.95-3.76)	1.78 (2.62)	0.74 (-0.84)	1.67 (0.67)	No
	Thrombocytopenia	29 / 1463	1885 / 62055	0.66 (0.45-0.95)	0.65 (5.45)	-0.60 (-1.27)	0.66 (0.45)	No
	Neuropathy Peripheral	5 / 1463	895 / 62055	0.26 (0.11-0.60)	0.24 (12.39)	-1.95 (-3.30)	0.26 (0.12)	No
	Acute Respiratory Distress Syndrome	3 / 1463	186 / 62055	0.80 (0.28-2.29)	0.68 (0.43)	-0.47 (-2.46)	0.72 (0.23)	No
	Pneumonia Aspiration	5 / 1463	80 / 62055	2.90 (1.22-6.90)	2.65 (4.84)	1.16 (-1.01)	2.24 (0.63)	No
	Myocardial Infarction	4 / 1463	166 / 62055	1.15 (0.45-2.93)	1.02 (0.00)	0.03 (-1.87)	1.02 (0.34)	No
	Cytomegalovirus Test Positive	20 / 1463	36 / 62055	24.13 (14.01-41.55)	23.56 (278.00)	3.52 (1.31)	11.45 (3.18)	Yes
	Hypoglycaemia	3 / 1463	42 / 62055	3.50 (1.17-10.41)	3.03 (3.81)	1.19 (-1.55)	2.28 (0.46)	No
	Metastases To Central Nervous System	6 / 1463	47 / 62055	5.82 (2.56-13.24)	5.42 (19.17)	1.92 (-0.51)	3.78 (0.92)	No
	Infusion Related Reaction	3 / 1463	569 / 62055	0.26 (0.09-0.74)	0.22 (8.12)	-1.97 (-3.66)	0.26 (0.10)	No
	Hydronephrosis	3 / 1463	98 / 62055	1.51 (0.52-4.38)	1.30 (0.20)	0.31 (-1.95)	1.24 (0.33)	No
	Decreased Appetite	13 / 1463	746 / 62055	0.76 (0.45-1.31)	0.74 (1.19)	-0.41 (-1.43)	0.75 (0.41)	No
	Pain	14 / 1463	523 / 62055	1.18 (0.70-1.99)	1.14 (0.22)	0.17 (-0.91)	1.13 (0.60)	No
	Haemorrhage	3 / 1463	203 / 62055	0.73 (0.25-2.10)	0.63 (0.66)	-0.58 (-2.54)	0.67 (0.21)	No
	Chest Discomfort	4 / 1463	264 / 62055	0.72 (0.28-1.83)	0.64 (0.79)	-0.57 (-2.29)	0.67 (0.25)	No
	Full Blood Count Decreased	8 / 1463	170 / 62055	2.12 (1.06-4.23)	2.00 (3.81)	0.89 (-0.75)	1.85 (0.71)	No
	Malaise	26 / 1463	442 / 62055	2.57 (1.73-3.81)	2.50 (22.16)	1.23 (0.23)	2.35 (1.31)	No
	Delirium	20 / 1463	158 / 62055	5.55 (3.49-8.81)	5.37 (63.30)	2.16 (0.70)	4.46 (1.91)	No
	Mass	6 / 1463	71 / 62055	3.87 (1.73-8.64)	3.58 (10.32)	1.52 (-0.66)	2.86 (0.81)	No

Drug	Preferred Term (PT)	Drug Exposure (Events / Total)	Comparator Exposure (Events / Total)	ROR (95% CI)	PRR (Chi-square)	IC (IC025)	EBGM (EB05)	Four-algorithm positive signal
	Hypogammaglobulinaemia	43 / 1463	530 / 62055	3.55 (2.60-4.86)	3.44 (69.51)	1.67 (0.79)	3.18 (1.91)	No
	Gamma-Glutamyltransferase Increased	3 / 1463	167 / 62055	0.89 (0.31-2.56)	0.76 (0.22)	-0.34 (-2.36)	0.79 (0.24)	No
	Shock Haemorrhagic	4 / 1463	77 / 62055	2.47 (0.95-6.39)	2.20 (2.50)	0.93 (-1.34)	1.90 (0.51)	No
	Interleukin-2 Receptor Increased	4 / 1463	5 / 62055	34.78 (10.00-121.00)	33.93 (71.04)	2.67 (-0.95)	6.36 (0.78)	No
	Oedema Peripheral	8 / 1463	286 / 62055	1.26 (0.64-2.50)	1.19 (0.23)	0.23 (-1.20)	1.17 (0.51)	No
	Pancytopenia	13 / 1463	1084 / 62055	0.52 (0.30-0.90)	0.51 (6.20)	-0.93 (-1.88)	0.52 (0.30)	No
	Escherichia Bacteraemia	3 / 1463	87 / 62055	1.70 (0.58-4.95)	1.46 (0.42)	0.44 (-1.88)	1.36 (0.35)	No
	Melaena	3 / 1463	41 / 62055	3.58 (1.20-10.67)	3.10 (3.99)	1.21 (-1.54)	2.31 (0.47)	No
	Respiratory Failure	11 / 1463	640 / 62055	0.76 (0.42-1.36)	0.73 (1.10)	-0.43 (-1.53)	0.74 (0.39)	No
	Nervous System Disorder	6 / 1463	150 / 62055	1.83 (0.83-4.03)	1.70 (1.65)	0.67 (-1.12)	1.59 (0.56)	No
	Liver Disorder	9 / 1463	130 / 62055	3.10 (1.60-6.00)	2.94 (10.77)	1.36 (-0.37)	2.57 (0.94)	No
	Blood Bilirubin Increased	4 / 1463	175 / 62055	1.09 (0.43-2.77)	0.97 (0.00)	-0.04 (-1.91)	0.97 (0.33)	No
	Cytomegalovirus Enterocolitis	8 / 1463	27 / 62055	13.17 (6.09-28.48)	12.57 (65.75)	2.70 (0.04)	6.51 (1.39)	No
	Tremor	3 / 1463	316 / 62055	0.47 (0.16-1.34)	0.40 (2.65)	-1.17 (-2.98)	0.45 (0.15)	No
	Palliative Care	5 / 1463	9 / 62055	24.63 (8.61-70.48)	23.56 (69.47)	2.74 (-0.60)	6.69 (0.96)	No
	Abdominal Distension	6 / 1463	137 / 62055	2.01 (0.91-4.42)	1.86 (2.28)	0.78 (-1.05)	1.71 (0.59)	No
	Spinal Stenosis	3 / 1463	15 / 62055	9.59 (3.00-30.63)	8.48 (16.51)	1.94 (-1.39)	3.83 (0.55)	No
	Femoral Neck Fracture	3 / 1463	10 / 62055	14.16 (4.22-47.53)	12.72 (24.94)	2.13 (-1.38)	4.38 (0.57)	No
	Dysarthria	3 / 1463	98 / 62055	1.51 (0.52-4.38)	1.30 (0.20)	0.31 (-1.95)	1.24 (0.33)	No
	Unevaluable Event	5 / 1463	100 / 62055	2.33 (0.98-5.49)	2.12 (2.83)	0.91 (-1.13)	1.89 (0.57)	No
	Therapeutic Product Effect Incomplete	8 / 1463	160 / 62055	2.25 (1.13-4.50)	2.12 (4.53)	0.96 (-0.70)	1.95 (0.74)	No
	Hepatic Function Abnormal	4 / 1463	275 / 62055	0.69 (0.27-1.76)	0.62 (0.94)	-0.62 (-2.33)	0.65 (0.24)	No
	Renal Failure	6 / 1463	456 / 62055	0.60 (0.28-1.31)	0.56 (2.09)	-0.78 (-2.17)	0.58 (0.26)	No
	Respiratory Disorder	4 / 1463	67 / 62055	2.83 (1.09-7.36)	2.53 (3.50)	1.07 (-1.27)	2.11 (0.54)	No
	Injection Site Rash	3 / 1463	6 / 62055	22.88 (6.23-83.98)	21.21 (38.52)	2.31 (-1.38)	4.95 (0.58)	No
	Injection Site Erythema	7 / 1463	8 / 62055	37.59 (14.06-100.50)	37.11 (131.20)	3.15 (-0.10)	8.87 (1.34)	No
	Injection Site Pruritus	4 / 1463	4 / 62055	42.52 (11.50-157.20)	42.42 (80.89)	2.72 (-0.95)	6.58 (0.78)	No
	Bacterial Infection	4 / 1463	211 / 62055	0.90 (0.35-2.30)	0.80 (0.19)	-0.28 (-2.08)	0.82 (0.29)	No
	Abdominal Mass	3 / 1463	33 / 62055	4.44 (1.47-13.36)	3.86 (5.82)	1.40 (-1.48)	2.63 (0.49)	No
	Decreased Immune Responsiveness	3 / 1463	34 / 62055	4.31 (1.43-12.95)	3.74 (5.54)	1.37 (-1.49)	2.59 (0.49)	No
	Cough	8 / 1463	567 / 62055	0.63 (0.32-1.25)	0.60 (2.15)	-0.69 (-1.93)	0.62 (0.30)	No
	Blood Immunoglobulin G Decreased	7 / 1463	95 / 62055	3.34 (1.59-7.04)	3.12 (9.44)	1.40 (-0.57)	2.63 (0.84)	No
	Hypercalcaemia	3 / 1463	108 / 62055	1.37 (0.47-3.97)	1.18 (0.08)	0.20 (-2.02)	1.15 (0.32)	No
	Pruritus	4 / 1463	667 / 62055	0.28 (0.11-0.72)	0.25 (8.78)	-1.83 (-3.33)	0.28 (0.12)	No
	Renal Disorder	3 / 1463	81 / 62055	1.82 (0.62-5.32)	1.57 (0.60)	0.52 (-1.84)	1.44 (0.36)	No
	Herpes Zoster	5 / 1463	250 / 62055	0.93 (0.40-2.17)	0.85 (0.13)	-0.21 (-1.86)	0.86 (0.33)	No
	Intestinal Perforation	9 / 1463	123 / 62055	3.27 (1.69-6.35)	3.10 (11.98)	1.42 (-0.34)	2.68 (0.96)	No
	Upper Respiratory Tract Infection	3 / 1463	157 / 62055	0.94 (0.33-2.72)	0.81 (0.13)	-0.26 (-2.31)	0.84 (0.25)	No
	Secondary Immunodeficiency	8 / 1463	26 / 62055	13.67 (6.30-29.65)	13.05 (68.11)	2.73 (0.05)	6.62 (1.40)	No
	Hypokalaemia	7 / 1463	503 / 62055	0.63 (0.31-1.30)	0.59 (1.98)	-0.71 (-2.02)	0.61 (0.29)	No
	Thrombosis	5 / 1463	189 / 62055	1.23 (0.53-2.88)	1.12 (0.07)	0.15 (-1.60)	1.11 (0.40)	No
	Herpes Zoster Disseminated	3 / 1463	7 / 62055	19.83 (5.57-70.59)	18.18 (34.10)	2.26 (-1.38)	4.79 (0.58)	No
	Gastrointestinal Perforation	4 / 1463	42 / 62055	4.50 (1.70-11.90)	4.04 (8.36)	1.53 (-1.10)	2.89 (0.63)	No
	Gastrointestinal Disorder	7 / 1463	276 / 62055	1.15 (0.56-2.38)	1.08 (0.04)	0.10 (-1.39)	1.07 (0.45)	No
	Myelosuppression	8 / 1463	1801 / 62055	0.20 (0.10-0.38)	0.19 (28.66)	-2.31 (-3.37)	0.20 (0.11)	No
	Arthralgia	7 / 1463	418 / 62055	0.76 (0.37-1.56)	0.71 (0.82)	-0.46 (-1.81)	0.73 (0.33)	No
	Lung Disorder	3 / 1463	117 / 62055	1.26 (0.44-3.66)	1.09 (0.02)	0.10 (-2.08)	1.07 (0.30)	No
	Coronavirus Infection	4 / 1463	123 / 62055	1.55 (0.60-3.97)	1.38 (0.41)	0.39 (-1.63)	1.31 (0.40)	No

Drug	Preferred Term (PT)	Drug Exposure (Events / Total)	Comparator Exposure (Events / Total)	ROR (95% CI)	PRR (Chi-square)	IC (IC025)	EBGM (EB05)	Four-algorithm positive signal
	Inflammation	4 / 1463	90 / 62055	2.11 (0.82-5.45)	1.89 (1.59)	0.76 (-1.43)	1.69 (0.47)	No
	Cardio-Respiratory Arrest	4 / 1463	69 / 62055	2.75 (1.06-7.14)	2.46 (3.28)	1.04 (-1.29)	2.06 (0.53)	No
	Erythema	6 / 1463	299 / 62055	0.92 (0.42-2.00)	0.85 (0.15)	-0.21 (-1.73)	0.86 (0.36)	No
	Alanine Aminotransferase Increased	14 / 1463	455 / 62055	1.35 (0.80-2.29)	1.30 (0.98)	0.36 (-0.76)	1.28 (0.67)	No
	Injection Site Pain	3 / 1463	22 / 62055	6.61 (2.14-20.40)	5.78 (10.45)	1.70 (-1.42)	3.25 (0.53)	No
	Lymphocyte Count Decreased	3 / 1463	458 / 62055	0.32 (0.11-0.92)	0.28 (5.64)	-1.67 (-3.40)	0.32 (0.12)	No
	Myopathy	3 / 1463	15 / 62055	9.59 (3.00-30.63)	8.48 (16.51)	1.94 (-1.39)	3.83 (0.55)	No
	Cholecystitis	3 / 1463	58 / 62055	2.54 (0.86-7.48)	2.19 (1.85)	0.88 (-1.67)	1.84 (0.42)	No
	Peritonitis	3 / 1463	58 / 62055	2.54 (0.86-7.48)	2.19 (1.85)	0.88 (-1.67)	1.84 (0.42)	No
	Hypothyroidism	3 / 1463	83 / 62055	1.78 (0.61-5.19)	1.53 (0.54)	0.50 (-1.85)	1.41 (0.36)	No
	Hypoalbuminaemia	20 / 1463	232 / 62055	3.78 (2.40-5.95)	3.66 (35.68)	1.70 (0.41)	3.25 (1.54)	No
	Weight Decreased	4 / 1463	552 / 62055	0.34 (0.14-0.87)	0.31 (6.25)	-1.56 (-3.11)	0.34 (0.14)	No
	Cardiac Arrest	5 / 1463	228 / 62055	1.02 (0.44-2.38)	0.93 (0.03)	-0.09 (-1.77)	0.94 (0.35)	No
	Fracture	3 / 1463	30 / 62055	4.87 (1.61-14.75)	4.24 (6.76)	1.47 (-1.46)	2.78 (0.50)	No
	Erythema Of Eyelid	3 / 1463	4 / 62055	33.05 (8.17-133.70)	31.81 (51.17)	2.40 (-1.39)	5.29 (0.58)	No
	Weight Increased	3 / 1463	163 / 62055	0.91 (0.31-2.62)	0.78 (0.18)	-0.30 (-2.34)	0.81 (0.25)	No
	Adenoviral Haemorrhagic Cystitis	3 / 1463	10 / 62055	14.16 (4.22-47.53)	12.72 (24.94)	2.13 (-1.38)	4.38 (0.57)	No
	Aspergillus Infection	4 / 1463	222 / 62055	0.86 (0.34-2.18)	0.76 (0.29)	-0.34 (-2.12)	0.79 (0.28)	No
	Therapeutic Product Effect Decreased	4 / 1463	40 / 62055	4.72 (1.78-12.52)	4.24 (9.02)	1.57 (-1.08)	2.97 (0.63)	No
	Systemic Candida	5 / 1463	125 / 62055	1.86 (0.79-4.38)	1.70 (1.38)	0.65 (-1.28)	1.57 (0.51)	No
	Marasmus	3 / 1463	8 / 62055	17.49 (5.03-60.81)	15.91 (30.48)	2.22 (-1.38)	4.65 (0.57)	No
	Splenomegaly	3 / 1463	140 / 62055	1.06 (0.36-3.05)	0.91 (0.03)	-0.12 (-2.21)	0.92 (0.27)	No
	Gastrointestinal Inflammation	3 / 1463	16 / 62055	9.01 (2.84-28.59)	7.95 (15.36)	1.90 (-1.39)	3.73 (0.55)	No
	Cerebral Infarction	3 / 1463	55 / 62055	2.68 (0.91-7.89)	2.31 (2.12)	0.93 (-1.65)	1.91 (0.43)	No
	Meningitis	3 / 1463	23 / 62055	6.33 (2.06-19.47)	5.53 (9.86)	1.67 (-1.42)	3.19 (0.53)	No
	Lymphopenia	9 / 1463	258 / 62055	1.56 (0.81-2.99)	1.48 (1.36)	0.52 (-0.92)	1.43 (0.62)	No
	Performance Status Decreased	3 / 1463	28 / 62055	5.22 (1.72-15.85)	4.54 (7.50)	1.53 (-1.45)	2.88 (0.51)	No
	Hepatic Failure	3 / 1463	168 / 62055	0.88 (0.30-2.54)	0.76 (0.23)	-0.34 (-2.36)	0.79 (0.24)	No
	Adverse Event	17 / 1463	142 / 62055	5.26 (3.19-8.66)	5.08 (49.85)	2.07 (0.53)	4.20 (1.71)	No
Glofitamab	Haematemesis	4 / 944	33 / 62574	8.93 (3.33-23.95)	8.04 (21.99)	2.10 (-0.96)	4.29 (0.72)	No
	Anaemia	34 / 944	1827 / 62574	1.26 (0.89-1.77)	1.23 (1.52)	0.29 (-0.42)	1.23 (0.81)	No
	Cytokine Release Syndrome	253 / 944	4420 / 62574	4.82 (4.16-5.59)	3.79 (531.50)	1.86 (1.48)	3.62 (2.90)	Yes
	Septic Shock	17 / 944	938 / 62574	1.24 (0.77-2.00)	1.20 (0.57)	0.25 (-0.75)	1.19 (0.67)	No
	Pyrexia	82 / 944	2760 / 62574	2.07 (1.65-2.61)	1.97 (39.78)	0.95 (0.42)	1.93 (1.42)	No
	Covid-19	38 / 944	2557 / 62574	1.00 (0.72-1.38)	0.98 (0.01)	-0.02 (-0.66)	0.98 (0.68)	No
	Bronchitis	4 / 944	87 / 62574	3.42 (1.32-8.83)	3.05 (5.27)	1.28 (-1.19)	2.43 (0.58)	No
	Hypercalcaemia	3 / 944	108 / 62574	2.14 (0.74-6.22)	1.84 (1.12)	0.70 (-1.75)	1.63 (0.39)	No
	Pain	9 / 944	528 / 62574	1.19 (0.63-2.27)	1.13 (0.13)	0.16 (-1.17)	1.12 (0.52)	No
	Vomiting	17 / 944	935 / 62574	1.24 (0.77-2.00)	1.21 (0.59)	0.26 (-0.74)	1.20 (0.67)	No
	Nausea	5 / 944	1484 / 62574	0.24 (0.10-0.56)	0.22 (13.78)	-2.04 (-3.38)	0.24 (0.11)	No
	Weight Decreased	4 / 944	552 / 62574	0.54 (0.21-1.36)	0.48 (2.25)	-0.96 (-2.60)	0.51 (0.20)	No
	Renal Impairment	5 / 944	377 / 62574	0.96 (0.41-2.24)	0.88 (0.08)	-0.17 (-1.82)	0.89 (0.34)	No
	Pneumonia	46 / 944	2192 / 62574	1.43 (1.06-1.92)	1.39 (5.13)	0.46 (-0.18)	1.38 (0.95)	No
	Pancreatitis	3 / 944	123 / 62574	1.88 (0.65-5.45)	1.62 (0.69)	0.56 (-1.82)	1.48 (0.37)	No
	Herpes Zoster	9 / 944	246 / 62574	2.57 (1.34-4.92)	2.42 (7.30)	1.15 (-0.50)	2.21 (0.85)	No
	C-Reactive Protein Increased	8 / 944	417 / 62574	1.35 (0.68-2.67)	1.27 (0.46)	0.32 (-1.14)	1.25 (0.54)	No
	Rash	8 / 944	1301 / 62574	0.43 (0.22-0.84)	0.41 (6.99)	-1.23 (-2.39)	0.43 (0.22)	No
	Gastrointestinal Haemorrhage	6 / 944	293 / 62574	1.47 (0.67-3.21)	1.36 (0.56)	0.40 (-1.29)	1.31 (0.49)	No

Drug	Preferred Term (PT)	Drug Exposure (Events / Total)	Comparator Exposure (Events / Total)	ROR (95% CI)	PRR (Chi-square)	IC (IC025)	EBGM (EB05)	Four-algorithm positive signal
	Colitis	4 / 944	250 / 62574	1.19 (0.47-3.03)	1.06 (0.01)	0.07 (-1.84)	1.05 (0.35)	No
	Neutropenia	41 / 944	2799 / 62574	0.98 (0.72-1.34)	0.97 (0.04)	-0.04 (-0.66)	0.97 (0.68)	No
	Covid-19 Pneumonia	7 / 944	709 / 62574	0.70 (0.34-1.44)	0.65 (1.28)	-0.57 (-1.91)	0.67 (0.31)	No
	Cardiac Failure	4 / 944	482 / 62574	0.62 (0.24-1.56)	0.55 (1.47)	-0.78 (-2.46)	0.58 (0.22)	No
	Respiratory Failure	12 / 944	639 / 62574	1.30 (0.74-2.28)	1.25 (0.57)	0.30 (-0.90)	1.23 (0.61)	No
	Multiple Organ Dysfunction Syndrome	7 / 944	535 / 62574	0.93 (0.45-1.91)	0.87 (0.14)	-0.19 (-1.60)	0.88 (0.39)	No
	Acute Kidney Injury	8 / 944	1093 / 62574	0.51 (0.26-1.00)	0.48 (4.42)	-0.99 (-2.18)	0.50 (0.25)	No
	Upper Respiratory Tract Infection	6 / 944	154 / 62574	2.80 (1.27-6.15)	2.58 (5.62)	1.18 (-0.83)	2.26 (0.70)	No
	Myelosuppression	16 / 944	1793 / 62574	0.60 (0.37-0.98)	0.59 (4.61)	-0.73 (-1.61)	0.60 (0.36)	No
	White Blood Cell Count Decreased	18 / 944	1362 / 62574	0.90 (0.56-1.43)	0.88 (0.32)	-0.18 (-1.08)	0.88 (0.52)	No
	Platelet Count Decreased	30 / 944	1327 / 62574	1.54 (1.07-2.22)	1.50 (4.97)	0.56 (-0.24)	1.48 (0.92)	No
	Neutrophil Count Decreased	9 / 944	1064 / 62574	0.59 (0.31-1.11)	0.56 (3.12)	-0.79 (-1.94)	0.58 (0.29)	No
	Cerebral Haemorrhage	5 / 944	104 / 62574	3.50 (1.48-8.27)	3.19 (7.17)	1.38 (-0.91)	2.59 (0.69)	No
	Tumour Lysis Syndrome	12 / 944	603 / 62574	1.38 (0.78-2.42)	1.32 (0.92)	0.38 (-0.84)	1.30 (0.64)	No
	Cytomegalovirus Infection	6 / 944	371 / 62574	1.16 (0.53-2.52)	1.07 (0.03)	0.09 (-1.50)	1.06 (0.42)	No
	Immune Effector Cell-Associated Neurotoxicity Syndrome	47 / 944	1863 / 62574	1.72 (1.28-2.32)	1.67 (12.77)	0.72 (0.05)	1.64 (1.11)	No
	Aspartate Aminotransferase Increased	15 / 944	419 / 62574	2.47 (1.48-4.12)	2.37 (11.58)	1.16 (-0.13)	2.23 (1.05)	No
	Alanine Aminotransferase Increased	18 / 944	451 / 62574	2.75 (1.72-4.39)	2.65 (17.85)	1.31 (0.08)	2.48 (1.21)	No
	Tumour Flare	19 / 944	59 / 62574	22.14 (13.22-37.07)	21.35 (279.10)	3.56 (1.27)	11.75 (3.11)	Yes
	Pneumonitis	5 / 944	257 / 62574	1.42 (0.61-3.31)	1.29 (0.32)	0.32 (-1.49)	1.25 (0.44)	No
	Ill-Defined Disorder	6 / 944	211 / 62574	2.04 (0.93-4.47)	1.89 (2.43)	0.80 (-1.03)	1.75 (0.60)	No
	Blood Bilirubin Increased	8 / 944	171 / 62574	3.30 (1.65-6.60)	3.10 (10.91)	1.43 (-0.44)	2.69 (0.91)	No
	Hypokalaemia	5 / 944	505 / 62574	0.72 (0.31-1.67)	0.66 (0.90)	-0.56 (-2.12)	0.68 (0.27)	No
	Leukopenia	8 / 944	716 / 62574	0.78 (0.40-1.55)	0.74 (0.73)	-0.41 (-1.69)	0.76 (0.36)	No
	Thrombocytopenia	26 / 944	1888 / 62574	0.93 (0.63-1.37)	0.91 (0.22)	-0.13 (-0.89)	0.92 (0.59)	No
	No Adverse Event	25 / 944	724 / 62574	2.37 (1.59-3.53)	2.29 (17.75)	1.13 (0.13)	2.19 (1.23)	No
	Blood Lactate Dehydrogenase Increased	8 / 944	586 / 62574	0.96 (0.49-1.89)	0.91 (0.08)	-0.13 (-1.48)	0.91 (0.42)	No
	Haemorrhage	5 / 944	201 / 62574	1.81 (0.78-4.24)	1.65 (1.25)	0.63 (-1.30)	1.54 (0.50)	No
	Leukocytosis	3 / 944	86 / 62574	2.69 (0.92-7.83)	2.31 (2.16)	0.94 (-1.64)	1.92 (0.43)	No
	Pulmonary Embolism	3 / 944	311 / 62574	0.74 (0.26-2.13)	0.64 (0.61)	-0.56 (-2.52)	0.68 (0.22)	No
	Pneumonia Fungal	4 / 944	143 / 62574	2.08 (0.81-5.33)	1.85 (1.53)	0.74 (-1.44)	1.68 (0.47)	No
	Febrile Neutropenia	11 / 944	3100 / 62574	0.24 (0.13-0.42)	0.23 (28.66)	-2.02 (-2.95)	0.25 (0.14)	No
	Sepsis	11 / 944	1368 / 62574	0.55 (0.31-0.99)	0.53 (4.56)	-0.87 (-1.91)	0.55 (0.30)	No
	Hypotension	13 / 944	826 / 62574	1.08 (0.63-1.86)	1.04 (0.02)	0.06 (-1.04)	1.04 (0.55)	No
	Therapy Non-Responder	5 / 944	540 / 62574	0.67 (0.29-1.56)	0.61 (1.22)	-0.65 (-2.19)	0.64 (0.26)	No
	Haematuria	4 / 944	88 / 62574	3.38 (1.31-8.73)	3.01 (5.15)	1.27 (-1.19)	2.41 (0.58)	No
	Infection	34 / 944	1278 / 62574	1.82 (1.29-2.56)	1.76 (11.18)	0.79 (-0.01)	1.73 (1.09)	No
	Urosepsis	3 / 944	69 / 62574	3.34 (1.14-9.80)	2.88 (3.54)	1.16 (-1.56)	2.23 (0.46)	No
	Deep Vein Thrombosis	3 / 944	310 / 62574	0.74 (0.26-2.14)	0.64 (0.60)	-0.56 (-2.52)	0.68 (0.22)	No
	Urinary Tract Infection	8 / 944	485 / 62574	1.16 (0.59-2.29)	1.09 (0.06)	0.12 (-1.28)	1.09 (0.48)	No
	Cardiac Arrest	5 / 944	228 / 62574	1.60 (0.68-3.73)	1.45 (0.69)	0.47 (-1.39)	1.39 (0.47)	No
	Oxygen Saturation Decreased	6 / 944	231 / 62574	1.86 (0.85-4.08)	1.72 (1.78)	0.69 (-1.10)	1.62 (0.57)	No
	Infusion Related Reaction	5 / 944	567 / 62574	0.64 (0.28-1.49)	0.58 (1.48)	-0.71 (-2.24)	0.61 (0.25)	No
	Tachycardia	4 / 944	317 / 62574	0.94 (0.37-2.38)	0.84 (0.13)	-0.23 (-2.04)	0.85 (0.30)	No
	Tachypnoea	5 / 944	101 / 62574	3.60 (1.52-8.52)	3.28 (7.57)	1.41 (-0.90)	2.65 (0.69)	No
	Acute Myeloid Leukaemia	5 / 944	396 / 62574	0.92 (0.40-2.13)	0.84 (0.16)	-0.23 (-1.87)	0.85 (0.33)	No
	Myelodysplastic Syndrome	4 / 944	567 / 62574	0.52 (0.21-1.32)	0.47 (2.43)	-1.00 (-2.63)	0.50 (0.19)	No

Drug	Preferred Term (PT)	Drug Exposure (Events / Total)	Comparator Exposure (Events / Total)	ROR (95% CI)	PRR (Chi-square)	IC (IC025)	EBGM (EB05)	Four-algorithm positive signal
	Hypertransaminasaemia	4 / 944	130 / 62574	2.29 (0.89-5.87)	2.04 (2.06)	0.85 (-1.38)	1.81 (0.49)	No
	Hypoxia	6 / 944	369 / 62574	1.17 (0.54-2.54)	1.08 (0.03)	0.10 (-1.50)	1.07 (0.42)	No
	Blood Creatinine Increased	6 / 944	267 / 62574	1.61 (0.74-3.52)	1.49 (0.95)	0.51 (-1.22)	1.43 (0.52)	No
	Arthralgia	4 / 944	421 / 62574	0.71 (0.28-1.79)	0.63 (0.87)	-0.60 (-2.32)	0.66 (0.24)	No
	Decreased Appetite	9 / 944	750 / 62574	0.84 (0.44-1.59)	0.80 (0.47)	-0.31 (-1.54)	0.81 (0.39)	No
	Diarrhoea	6 / 944	1910 / 62574	0.22 (0.10-0.48)	0.21 (18.57)	-2.16 (-3.38)	0.22 (0.11)	No
	Hypophosphataemia	5 / 944	137 / 62574	2.66 (1.13-6.25)	2.42 (4.03)	1.07 (-1.05)	2.11 (0.61)	No
	Back Pain	3 / 944	361 / 62574	0.64 (0.22-1.84)	0.55 (1.10)	-0.76 (-2.66)	0.59 (0.20)	No
	Neurotoxicity	6 / 944	763 / 62574	0.56 (0.26-1.22)	0.52 (2.65)	-0.88 (-2.25)	0.55 (0.24)	No
	Constipation	3 / 944	743 / 62574	0.31 (0.11-0.89)	0.27 (6.06)	-1.73 (-3.45)	0.30 (0.11)	No
	Lymphocyte Count Decreased	4 / 944	457 / 62574	0.65 (0.26-1.65)	0.58 (1.21)	-0.71 (-2.40)	0.61 (0.23)	No
	Fatigue	9 / 944	1895 / 62574	0.33 (0.17-0.62)	0.32 (13.77)	-1.60 (-2.66)	0.33 (0.18)	No
	Fall	5 / 944	500 / 62574	0.73 (0.31-1.69)	0.66 (0.86)	-0.54 (-2.11)	0.69 (0.28)	No
	Syncope	3 / 944	209 / 62574	1.11 (0.38-3.19)	0.95 (0.01)	-0.06 (-2.18)	0.96 (0.28)	No
	Abdominal Pain	5 / 944	532 / 62574	0.68 (0.29-1.58)	0.62 (1.14)	-0.62 (-2.17)	0.65 (0.26)	No
	Acute Myocardial Infarction	3 / 944	75 / 62574	3.08 (1.05-9.00)	2.65 (2.97)	1.08 (-1.59)	2.11 (0.45)	No
	Depressed Level Of Consciousness	5 / 944	92 / 62574	3.95 (1.67-9.37)	3.60 (8.93)	1.50 (-0.86)	2.83 (0.72)	No
	Dyspnoea	13 / 944	1040 / 62574	0.86 (0.50-1.47)	0.83 (0.46)	-0.26 (-1.30)	0.84 (0.46)	No
	Pleural Effusion	5 / 944	473 / 62574	0.77 (0.33-1.78)	0.70 (0.64)	-0.47 (-2.05)	0.72 (0.29)	No
	Liver Function Test Increased	4 / 944	60 / 62574	4.94 (1.89-12.91)	4.42 (9.93)	1.63 (-1.07)	3.10 (0.65)	No
	Speech Disorder	3 / 944	59 / 62574	3.91 (1.33-11.50)	3.37 (4.76)	1.30 (-1.51)	2.46 (0.48)	No
	Chills	8 / 944	623 / 62574	0.90 (0.46-1.78)	0.85 (0.21)	-0.22 (-1.54)	0.86 (0.40)	No
	Heart Rate Increased	3 / 944	142 / 62574	1.63 (0.56-4.71)	1.40 (0.34)	0.40 (-1.90)	1.32 (0.35)	No
	Disorientation	4 / 944	119 / 62574	2.50 (0.97-6.42)	2.23 (2.62)	0.95 (-1.33)	1.93 (0.51)	No
	Cytomegalovirus Infection Reactivation	7 / 944	355 / 62574	1.40 (0.68-2.89)	1.31 (0.50)	0.35 (-1.21)	1.28 (0.52)	No
	Influenza	3 / 944	254 / 62574	0.91 (0.32-2.62)	0.78 (0.18)	-0.30 (-2.34)	0.81 (0.25)	No
	Hypertension	5 / 944	447 / 62574	0.81 (0.35-1.89)	0.74 (0.45)	-0.39 (-1.99)	0.76 (0.30)	No
	Enterocolitis	3 / 944	88 / 62574	2.62 (0.90-7.65)	2.26 (2.04)	0.92 (-1.65)	1.89 (0.42)	No
	Haemophagocytic Lymphohistiocytosis	6 / 944	379 / 62574	1.14 (0.52-2.47)	1.05 (0.01)	0.06 (-1.52)	1.04 (0.41)	No
	Hydronephrosis	3 / 944	98 / 62574	2.36 (0.81-6.86)	2.03 (1.52)	0.81 (-1.70)	1.75 (0.41)	No
	Gastrointestinal Disorder	4 / 944	279 / 62574	1.07 (0.42-2.71)	0.95 (0.01)	-0.07 (-1.93)	0.96 (0.32)	No
	Cough	7 / 944	568 / 62574	0.87 (0.42-1.80)	0.82 (0.29)	-0.27 (-1.67)	0.83 (0.37)	No
	Hepatic Enzyme Increased	3 / 944	145 / 62574	1.59 (0.55-4.61)	1.37 (0.30)	0.38 (-1.92)	1.30 (0.34)	No
	Inflammation	4 / 944	90 / 62574	3.30 (1.28-8.53)	2.95 (4.93)	1.25 (-1.20)	2.37 (0.57)	No
	Varicella Zoster Virus Infection	3 / 944	37 / 62574	6.20 (2.07-18.58)	5.38 (9.89)	1.68 (-1.42)	3.20 (0.53)	No
	Hypogammaglobulinaemia	10 / 944	563 / 62574	1.24 (0.67-2.29)	1.18 (0.27)	0.22 (-1.06)	1.17 (0.55)	No
	Lung Neoplasm	3 / 944	20 / 62574	11.34 (3.65-35.31)	9.94 (20.99)	2.06 (-1.38)	4.16 (0.56)	No
	Swelling	3 / 944	135 / 62574	1.71 (0.59-4.96)	1.47 (0.45)	0.46 (-1.87)	1.37 (0.35)	No
	Pruritus	6 / 944	665 / 62574	0.64 (0.30-1.40)	0.60 (1.62)	-0.69 (-2.10)	0.62 (0.27)	No
	Erythema	4 / 944	301 / 62574	0.99 (0.39-2.51)	0.88 (0.06)	-0.16 (-2.00)	0.89 (0.31)	No
	Sinusitis	5 / 944	157 / 62574	2.32 (0.99-5.44)	2.11 (2.84)	0.92 (-1.13)	1.89 (0.57)	No
	General Physical Health Deterioration	5 / 944	464 / 62574	0.78 (0.34-1.82)	0.71 (0.57)	-0.44 (-2.03)	0.74 (0.29)	No
	Myalgia	3 / 944	244 / 62574	0.95 (0.33-2.73)	0.81 (0.12)	-0.25 (-2.30)	0.84 (0.26)	No
	Movement Disorder	7 / 944	33 / 62574	14.94 (6.75-33.05)	14.06 (70.11)	2.78 (-0.12)	6.85 (1.27)	No
	Blood Pressure Decreased	9 / 944	139 / 62574	4.54 (2.35-8.80)	4.29 (21.39)	1.81 (-0.14)	3.52 (1.13)	No
	Haemoglobin Decreased	8 / 944	468 / 62574	1.20 (0.61-2.38)	1.13 (0.12)	0.17 (-1.25)	1.12 (0.49)	No
	Somnolence	3 / 944	249 / 62574	0.93 (0.32-2.67)	0.80 (0.15)	-0.28 (-2.32)	0.82 (0.25)	No
	Temperature Intolerance	5 / 944	105 / 62574	3.47 (1.47-8.19)	3.16 (7.04)	1.36 (-0.92)	2.58 (0.68)	No

Drug	Preferred Term (PT)	Drug Exposure (Events / Total)	Comparator Exposure (Events / Total)	ROR (95% CI)	PRR (Chi-square)	IC (IC025)	EBGM (EB05)	Four-algorithm positive signal
	Hyperhidrosis	3 / 944	155 / 62574	1.49 (0.52-4.31)	1.28 (0.18)	0.30 (-1.96)	1.23 (0.33)	No
	Condition Aggravated	12 / 944	627 / 62574	1.32 (0.75-2.33)	1.27 (0.68)	0.32 (-0.88)	1.25 (0.62)	No
	Muscular Weakness	4 / 944	191 / 62574	1.56 (0.61-3.98)	1.39 (0.43)	0.41 (-1.63)	1.32 (0.41)	No
	Confusional State	9 / 944	397 / 62574	1.59 (0.83-3.03)	1.50 (1.49)	0.54 (-0.90)	1.45 (0.63)	No
	Asthenia	15 / 944	1111 / 62574	0.92 (0.56-1.53)	0.90 (0.19)	-0.15 (-1.14)	0.90 (0.51)	No
	Hepatic Function Abnormal	6 / 944	273 / 62574	1.58 (0.72-3.44)	1.46 (0.84)	0.48 (-1.23)	1.40 (0.52)	No
	Dizziness	4 / 944	573 / 62574	0.52 (0.20-1.31)	0.46 (2.50)	-1.01 (-2.64)	0.50 (0.19)	No
	Lymphadenopathy	3 / 944	462 / 62574	0.50 (0.17-1.43)	0.43 (2.26)	-1.08 (-2.92)	0.47 (0.16)	No
	Nasopharyngitis	6 / 944	213 / 62574	2.02 (0.93-4.43)	1.87 (2.36)	0.79 (-1.04)	1.73 (0.60)	No
	Inappropriate Schedule Of Product Administration	3 / 944	168 / 62574	1.38 (0.48-3.98)	1.18 (0.08)	0.20 (-2.01)	1.15 (0.32)	No
	Progressive Multifocal Leukoencephalopathy	3 / 944	294 / 62574	0.79 (0.27-2.26)	0.68 (0.46)	-0.49 (-2.47)	0.71 (0.23)	No
	Hyperpyrexia	20 / 944	81 / 62574	17.00 (10.43-27.72)	16.37 (231.80)	3.36 (1.26)	10.24 (3.03)	Yes
	Cellulitis	3 / 944	220 / 62574	1.05 (0.36-3.03)	0.90 (0.03)	-0.12 (-2.22)	0.92 (0.27)	No
	Feeding Disorder	3 / 944	38 / 62574	6.04 (2.02-18.07)	5.23 (9.53)	1.66 (-1.42)	3.15 (0.53)	No
	Fungal Infection	8 / 944	142 / 62574	3.98 (1.99-7.97)	3.73 (15.20)	1.64 (-0.33)	3.11 (0.99)	No
	Drug Resistance	5 / 944	150 / 62574	2.43 (1.03-5.70)	2.21 (3.21)	0.97 (-1.10)	1.96 (0.59)	No
	Intestinal Perforation	4 / 944	128 / 62574	2.33 (0.91-5.97)	2.07 (2.15)	0.87 (-1.37)	1.83 (0.50)	No
	Bedridden	3 / 944	14 / 62574	16.04 (4.98-51.63)	14.20 (30.33)	2.22 (-1.38)	4.65 (0.57)	No
	Cytopenia	3 / 944	641 / 62574	0.36 (0.12-1.03)	0.31 (4.63)	-1.52 (-3.28)	0.35 (0.12)	No
	Therapeutic Response Decreased	5 / 944	102 / 62574	3.57 (1.51-8.43)	3.25 (7.43)	1.40 (-0.90)	2.63 (0.69)	No
	Discomfort	3 / 944	70 / 62574	3.30 (1.12-9.66)	2.84 (3.44)	1.14 (-1.56)	2.21 (0.46)	No
	Organ Failure	6 / 944	13 / 62574	32.10 (12.56-82.00)	30.59 (117.60)	3.06 (-0.33)	8.31 (1.16)	No
	Atrial Fibrillation	5 / 944	699 / 62574	0.52 (0.22-1.20)	0.47 (2.93)	-0.99 (-2.47)	0.50 (0.21)	No
	Haematochezia	3 / 944	59 / 62574	3.91 (1.33-11.50)	3.37 (4.76)	1.30 (-1.51)	2.46 (0.48)	No
	Shock	5 / 944	89 / 62574	4.09 (1.72-9.69)	3.72 (9.45)	1.54 (-0.84)	2.90 (0.73)	No
	Bacterial Infection	6 / 944	209 / 62574	2.06 (0.94-4.51)	1.90 (2.51)	0.81 (-1.03)	1.76 (0.60)	No
	Renal Failure	4 / 944	458 / 62574	0.65 (0.26-1.64)	0.58 (1.22)	-0.71 (-2.40)	0.61 (0.23)	No
	Pancytopenia	7 / 944	1090 / 62574	0.45 (0.22-0.93)	0.43 (5.48)	-1.16 (-2.41)	0.45 (0.22)	No
	Viral Infection	3 / 944	105 / 62574	2.20 (0.76-6.40)	1.89 (1.23)	0.73 (-1.73)	1.66 (0.40)	No
	Neuropathy Peripheral	4 / 944	896 / 62574	0.33 (0.13-0.83)	0.30 (6.77)	-1.62 (-3.16)	0.32 (0.13)	No
Mosunetuzumab	Neutrophil Count Decreased	7 / 472	1066 / 63046	0.94 (0.45-1.93)	0.88 (0.12)	-0.18 (-1.59)	0.89 (0.39)	No
	Headache	11 / 472	647 / 63046	2.40 (1.33-4.33)	2.27 (7.77)	1.09 (-0.38)	2.13 (0.90)	No
	Hypoxia	5 / 472	370 / 63046	1.99 (0.85-4.64)	1.80 (1.78)	0.74 (-1.23)	1.67 (0.53)	No
	Covid-19 Pneumonia	34 / 472	682 / 63046	7.19 (5.04-10.25)	6.66 (157.50)	2.57 (1.30)	5.93 (2.84)	Yes
	Sepsis	10 / 472	1369 / 63046	1.02 (0.55-1.89)	0.98 (0.01)	-0.03 (-1.26)	0.98 (0.48)	No
	Cytokine Release Syndrome	110 / 472	4563 / 63046	3.91 (3.15-4.84)	3.22 (177.50)	1.65 (1.10)	3.14 (2.28)	Yes
	Pyrexia	27 / 472	2815 / 63046	1.32 (0.90-1.95)	1.28 (1.73)	0.35 (-0.47)	1.27 (0.79)	No
	Dizziness	3 / 472	574 / 63046	0.81 (0.28-2.33)	0.70 (0.39)	-0.45 (-2.44)	0.73 (0.23)	No
	Muscular Weakness	3 / 472	192 / 63046	2.43 (0.84-7.03)	2.09 (1.68)	0.84 (-1.68)	1.80 (0.41)	No
	Febrile Neutropenia	10 / 472	3101 / 63046	0.44 (0.24-0.81)	0.43 (7.88)	-1.17 (-2.22)	0.45 (0.24)	No
	Cytomegalovirus Infection	4 / 472	373 / 63046	1.61 (0.63-4.10)	1.43 (0.52)	0.45 (-1.60)	1.36 (0.41)	No
	Pneumonitis	11 / 472	251 / 63046	6.22 (3.42-11.32)	5.85 (42.59)	2.23 (0.24)	4.70 (1.48)	No
	Pneumonia	23 / 472	2215 / 63046	1.44 (0.95-2.18)	1.39 (2.55)	0.46 (-0.44)	1.37 (0.81)	No
	Fall	3 / 472	502 / 63046	0.93 (0.32-2.67)	0.80 (0.15)	-0.28 (-2.32)	0.82 (0.25)	No
	Covid-19	26 / 472	2569 / 63046	1.40 (0.94-2.07)	1.35 (2.46)	0.42 (-0.42)	1.34 (0.82)	No
	Infusion Related Reaction	13 / 472	559 / 63046	3.28 (1.90-5.67)	3.11 (18.31)	1.51 (-0.00)	2.84 (1.18)	No
	Liver Function Test Increased	4 / 472	60 / 63046	10.00 (3.82-26.17)	8.90 (26.34)	2.21 (-0.95)	4.61 (0.73)	No

Drug	Preferred Term (PT)	Drug Exposure (Events / Total)	Comparator Exposure (Events / Total)	ROR (95% CI)	PRR (Chi-square)	IC (IC025)	EBGM (EB05)	Four-algorithm positive signal
	Anaemia	4 / 472	1857 / 63046	0.32 (0.12-0.80)	0.29 (7.25)	-1.67 (-3.20)	0.31 (0.13)	No
	Aspartate Aminotransferase Increased	11 / 472	423 / 63046	3.69 (2.04-6.67)	3.47 (19.01)	1.63 (-0.06)	3.09 (1.16)	No
	Alanine Aminotransferase Increased	12 / 472	457 / 63046	3.71 (2.10-6.56)	3.51 (21.12)	1.65 (0.02)	3.14 (1.22)	No
	Urinary Tract Infection	7 / 472	486 / 63046	2.07 (1.00-4.29)	1.92 (3.08)	0.85 (-0.88)	1.80 (0.66)	No
	Asthenia	4 / 472	1122 / 63046	0.53 (0.21-1.34)	0.48 (2.34)	-0.98 (-2.62)	0.51 (0.20)	No
	Deep Vein Thrombosis	4 / 472	309 / 63046	1.95 (0.76-4.96)	1.73 (1.22)	0.67 (-1.48)	1.59 (0.46)	No
	Atrial Fibrillation	12 / 472	692 / 63046	2.44 (1.39-4.31)	2.32 (8.92)	1.12 (-0.30)	2.18 (0.95)	No
	Cytomegalovirus Infection Reactivation	5 / 472	357 / 63046	2.06 (0.88-4.81)	1.87 (2.01)	0.79 (-1.20)	1.72 (0.54)	No
	No Adverse Event	12 / 472	737 / 63046	2.29 (1.30-4.04)	2.17 (7.58)	1.04 (-0.36)	2.06 (0.91)	No
	Influenza	3 / 472	254 / 63046	1.84 (0.64-5.30)	1.58 (0.63)	0.54 (-1.83)	1.45 (0.37)	No
	Diarrhoea	10 / 472	1906 / 63046	0.73 (0.39-1.34)	0.70 (1.31)	-0.49 (-1.63)	0.71 (0.37)	No
	Pain	3 / 472	534 / 63046	0.87 (0.30-2.50)	0.75 (0.25)	-0.36 (-2.38)	0.78 (0.24)	No
	Dyspnoea	9 / 472	1044 / 63046	1.22 (0.64-2.32)	1.15 (0.18)	0.19 (-1.15)	1.14 (0.52)	No
	Injection Site Reaction	12 / 472	30 / 63046	56.08 (28.85-109.00)	53.43 (441.30)	3.94 (0.71)	15.39 (2.34)	Yes
	Haemophagocytic Lymphohistiocytosis	5 / 472	380 / 63046	1.94 (0.83-4.52)	1.76 (1.62)	0.71 (-1.25)	1.64 (0.52)	No
	Nervous System Disorder	3 / 472	153 / 63046	3.05 (1.05-8.85)	2.62 (2.95)	1.08 (-1.59)	2.11 (0.45)	No
	Tumour Flare	11 / 472	67 / 63046	23.25 (12.36-43.74)	21.93 (189.00)	3.41 (0.57)	10.65 (2.03)	Yes
	Encephalopathy	3 / 472	242 / 63046	1.93 (0.67-5.57)	1.66 (0.77)	0.59 (-1.80)	1.51 (0.38)	No
	Hypophosphataemia	3 / 472	139 / 63046	3.36 (1.16-9.75)	2.88 (3.62)	1.17 (-1.55)	2.25 (0.46)	No
	Tumour Lysis Syndrome	5 / 472	610 / 63046	1.20 (0.52-2.80)	1.09 (0.04)	0.12 (-1.62)	1.08 (0.39)	No
	Cardiac Failure	3 / 472	483 / 63046	0.96 (0.34-2.77)	0.83 (0.10)	-0.23 (-2.29)	0.85 (0.26)	No
	Hypotension	6 / 472	833 / 63046	1.04 (0.48-2.26)	0.96 (0.01)	-0.05 (-1.61)	0.96 (0.39)	No
	Infection	5 / 472	1307 / 63046	0.56 (0.24-1.29)	0.51 (2.38)	-0.90 (-2.39)	0.54 (0.23)	No
	Neutropenia	17 / 472	2823 / 63046	0.82 (0.51-1.32)	0.80 (0.84)	-0.30 (-1.21)	0.81 (0.48)	No
	Uveitis	3 / 472	25 / 63046	18.42 (6.01-56.52)	16.03 (37.76)	2.31 (-1.38)	4.94 (0.58)	No
	Pulmonary Embolism	3 / 472	311 / 63046	1.50 (0.52-4.32)	1.29 (0.19)	0.30 (-1.96)	1.24 (0.33)	No
	Hyperglycaemia	3 / 472	183 / 63046	2.55 (0.88-7.38)	2.19 (1.91)	0.90 (-1.66)	1.86 (0.42)	No
	Acute Kidney Injury	4 / 472	1097 / 63046	0.54 (0.21-1.38)	0.49 (2.19)	-0.95 (-2.59)	0.52 (0.20)	No
	Malaise	4 / 472	464 / 63046	1.29 (0.51-3.29)	1.15 (0.08)	0.18 (-1.77)	1.13 (0.36)	No
	Chills	6 / 472	625 / 63046	1.39 (0.64-3.03)	1.28 (0.37)	0.33 (-1.34)	1.25 (0.48)	No
	Hepatic Enzyme Increased	3 / 472	145 / 63046	3.22 (1.11-9.34)	2.76 (3.32)	1.13 (-1.57)	2.19 (0.46)	No
	Rash	20 / 472	1289 / 63046	2.17 (1.39-3.39)	2.07 (11.16)	1.00 (-0.08)	2.00 (1.07)	No
	Pleural Effusion	6 / 472	472 / 63046	1.84 (0.85-4.02)	1.70 (1.71)	0.68 (-1.11)	1.60 (0.57)	No
	Platelet Count Decreased	4 / 472	1353 / 63046	0.44 (0.17-1.11)	0.40 (3.78)	-1.23 (-2.83)	0.42 (0.17)	No
	Septic Shock	6 / 472	949 / 63046	0.91 (0.42-1.98)	0.84 (0.17)	-0.23 (-1.74)	0.86 (0.35)	No
	Dry Skin	4 / 472	119 / 63046	5.06 (1.97-13.02)	4.49 (10.52)	1.67 (-1.06)	3.18 (0.65)	No
	Skin Exfoliation	6 / 472	88 / 63046	9.91 (4.45-22.10)	9.11 (40.60)	2.44 (-0.37)	5.42 (1.06)	No
	Thrombocytopenia	5 / 472	1909 / 63046	0.38 (0.16-0.87)	0.35 (6.21)	-1.42 (-2.83)	0.37 (0.16)	No
	Rash Maculo-Papular	3 / 472	213 / 63046	2.19 (0.76-6.33)	1.88 (1.23)	0.73 (-1.73)	1.66 (0.40)	No
	Herpes Zoster	5 / 472	250 / 63046	2.95 (1.26-6.90)	2.67 (5.15)	1.20 (-0.99)	2.30 (0.64)	No
	Fatigue	11 / 472	1893 / 63046	0.81 (0.45-1.45)	0.78 (0.73)	-0.35 (-1.46)	0.79 (0.41)	No
	Immune Effector Cell-Associated Neurotoxicity Syndrome	4 / 472	1906 / 63046	0.31 (0.12-0.78)	0.28 (7.60)	-1.71 (-3.23)	0.31 (0.13)	No
	Interstitial Lung Disease	4 / 472	375 / 63046	1.60 (0.63-4.08)	1.43 (0.50)	0.44 (-1.61)	1.36 (0.41)	No
	Respiratory Failure	3 / 472	648 / 63046	0.72 (0.25-2.06)	0.62 (0.71)	-0.61 (-2.55)	0.66 (0.21)	No
	Fungal Infection	3 / 472	147 / 63046	3.18 (1.10-9.21)	2.73 (3.22)	1.12 (-1.57)	2.17 (0.45)	No
	Pruritus	4 / 472	667 / 63046	0.90 (0.35-2.28)	0.80 (0.20)	-0.29 (-2.08)	0.82 (0.29)	No
	Hepatic Function Abnormal	5 / 472	274 / 63046	2.69 (1.15-6.29)	2.44 (4.18)	1.10 (-1.04)	2.14 (0.62)	No

Drug	Preferred Term (PT)	Drug Exposure (Events / Total)	Comparator Exposure (Events / Total)	ROR (95% CI)	PRR (Chi-square)	IC (IC025)	EBGM (EB05)	Four-algorithm positive signal
	Cellulitis	4 / 472	219 / 63046	2.75 (1.08-7.02)	2.44 (3.35)	1.06 (-1.28)	2.09 (0.53)	No
	C-Reactive Protein Increased	3 / 472	422 / 63046	1.10 (0.38-3.18)	0.95 (0.01)	-0.06 (-2.18)	0.96 (0.28)	No
	Constipation	3 / 472	743 / 63046	0.62 (0.22-1.79)	0.54 (1.19)	-0.79 (-2.69)	0.58 (0.19)	No
	Leukopenia	3 / 472	721 / 63046	0.64 (0.22-1.85)	0.56 (1.07)	-0.75 (-2.66)	0.59 (0.20)	No
	Vomiting	5 / 472	947 / 63046	0.77 (0.33-1.79)	0.70 (0.62)	-0.46 (-2.05)	0.73 (0.29)	No
	Nausea	5 / 472	1484 / 63046	0.49 (0.21-1.13)	0.45 (3.43)	-1.07 (-2.54)	0.48 (0.20)	No
	Hypogammaglobulinaemia	6 / 472	567 / 63046	1.53 (0.70-3.34)	1.41 (0.72)	0.45 (-1.26)	1.37 (0.51)	No
	Erythema	3 / 472	302 / 63046	1.55 (0.54-4.45)	1.33 (0.24)	0.34 (-1.94)	1.26 (0.34)	No
	Liver Disorder	5 / 472	134 / 63046	5.50 (2.33-12.97)	4.98 (15.38)	1.84 (-0.74)	3.59 (0.80)	No
	Skin Disorder	3 / 472	102 / 63046	4.58 (1.57-13.34)	3.93 (6.37)	1.45 (-1.47)	2.73 (0.50)	No

Note: Positive signal indicates simultaneous positivity across the prespecified ROR, PRR, BCPNN (IC025), and MGPS (EB05) criteria. PTs with at least three exposed reports are shown. CI, confidence interval; EBGM, empirical Bayes geometric mean; IC, information component; PRR, proportional reporting ratio; PT, preferred term; ROR, reporting odds ratio.

Supplementary Table S7. Time-to-onset distributions, phenotype prevalence, and clinical consequences of prespecified safety phenotypes.

A. Time-to-onset distribution by drug and safety endpoint

Drug	Safety Endpoint	Total Cases / Valid TTO Cases	Median TTO Days (IQR)	≤ 7 days (%)	8–30 days (%)	31–90 days (%)	> 90 days (%)
**Epcoritamab**	CMV	120 / 56	20.5 (8.0-40.2)	13 (23.2%)	23 (41.1%)	13 (23.2%)	7 (12.5%)
	COVID-19	69 / 25	24.0 (12.0-76.0)	4 (16.0%)	13 (52.0%)	2 (8.0%)	6 (24.0%)
	CRS	459 / 201	8.0 (2.0-15.0)	88 (43.8%)	107 (53.2%)	3 (1.5%)	3 (1.5%)
	ICANS	119 / 53	12.0 (5.0-15.0)	19 (35.8%)	32 (60.4%)	1 (1.9%)	1 (1.9%)
	Infection composite	374 / 195	21.0 (7.5-64.5)	49 (25.1%)	72 (36.9%)	36 (18.5%)	38 (19.5%)
	Opportunistic infection	153 / 75	30.0 (12.0-72.5)	14 (18.7%)	26 (34.7%)	19 (25.3%)	16 (21.3%)
	PJP	10 / 5	34.0 (1.0-56.0)	2 (40.0%)	0 (0.0%)	3 (60.0%)	0 (0.0%)
	PML	7 / 5	529.0 (360.0-687.0)	0 (0.0%)	1 (20.0%)	0 (0.0%)	4 (80.0%)
	Pneumonia/LRI	115 / 59	26.0 (12.0-118.5)	12 (20.3%)	20 (33.9%)	10 (16.9%)	17 (28.8%)
	Serious infection proxy	296 / 156	20.5 (7.0-69.2)	41 (26.3%)	57 (36.5%)	26 (16.7%)	32 (20.5%)
**Glofitamab**	CMV	17 / 5	28.0 (19.0-36.0)	1 (20.0%)	2 (40.0%)	2 (40.0%)	0 (0.0%)
	COVID-19	44 / 19	102.0 (7.5-207.0)	5 (26.3%)	2 (10.5%)	2 (10.5%)	10 (52.6%)
	CRS	253 / 98	1.0 (0.0-2.0)	83 (84.7%)	10 (10.2%)	2 (2.0%)	3 (3.1%)
	ICANS	47 / 9	1.0 (0.0-2.0)	8 (88.9%)	1 (11.1%)	0 (0.0%)	0 (0.0%)
	Infection composite	156 / 54	57.5 (13.5-173.2)	12 (22.2%)	10 (18.5%)	11 (20.4%)	21 (38.9%)
	Opportunistic infection	29 / 12	56.0 (14.8-158.8)	3 (25.0%)	2 (16.7%)	2 (16.7%)	5 (41.7%)
	PJP	2 / 1	2.0 (2.0-2.0)	1 (100.0%)	0 (0.0%)	0 (0.0%)	0 (0.0%)
	PML	3 / 2	253.0 (217.5-288.5)	0 (0.0%)	0 (0.0%)	0 (0.0%)	2 (100.0%)
	Pneumonia/LRI	58 / 23	71.0 (26.5-143.0)	2 (8.7%)	4 (17.4%)	8 (34.8%)	9 (39.1%)
	Serious infection proxy	113 / 38	63.0 (19.0-166.0)	6 (15.8%)	7 (18.4%)	11 (28.9%)	14 (36.8%)
**Mosunetuzumab**	CMV	9 / 7	30.0 (5.0-93.5)	2 (28.6%)	2 (28.6%)	1 (14.3%)	2 (28.6%)
	COVID-19	60 / 51	120.0 (39.5-185.0)	6 (11.8%)	5 (9.8%)	11 (21.6%)	29 (56.9%)
	CRS	110 / 59	1.0 (1.0-10.0)	39 (66.1%)	15 (25.4%)	3 (5.1%)	2 (3.4%)
	ICANS	4 / 2	17.0 (10.0-24.0)	1 (50.0%)	0 (0.0%)	1 (50.0%)	0 (0.0%)
	Infection composite	138 / 107	85.0 (26.5-178.5)	15 (14.0%)	14 (13.1%)	28 (26.2%)	50 (46.7%)
	Opportunistic infection	20 / 15	63.0 (26.0-131.0)	2 (13.3%)	3 (20.0%)	5 (33.3%)	5 (33.3%)
	PJP	2 / 2	48.0 (40.5-55.5)	0 (0.0%)	0 (0.0%)	2 (100.0%)	0 (0.0%)
	Pneumonia/LRI	59 / 47	109.0 (57.5-185.0)	3 (6.4%)	5 (10.6%)	12 (25.5%)	27 (57.4%)
	Serious infection proxy	96 / 72	87.0 (29.2-175.8)	8 (11.1%)	11 (15.3%)	18 (25.0%)	35 (48.6%)

B. Prespecified phenotype counts

Phenotype	Cases
**CRS + ICANS**	78
**CRS + ICANS + infection**	28
**CRS + infection**	163
**CRS only**	553
**ICANS + infection**	12
**ICANS only**	52
**Infection only**	465
**HLH + infection**	6
**Opportunistic infection**	202

C. Drug-specific phenotype prevalence

Drug	Phenotype	Cases	All exposed reports	Prevalence (%)
**Epcoritamab**	CRS + ICANS	49	1463	3%
**Epcoritamab**	CRS + ICANS + infection	22	1463	2%
**Epcoritamab**	CRS + infection	108	1463	7%
**Epcoritamab**	CRS only	280	1463	19%
**Epcoritamab**	HLH + infection	5	1463	<1%
**Epcoritamab**	ICANS + infection	12	1463	<1%
**Epcoritamab**	ICANS only	36	1463	2%
**Epcoritamab**	Infection only	232	1463	16%
**Epcoritamab**	Opportunistic infection	153	1463	10%
**Glofitamab**	CRS + ICANS	29	944	3%
**Glofitamab**	CRS + ICANS + infection	5	944	<1%
**Glofitamab**	CRS + infection	34	944	4%
**Glofitamab**	CRS only	185	944	20%
**Glofitamab**	HLH + infection	0	944	0%
**Glofitamab**	ICANS + infection	0	944	0%
**Glofitamab**	ICANS only	13	944	1%
**Glofitamab**	Infection only	117	944	12%
**Glofitamab**	Opportunistic infection	29	944	3%
**Mosunetuzumab**	CRS + ICANS	0	472	0%
**Mosunetuzumab**	CRS + ICANS + infection	1	472	<1%
**Mosunetuzumab**	CRS + infection	21	472	4%
**Mosunetuzumab**	CRS only	88	472	19%
**Mosunetuzumab**	HLH + infection	1	472	<1%
**Mosunetuzumab**	ICANS + infection	0	472	0%
**Mosunetuzumab**	ICANS only	3	472	<1%
**Mosunetuzumab**	Infection only	116	472	25%

Drug	Phenotype	Cases	All exposed reports	Prevalence (%)
**Mosunetuzumab**	Opportunistic infection	20	472	4%

D. Clinical consequences among phenotype-positive reports

Phenotype	Cases	Death	Severe outcome	Hospitalization
**CRS + ICANS + infection**	28	61%	79%	43%
**CRS + infection**	163	40%	64%	39%
**ICANS + infection**	12	58%	75%	58%
**CRS + ICANS**	78	37%	73%	45%
**CRS only**	553	20%	56%	39%
**ICANS only**	52	37%	60%	23%
**Infection only**	465	37%	81%	59%
**HLH + infection**	6	33%	67%	50%
**Opportunistic infection**	202	27%	60%	43%

Note: The seven CRS/ICANS/infection core phenotypes are mutually exclusive. HLH + infection and opportunistic infection are non-mutually exclusive contextual phenotypes. Clinical outcomes are non-mutually exclusive.

Abbreviations: CMV, cytomegalovirus; COVID-19, coronavirus disease 2019; CRS, cytokine release syndrome; HLH, hemophagocytic lymphohistiocytosis; ICANS, immune effector cell-associated neurotoxicity syndrome; IQR, interquartile range; LRI, lower respiratory infection; PJP, Pneumocystis jirovecii pneumonia; PML, progressive multifocal leukoencephalopathy; TTO, time to onset.

Supplementary Table S8. Cross-database descriptive comparison of safety endpoints by CD20×CD3 bispecific antibody exposure

Drug	Safety endpoint	FAERS exposure (events / total, %)	JADER exposure (events / total, %)
**Epcoritamab**	CRS	459 / 1463 (31.4%)	801 / 1240 (64.6%)
	ICANS	119 / 1463 (8.1%)	139 / 1240 (11.2%)
	Infection composite	374 / 1463 (25.6%)	275 / 1240 (22.2%)
	Pneumonia/LRI	115 / 1463 (7.9%)	113 / 1240 (9.1%)
	COVID-19	69 / 1463 (4.7%)	43 / 1240 (3.5%)
	CMV	120 / 1463 (8.2%)	88 / 1240 (7.1%)
	Opportunistic infection	153 / 1463 (10.5%)	124 / 1240 (10.0%)
**Glofitamab**	CRS	253 / 944 (26.8%)	0 / 0 (NA)
	ICANS	47 / 944 (5.0%)	0 / 0 (NA)
	Infection composite	156 / 944 (16.5%)	0 / 0 (NA)
	Pneumonia/LRI	58 / 944 (6.1%)	0 / 0 (NA)
	COVID-19	44 / 944 (4.7%)	0 / 0 (NA)
	CMV	17 / 944 (1.8%)	0 / 0 (NA)
	Opportunistic infection	29 / 944 (3.1%)	0 / 0 (NA)
**Mosunetuzumab**	CRS	110 / 472 (23.3%)	63 / 101 (62.4%)
	ICANS	4 / 472 (0.8%)	5 / 101 (5.0%)
	Infection composite	138 / 472 (29.2%)	13 / 101 (12.9%)
	Pneumonia/LRI	59 / 472 (12.5%)	9 / 101 (8.9%)
	COVID-19	60 / 472 (12.7%)	8 / 101 (7.9%)
	CMV	9 / 472 (1.9%)	1 / 101 (1.0%)
	Opportunistic infection	20 / 472 (4.2%)	4 / 101 (4.0%)

Values are shown as events / total (%). Percentages were calculated within the corresponding drug-specific exposure denominator in each database.
Abbreviations: CD20×CD3, CD20/CD3 bispecific antibody; CMV, cytomegalovirus; COVID-19, coronavirus disease 2019; CRS, cytokine release syndrome; FAERS, FDA Adverse Event Reporting System; ICANS, immune effector cell-associated neurotoxicity syndrome; JADER, Japanese Adverse Drug Event Report; LRI, lower respiratory infection; NA, not applicable.

Supplementary Table S9.

Context / interpretation	Epcoritamab	Glofitamab	Mosunetuzumab
**FDA-label and clinical context**	CRS and ICANS/neurologic toxicity warnings; serious and opportunistic infection concerns; step-up dosing.	CRS and neurologic toxicity warnings; anti-CD20 pretreatment and fixed-duration regimen context; serious infection concerns.	CRS and neurologic toxicity warnings; infection concerns; FL-heavy treatment context.
**Primary FAERS reporting pattern**	Shared CRS pattern; higher ICANS, CMV, and narrow opportunistic-infection reporting.	Shared CRS pattern; ICANS CI crossed 1 in the primary model but was higher in extended treatment-context models; infection-composite reporting odds were lower in the primary model.	Shared CRS pattern; higher pneumonia/LRI and COVID-19 reporting in the primary analysis; ICANS sparse.
**Descriptive JADER context**	Broad CRS, ICANS, and infection categories were present in 1,240 suspected-drug reports.	No reports were detected using the prespecified JADER dictionary; this is not negative safety evidence.	CRS and selected infection categories were present, but only 101 suspected-drug reports were available.
**Key interpretive qualification**	Label salience may stimulate reporting; estimates are reporting odds rather than incidence.	ICANS was covariate-sensitive, and pretreatment/regimen context complicates interpretation.	Infection-specific estimates attenuated or became unstable in US-only and common-calendar analyses.

Supplementary Table S10.

Endpoint	Events, n (%)	Crude ROR (95% CI)	Primary aROR (95% CI)	Extended aROR (95% CI)	Excl. corticosteroid aROR (95% CI)	Excl. anti-CD20 aROR (95% CI)	Excl. both aROR (95% CI)
**Epcoritamab (N = 1,463)**							
CRS	459 (31.4%)	6.283 (5.602-7.046)	4.698 (4.151-5.318)	8.866 (7.669-10.251)	10.102 (8.763-11.647)	10.423 (9.062-11.989)	11.033 (9.609-12.667)
ICANS	119 (8.1%)	2.991 (2.466-3.628)	2.285 (1.865-2.798)	9.874 (7.662-12.724)	11.310 (8.813-14.516)	11.365 (8.884-14.540)	11.977 (9.380-15.293)
Broadly defined neurotoxicity	185 (12.6%)	1.725 (1.475-2.018)	1.704 (1.448-2.005)	2.620 (2.202-3.117)	2.726 (2.294-3.240)	2.915 (2.457-3.458)	2.963 (2.499-3.514)
Infection composite	374 (25.6%)	1.440 (1.279-1.623)	1.354 (1.197-1.531)	2.232 (1.958-2.544)	2.386 (2.095-2.718)	1.944 (1.709-2.211)	2.037 (1.793-2.315)
Serious infection proxy	296 (20.2%)	1.699 (1.493-1.934)	1.524 (1.333-1.743)	2.384 (2.064-2.753)	2.621 (2.272-3.022)	2.198 (1.909-2.532)	2.341 (2.035-2.693)
Pneumonia/lower respiratory infection	115 (7.9%)	1.289 (1.063-1.563)	1.202 (0.985-1.466)	1.499 (1.216-1.849)	1.633 (1.326-2.010)	1.364 (1.110-1.676)	1.453 (1.184-1.783)
COVID-19	69 (4.7%)	0.855 (0.670-1.090)	0.973 (0.757-1.252)	1.654 (1.272-2.150)	1.590 (1.224-2.067)	1.106 (0.856-1.430)	1.091 (0.844-1.410)
CMV	120 (8.2%)	5.806 (4.767-7.071)	4.536 (3.662-5.617)	10.903 (8.444-14.078)	11.027 (8.556-14.212)	9.425 (7.376-12.043)	9.539 (7.471-12.180)
Narrow opportunistic infection	153 (10.5%)	2.356 (1.985-2.796)	2.201 (1.840-2.634)	4.391 (3.603-5.352)	4.834 (3.974-5.881)	3.984 (3.284-4.833)	4.232 (3.493-5.126)
**Glofitamab (N = 944)**							
CRS	253 (26.8%)	4.826 (4.166-5.591)	3.433 (2.944-4.004)	8.155 (6.908-9.626)	7.114 (6.037-8.383)	8.074 (6.850-9.517)	7.327 (6.225-8.625)
ICANS	47 (5.0%)	1.725 (1.284-2.318)	1.259 (0.931-1.703)	5.713 (4.113-7.936)	5.193 (3.748-7.195)	5.549 (3.996-7.706)	5.252 (3.789-7.279)
Broadly defined neurotoxicity	71 (7.5%)	0.958 (0.751-1.221)	0.896 (0.700-1.146)	1.499 (1.166-1.926)	1.461 (1.137-1.877)	1.502 (1.169-1.931)	1.478 (1.150-1.899)
Infection composite	156 (16.5%)	0.822 (0.691-0.977)	0.780 (0.655-0.929)	1.121 (0.937-1.340)	1.086 (0.908-1.297)	1.107 (0.926-1.324)	1.059 (0.887-1.265)
Serious infection proxy	113 (12.0%)	0.899 (0.738-1.095)	0.810 (0.663-0.989)	1.178 (0.960-1.444)	1.122 (0.916-1.375)	1.173 (0.956-1.438)	1.104 (0.901-1.353)
Pneumonia/lower respiratory infection	58 (6.1%)	0.987 (0.756-1.288)	0.970 (0.740-1.271)	1.170 (0.890-1.540)	1.135 (0.863-1.492)	1.162 (0.884-1.529)	1.113 (0.847-1.464)
COVID-19	44 (4.7%)	0.848 (0.627-1.149)	1.052 (0.772-1.434)	1.212 (0.886-1.658)	1.213 (0.887-1.661)	1.144 (0.837-1.564)	1.152 (0.843-1.575)
CMV	17 (1.8%)	1.109 (0.688-1.786)	0.774 (0.475-1.261)	1.158 (0.706-1.900)	1.135 (0.692-1.862)	1.156 (0.705-1.897)	1.128 (0.688-1.850)
Narrow opportunistic infection	29 (3.1%)	0.626 (0.433-0.905)	0.529 (0.364-0.768)	0.875 (0.600-1.278)	0.831 (0.569-1.212)	0.874 (0.599-1.275)	0.817 (0.560-1.192)
**Mosunetuzumab (N = 472)**							
CRS	110 (23.3%)	3.909 (3.153-4.847)	6.229 (4.969-7.808)	6.194 (4.882-7.857)	7.933 (6.281-10.020)	7.806 (6.176-9.865)	9.111 (7.229-11.482)
ICANS	4 (0.8%)	0.308 (0.122-0.781)	0.480 (0.178-1.294)	0.836 (0.308-2.268)	1.102 (0.407-2.982)	1.040 (0.384-2.816)	1.215 (0.449-3.285)
Broadly defined neurotoxicity	16 (3.4%)	0.421 (0.258-0.689)	0.491 (0.297-0.813)	0.532 (0.321-0.881)	0.587 (0.354-0.971)	0.597 (0.360-0.989)	0.634 (0.383-1.049)
Infection composite	138 (29.2%)	1.727 (1.415-2.108)	1.537 (1.254-1.885)	1.954 (1.589-2.403)	2.218 (1.805-2.725)	1.766 (1.437-2.170)	1.983 (1.615-2.435)
Serious infection proxy	96 (20.3%)	1.698 (1.356-2.126)	1.654 (1.314-2.081)	1.940 (1.534-2.452)	2.323 (1.841-2.931)	1.845 (1.461-2.330)	2.160 (1.713-2.723)
Pneumonia/lower respiratory infection	59 (12.5%)	2.169 (1.650-2.852)	1.745 (1.318-2.309)	1.920 (1.443-2.554)	2.202 (1.658-2.925)	1.773 (1.335-2.355)	2.004 (1.511-2.658)
COVID-19	60 (12.7%)	2.551 (1.943-3.348)	1.663 (1.254-2.206)	2.798 (2.096-3.735)	2.625 (1.970-3.499)	1.875 (1.409-2.494)	1.825 (1.373-2.425)
CMV	9 (1.9%)	1.204 (0.632-2.296)	1.291 (0.659-2.528)	1.676 (0.849-3.310)	1.774 (0.900-3.496)	1.656 (0.841-3.260)	1.732 (0.881-3.407)
Narrow opportunistic infection	20 (4.2%)	0.884 (0.567-1.379)	0.925 (0.587-1.457)	1.183 (0.747-1.873)	1.446 (0.914-2.286)	1.136 (0.718-1.796)	1.350 (0.855-2.133)

ROR, reporting odds ratio; aROR, adjusted reporting odds ratio. Model 0: crude; Model 1: primary minimally adjusted; Model 2: extended treatment-context adjusted; Model 3: extended excluding corticosteroid; Model 4: extended excluding anti-CD20; Model 5: extended excluding corticosteroid and anti-CD20. Crude estimates used the Haldane-Anscombe 0.5 correction. Adjusted models used a tiny ridge term (lambda=1e-6) for numerical stability; all adjusted models converged.

Supplementary Table S11.

Endpoint	Events, n (%)	Crude ROR (95% CI)	Primary aROR (95% CI)	Extended aROR (95% CI)
**A: LBCL/DLBCL/HGBCL restricted (analysis cohort N = 25,936)**				
**Epcoritamab (N = 1,255)**				
CRS	421 (33.5%)	4.349 (3.841-4.923)<br>Preserved	4.570 (4.000-5.221)<br>Preserved	7.835 (6.623-9.268)<br>Preserved
ICANS	108 (8.6%)	1.981 (1.612-2.433)<br>Preserved	2.252 (1.815-2.793)<br>Preserved	9.311 (6.882-12.596)<br>Preserved
Broadly defined neurotoxicity	165 (13.1%)	1.579 (1.333-1.870)<br>Preserved	1.888 (1.581-2.253)<br>Preserved	3.400 (2.766-4.179)<br>Preserved
Infection composite	314 (25.0%)	1.359 (1.192-1.550)<br>Preserved	1.274 (1.112-1.460)<br>Preserved	2.047 (1.754-2.388)<br>Preserved
Serious infection proxy	249 (19.8%)	1.570 (1.360-1.812)<br>Preserved	1.428 (1.230-1.657)<br>Preserved	2.380 (2.005-2.824)<br>Preserved
Pneumonia/lower respiratory infection	97 (7.7%)	1.278 (1.032-1.581)<br>Attenuated	1.084 (0.870-1.353)<br>Preserved	1.333 (1.039-1.710)<br>Attenuated
COVID-19	56 (4.5%)	0.942 (0.717-1.238)<br>Preserved	0.970 (0.731-1.287)<br>Preserved	1.206 (0.883-1.646)<br>Preserved
CMV	104 (8.3%)	4.892 (3.923-6.102)<br>Preserved	4.455 (3.498-5.673)<br>Preserved	12.065 (8.743-16.649)<br>Preserved
Narrow opportunistic infection	129 (10.3%)	2.241 (1.851-2.712)<br>Preserved	2.230 (1.825-2.726)<br>Preserved	5.467 (4.277-6.987)<br>Preserved
**Glofitamab (N = 779)**				
CRS	191 (24.5%)	2.600 (2.198-3.075)<br>Preserved	2.637 (2.218-3.136)<br>Preserved	5.654 (4.663-6.856)<br>Preserved
ICANS	30 (3.9%)	0.811 (0.562-1.170)<br>Preserved	0.872 (0.600-1.266)<br>Preserved	3.121 (2.082-4.679)<br>Attenuated
Broadly defined neurotoxicity	49 (6.3%)	0.680 (0.508-0.909)<br>Attenuated	0.758 (0.564-1.019)<br>Preserved	1.390 (1.024-1.888)<br>Unstable
Infection composite	122 (15.7%)	0.740 (0.609-0.900)<br>Preserved	0.719 (0.590-0.876)<br>Preserved	1.046 (0.853-1.281)<br>Unstable
Serious infection proxy	88 (11.3%)	0.785 (0.627-0.982)<br>Preserved	0.742 (0.592-0.931)<br>Preserved	1.088 (0.862-1.374)<br>Unstable
Pneumonia/lower respiratory infection	45 (5.8%)	0.926 (0.683-1.255)<br>Preserved	0.878 (0.645-1.194)<br>Preserved	1.105 (0.806-1.515)<br>Preserved
COVID-19	33 (4.2%)	0.897 (0.632-1.274)<br>Preserved	0.948 (0.664-1.355)<br>Preserved	1.078 (0.749-1.553)<br>Preserved
CMV	13 (1.7%)	0.802 (0.465-1.383)<br>Preserved	0.654 (0.374-1.143)<br>Preserved	0.866 (0.490-1.533)<br>Preserved
Narrow opportunistic infection	21 (2.7%)	0.515 (0.334-0.793)<br>Preserved	0.456 (0.294-0.708)<br>Preserved	0.753 (0.482-1.177)<br>Attenuated

Endpoint	Events, n (%)	Crude ROR (95% CI)	Primary aROR (95% CI)	Extended aROR (95% CI)
**Mosunetuzumab (N = 38)**				
CRS	9 (23.7%)	2.476 (1.191-5.150) Attenuated	3.030 (1.403-6.542) Attenuated	2.470 (1.113-5.479) Attenuated
ICANS	0 (0.0%)	0.260 (0.016-4.233) Unstable	Not estimable	Not estimable
Broadly defined neurotoxicity	1 (2.6%)	0.405 (0.079-2.072) Unstable	0.272 (0.037-2.005) Unstable	0.353 (0.048-2.591) Unstable
Infection composite	14 (36.8%)	2.374 (1.239-4.548) Preserved	2.430 (1.244-4.746) Preserved	3.206 (1.630-6.307) Preserved
Serious infection proxy	8 (21.1%)	1.723 (0.805-3.687) Attenuated	1.705 (0.776-3.749) Attenuated	2.227 (1.006-4.929) Preserved
Pneumonia/lower respiratory infection	6 (15.8%)	3.004 (1.292-6.985) Preserved	2.618 (1.081-6.339) Preserved	2.860 (1.170-6.994) Preserved
COVID-19	7 (18.4%)	4.798 (2.159-10.661) Preserved	3.931 (1.688-9.155) Preserved	4.718 (2.007-11.092) Preserved
CMV	2 (5.3%)	3.148 (0.872-11.369) Unstable	3.562 (0.835-15.191) Unstable	3.984 (0.916-17.324) Unstable
Narrow opportunistic infection	3 (7.9%)	1.821 (0.606-5.466) Preserved	2.222 (0.678-7.285) Preserved	3.351 (1.013-11.082) Unstable
**B: Non-FL (analysis cohort N = 55,262)**				
**Epcoritamab (N = 1,363)**				
CRS	438 (32.1%)	6.260 (5.561-7.046) Preserved	4.629 (4.073-5.260) Preserved	9.693 (8.325-11.287) Preserved
ICANS	114 (8.4%)	2.813 (2.309-3.427) Preserved	2.231 (1.813-2.744) Preserved	9.304 (7.175-12.063) Preserved
Broadly defined neurotoxicity	175 (12.8%)	1.644 (1.399-1.932) Preserved	1.686 (1.426-1.994) Preserved	2.619 (2.188-3.135) Preserved
Infection composite	344 (25.2%)	1.500 (1.325-1.698) Preserved	1.350 (1.188-1.535) Preserved	2.119 (1.847-2.430) Preserved
Serious infection proxy	272 (20.0%)	1.701 (1.486-1.947) Preserved	1.489 (1.294-1.713) Preserved	2.320 (1.995-2.699) Preserved
Pneumonia/lower respiratory infection	107 (7.9%)	1.404 (1.149-1.716) Attenuated	1.216 (0.988-1.495) Preserved	1.469 (1.178-1.832) Attenuated
COVID-19	65 (4.8%)	1.130 (0.878-1.453) Preserved	1.143 (0.882-1.483) Preserved	1.657 (1.260-2.178) Unstable
CMV	112 (8.2%)	5.978 (4.869-7.339) Preserved	4.611 (3.685-5.770) Preserved	10.725 (8.174-14.073) Preserved
Narrow opportunistic infection	141 (10.3%)	2.316 (1.937-2.769) Preserved	2.192 (1.817-2.644) Preserved	4.214 (3.422-5.189) Preserved

Endpoint	Events, n (%)	Crude ROR (95% CI)	Primary aROR (95% CI)	Extended aROR (95% CI)
**Glofitamab (N = 925)**				
CRS	247 (26.7%)	4.611 (3.972-5.352)<br>Preserved	3.392 (2.903-3.964)<br>Preserved	8.397 (7.087-9.949)<br>Preserved
ICANS	45 (4.9%)	1.535 (1.135-2.076)<br>Attenuated	1.214 (0.892-1.652)<br>Preserved	5.081 (3.637-7.098)<br>Attenuated
Broadly defined neurotoxicity	69 (7.5%)	0.888 (0.694-1.137)<br>Preserved	0.881 (0.686-1.131)<br>Preserved	1.455 (1.128-1.877)<br>Unstable
Infection composite	152 (16.4%)	0.863 (0.725-1.029)<br>Attenuated	0.782 (0.655-0.934)<br>Preserved	1.115 (0.930-1.336)<br>Unstable
Serious infection proxy	110 (11.9%)	0.907 (0.743-1.109)<br>Attenuated	0.803 (0.655-0.983)<br>Preserved	1.178 (0.958-1.449)<br>Unstable
Pneumonia/lower respiratory infection	58 (6.3%)	1.098 (0.840-1.434)<br>Preserved	1.000 (0.763-1.311)<br>Preserved	1.211 (0.920-1.595)<br>Preserved
COVID-19	43 (4.6%)	1.102 (0.811-1.499)<br>Preserved	1.122 (0.821-1.534)<br>Preserved	1.140 (0.829-1.566)<br>Preserved
CMV	17 (1.8%)	1.152 (0.715-1.858)<br>Preserved	0.789 (0.484-1.286)<br>Preserved	1.136 (0.691-1.867)<br>Preserved
Narrow opportunistic infection	28 (3.0%)	0.612 (0.421-0.891)<br>Preserved	0.521 (0.356-0.763)<br>Preserved	0.852 (0.580-1.252)<br>Attenuated
**Mosunetuzumab (N = 236)**				
CRS	48 (20.3%)	3.128 (2.278-4.295)<br>Attenuated	4.793 (3.447-6.663)<br>Preserved	4.984 (3.538-7.019)<br>Preserved
ICANS	1 (0.4%)	0.187 (0.038-0.931)<br>Unstable	0.170 (0.024-1.219)<br>Unstable	0.290 (0.040-2.083)<br>Unstable
Broadly defined neurotoxicity	8 (3.4%)	0.407 (0.205-0.808)<br>Preserved	0.370 (0.182-0.751)<br>Preserved	0.404 (0.199-0.823)<br>Preserved
Infection composite	85 (36.0%)	2.490 (1.908-3.248)<br>Preserved	2.425 (1.849-3.179)<br>Preserved	2.662 (2.020-3.507)<br>Preserved
Serious infection proxy	56 (23.7%)	2.108 (1.562-2.846)<br>Preserved	2.113 (1.556-2.868)<br>Preserved	2.232 (1.634-3.048)<br>Preserved
Pneumonia/lower respiratory infection	35 (14.8%)	2.884 (2.015-4.128)<br>Preserved	2.648 (1.834-3.824)<br>Preserved	2.553 (1.754-3.717)<br>Preserved
COVID-19	40 (16.9%)	4.665 (3.318-6.559)<br>Preserved	3.954 (2.782-5.621)<br>Preserved	5.099 (3.549-7.327)<br>Preserved
CMV	6 (2.5%)	1.687 (0.772-3.689)<br>Preserved	2.270 (0.994-5.183)<br>Preserved	2.668 (1.156-6.161)<br>Attenuated
Narrow opportunistic infection	12 (5.1%)	1.081 (0.611-1.914)<br>Preserved	1.135 (0.631-2.044)<br>Preserved	1.280 (0.707-2.319)<br>Preserved

Endpoint	Events, n (%)	Crude ROR (95% CI)	Primary aROR (95% CI)	Extended aROR (95% CI)
**C: US only (analysis cohort N = 21,389)**				
**Epcoritamab (N = 175)**				
CRS	31 (17.7%)	3.184 (2.157-4.699)<br>Preserved	1.591 (1.055-2.398)<br>Attenuated	6.916 (4.498-10.633)<br>Preserved
ICANS	6 (3.4%)	0.997 (0.454-2.190)<br>Unstable	0.469 (0.206-1.072)<br>Unstable	3.866 (1.598-9.350)<br>Preserved
Broadly defined neurotoxicity	17 (9.7%)	0.983 (0.599-1.614)<br>Unstable	0.644 (0.387-1.073)<br>Unstable	1.352 (0.807-2.266)<br>Attenuated
Infection composite	24 (13.7%)	1.026 (0.669-1.576)<br>Attenuated	0.959 (0.619-1.485)<br>Unstable	1.540 (0.986-2.404)<br>Attenuated
Serious infection proxy	18 (10.3%)	1.208 (0.744-1.960)<br>Attenuated	1.051 (0.639-1.727)<br>Attenuated	1.858 (1.120-3.083)<br>Preserved
Pneumonia/lower respiratory infection	9 (5.1%)	1.346 (0.698-2.598)<br>Preserved	1.306 (0.661-2.581)<br>Preserved	1.849 (0.926-3.692)<br>Preserved
COVID-19	5 (2.9%)	0.981 (0.418-2.301)<br>Preserved	1.231 (0.500-3.035)<br>Preserved	1.406 (0.565-3.497)<br>Preserved
CMV	5 (2.9%)	5.293 (2.225-12.593)<br>Preserved	3.830 (1.507-9.735)<br>Preserved	10.350 (3.906-27.421)<br>Preserved
Narrow opportunistic infection	11 (6.3%)	2.286 (1.251-4.178)<br>Preserved	1.842 (0.980-3.464)<br>Attenuated	5.122 (2.664-9.848)<br>Preserved
**Glofitamab (N = 122)**				
CRS	58 (47.5%)	13.569 (9.484-19.414)<br>Preserved	16.015 (10.687-24.000)<br>Preserved	64.833 (42.936-97.898)<br>Preserved
ICANS	16 (13.1%)	4.094 (2.425-6.910)<br>Attenuated	3.401 (1.949-5.934)<br>Attenuated	29.158 (15.960-53.271)<br>Attenuated
Broadly defined neurotoxicity	21 (17.2%)	1.896 (1.188-3.026)<br>Unstable	1.711 (1.054-2.776)<br>Unstable	3.436 (2.108-5.600)<br>Unstable
Infection composite	6 (4.9%)	0.353 (0.160-0.778)<br>Preserved	0.340 (0.149-0.775)<br>Preserved	0.413 (0.179-0.952)<br>Preserved
Serious infection proxy	4 (3.3%)	0.388 (0.151-0.997)<br>Preserved	0.337 (0.124-0.919)<br>Preserved	0.441 (0.161-1.212)<br>Attenuated
Pneumonia/lower respiratory infection	2 (1.6%)	0.487 (0.139-1.706)<br>Unstable	0.442 (0.109-1.798)<br>Unstable	0.506 (0.124-2.069)<br>Unstable
COVID-19	2 (1.6%)	0.630 (0.180-2.208)<br>Unstable	0.780 (0.191-3.184)<br>Unstable	0.691 (0.168-2.845)<br>Unstable
CMV	1 (0.8%)	1.969 (0.390-9.943)<br>Unstable	1.168 (0.160-8.553)<br>Unstable	1.760 (0.238-13.037)<br>Unstable
Narrow opportunistic infection	2 (1.6%)	0.670 (0.191-2.351)<br>Unstable	0.530 (0.130-2.170)<br>Unstable	0.847 (0.203-3.543)<br>Unstable

Endpoint	Events, n (%)	Crude ROR (95% CI)	Primary aROR (95% CI)	Extended aROR (95% CI)
**Mosunetuzumab (N = 193)**				
CRS	47 (24.4%)	4.791 (3.438-6.677) Preserved	8.368 (5.837-11.995) Preserved	18.558 (12.715-27.085) Preserved
ICANS	1 (0.5%)	0.201 (0.040-1.004) Unstable	0.213 (0.030-1.534) Unstable	0.707 (0.097-5.137) Unstable
Broadly defined neurotoxicity	7 (3.6%)	0.356 (0.171-0.740) Preserved	0.405 (0.189-0.867) Preserved	0.530 (0.247-1.138) Attenuated
Infection composite	20 (10.4%)	0.748 (0.472-1.184) Unstable	0.800 (0.500-1.279) Unstable	0.872 (0.543-1.402) Unstable
Serious infection proxy	18 (9.3%)	1.083 (0.669-1.752) Attenuated	1.239 (0.756-2.031) Attenuated	1.351 (0.818-2.231) Attenuated
Pneumonia/lower respiratory infection	13 (6.7%)	1.772 (1.015-3.093) Preserved	1.506 (0.847-2.678) Attenuated	1.716 (0.959-3.071) Attenuated
COVID-19	8 (4.1%)	1.398 (0.700-2.793) Attenuated	0.998 (0.485-2.054) Unstable	1.329 (0.641-2.754) Attenuated
CMV	0 (0.0%)	0.408 (0.025-6.576) Unstable	Not estimable	Not estimable
Narrow opportunistic infection	3 (1.6%)	0.593 (0.205-1.711) Preserved	0.736 (0.232-2.330) Preserved	0.879 (0.274-2.820) Preserved
**D: Common calendar period 2024Q2-2026Q1 (analysis cohort N = 36,652)**				
**Epcoritamab (N = 1,272)**				
CRS	417 (32.8%)	6.596 (5.826-7.467) Preserved	4.871 (4.256-5.574) Preserved	9.032 (7.647-10.668) Preserved
ICANS	113 (8.9%)	3.140 (2.566-3.844) Preserved	2.622 (2.116-3.250) Preserved	8.248 (6.255-10.877) Preserved
Broadly defined neurotoxicity	167 (13.1%)	1.764 (1.493-2.085) Preserved	1.797 (1.509-2.140) Preserved	2.431 (2.009-2.943) Preserved
Infection composite	301 (23.7%)	1.383 (1.212-1.578) Preserved	1.264 (1.102-1.449) Unstable; NC	2.221 (1.912-2.580) Preserved
Serious infection proxy	237 (18.6%)	1.556 (1.347-1.798) Preserved	1.387 (1.193-1.612) Unstable; NC	2.264 (1.918-2.672) Preserved
Pneumonia/lower respiratory infection	82 (6.4%)	1.114 (0.887-1.398) Preserved	0.956 (0.755-1.209) Preserved	1.256 (0.975-1.618) Preserved
COVID-19	60 (4.7%)	1.153 (0.886-1.501) Preserved	1.150 (0.874-1.514) Preserved	2.728 (2.020-3.684) Unstable
CMV	111 (8.7%)	6.108 (4.941-7.549) Preserved	5.052 (3.997-6.387) Preserved	13.311 (9.845-17.996) Preserved
Narrow opportunistic infection	129 (10.1%)	2.165 (1.794-2.613) Preserved	2.105 (1.726-2.568) Preserved	4.376 (3.484-5.498) Preserved

Endpoint	Events, n (%)	Crude ROR (95% CI)	Primary aROR (95% CI)	Extended aROR (95% CI)
**Glofitamab (N = 739)**				
CRS	176 (23.8%)	3.887 (3.267-4.623)<br>Preserved	2.643 (2.206-3.166)<br>Preserved	5.723 (4.694-6.977)<br>Preserved
ICANS	40 (5.4%)	1.762 (1.277-2.432)<br>Attenuated	1.330 (0.956-1.848)<br>Unstable; NC	4.320 (3.021-6.179)<br>Attenuated
Broadly defined neurotoxicity	59 (8.0%)	0.993 (0.760-1.298)<br>Preserved	0.946 (0.721-1.242)<br>Preserved	1.405 (1.064-1.857)<br>Unstable
Infection composite	106 (14.3%)	0.736 (0.598-0.905)<br>Preserved	0.681 (0.552-0.840)<br>Unstable; NC	1.144 (0.923-1.419)<br>Unstable
Serious infection proxy	79 (10.7%)	0.799 (0.632-1.010)<br>Attenuated	0.711 (0.560-0.902)<br>Preserved	1.165 (0.913-1.487)<br>Unstable
Pneumonia/lower respiratory infection	39 (5.3%)	0.901 (0.652-1.245)<br>Preserved	0.815 (0.586-1.132)<br>Preserved	1.141 (0.816-1.596)<br>Preserved
COVID-19	25 (3.4%)	0.817 (0.549-1.217)<br>Preserved	0.918 (0.610-1.381)<br>Preserved	1.454 (0.959-2.203)<br>Preserved
CMV	12 (1.6%)	0.937 (0.533-1.648)<br>Preserved	0.668 (0.374-1.194)<br>Preserved	0.981 (0.542-1.773)<br>Preserved
Narrow opportunistic infection	21 (2.8%)	0.546 (0.355-0.840)<br>Preserved	0.471 (0.303-0.731)<br>Preserved	0.849 (0.543-1.326)<br>Attenuated
**Mosunetuzumab (N = 152)**				
CRS	38 (25.0%)	4.021 (2.786-5.805)<br>Preserved	7.634 (5.136-11.345)<br>Unstable; NC	8.407 (5.581-12.664)<br>Preserved
ICANS	2 (1.3%)	0.497 (0.142-1.737)<br>Unstable	0.742 (0.181-3.044)<br>Unstable	1.153 (0.280-4.755)<br>Unstable
Broadly defined neurotoxicity	7 (4.6%)	0.584 (0.280-1.219)<br>Attenuated	0.826 (0.383-1.781)<br>Attenuated	0.880 (0.408-1.901)<br>Attenuated
Infection composite	33 (21.7%)	1.235 (0.841-1.814)<br>Attenuated	1.163 (0.784-1.726)<br>Unstable; NC	1.541 (1.034-2.297)<br>Preserved
Serious infection proxy	23 (15.1%)	1.211 (0.779-1.881)<br>Attenuated	1.460 (0.927-2.299)<br>Attenuated	1.854 (1.172-2.932)<br>Preserved
Pneumonia/lower respiratory infection	10 (6.6%)	1.180 (0.630-2.213)<br>Attenuated	1.080 (0.562-2.075)<br>Attenuated	1.264 (0.655-2.437)<br>Attenuated
COVID-19	10 (6.6%)	1.699 (0.906-3.186)<br>Attenuated	0.847 (0.439-1.635)<br>Unstable	1.424 (0.734-2.761)<br>Attenuated
CMV	2 (1.3%)	0.907 (0.259-3.173)<br>Unstable	0.837 (0.203-3.451)<br>Unstable	0.991 (0.239-4.107)<br>Unstable
Narrow opportunistic infection	5 (3.3%)	0.686 (0.292-1.608)<br>Preserved	0.825 (0.334-2.036)<br>Preserved	1.123 (0.453-2.787)<br>Preserved

ROR, reporting odds ratio; aROR, adjusted reporting odds ratio; NC, model did not converge. Interpretation flags (Preserved, Attenuated, Unstable, and Not estimable) are retained from the source table and shown beneath the corresponding model estimate.

Supplementary Table S12.

Endpoint	Exposed events, n (%)	Comparator events, n (%)	Crude ROR (95% CI)	Primary aROR (95% CI)	Extended aROR (95% CI)
**Epcoritamab (exposed reports N = 1,463)**					
CRS	459 (31.4%)	3850 (6.3%)	6.750 (6.016-7.573)<br>Preserved	5.047 (4.452-5.721)<br>Preserved	12.563 (10.791-14.626)<br>Preserved
ICANS	119 (8.1%)	1740 (2.9%)	3.009 (2.481-3.650)<br>Preserved	2.292 (1.870-2.808)<br>Preserved	12.030 (9.247-15.651)<br>Preserved
Broadly defined neurotoxicity	185 (12.6%)	4728 (7.8%)	1.716 (1.467-2.008)<br>Preserved	1.696 (1.441-1.996)<br>Preserved	2.629 (2.207-3.131)<br>Preserved
Infection composite	374 (25.6%)	11666 (19.2%)	1.444 (1.282-1.626)<br>Preserved	1.346 (1.190-1.523)<br>Preserved	2.348 (2.058-2.679)<br>Preserved
Serious infection proxy	296 (20.2%)	7863 (13.0%)	1.705 (1.498-1.941)<br>Preserved	1.517 (1.326-1.735)<br>Preserved	2.522 (2.181-2.916)<br>Preserved
Pneumonia/lower respiratory infection	115 (7.9%)	3750 (6.2%)	1.300 (1.072-1.576)<br>Attenuated	1.199 (0.983-1.463)<br>Preserved	1.586 (1.284-1.960)<br>Attenuated
COVID-19	69 (4.7%)	3316 (5.5%)	0.862 (0.676-1.099)<br>Preserved	0.976 (0.759-1.256)<br>Preserved	1.777 (1.365-2.314)<br>Unstable
CMV	120 (8.2%)	918 (1.5%)	5.834 (4.789-7.108)<br>Preserved	4.531 (3.655-5.618)<br>Preserved	11.823 (9.115-15.334)<br>Preserved
Narrow opportunistic infection	153 (10.5%)	2891 (4.8%)	2.340 (1.972-2.778)<br>Preserved	2.174 (1.816-2.602)<br>Preserved	4.529 (3.710-5.528)<br>Preserved
**Glofitamab (exposed reports N = 944)**					
CRS	253 (26.8%)	3850 (6.3%)	5.409 (4.667-6.269)<br>Preserved	4.069 (3.484-4.753)<br>Preserved	14.897 (12.558-17.672)<br>Preserved
ICANS	47 (5.0%)	1740 (2.9%)	1.792 (1.333-2.408)<br>Attenuated	1.364 (1.008-1.846)<br>Attenuated	9.766 (6.928-13.766)<br>Attenuated
Broadly defined neurotoxicity	71 (7.5%)	4728 (7.8%)	0.968 (0.759-1.235)<br>Preserved	0.929 (0.726-1.189)<br>Preserved	1.668 (1.296-2.147)<br>Unstable
Infection composite	156 (16.5%)	11666 (19.2%)	0.834 (0.701-0.991)<br>Preserved	0.797 (0.669-0.950)<br>Preserved	1.235 (1.032-1.479)<br>Unstable
Serious infection proxy	113 (12.0%)	7863 (13.0%)	0.917 (0.752-1.117)<br>Attenuated	0.833 (0.682-1.017)<br>Attenuated	1.312 (1.069-1.611)<br>Unstable
Pneumonia/lower respiratory infection	58 (6.1%)	3750 (6.2%)	1.001 (0.767-1.307)<br>Preserved	0.984 (0.751-1.291)<br>Preserved	1.244 (0.945-1.638)<br>Preserved
COVID-19	44 (4.7%)	3316 (5.5%)	0.855 (0.631-1.157)<br>Preserved	1.066 (0.782-1.454)<br>Preserved	1.319 (0.963-1.807)<br>Preserved
CMV	17 (1.8%)	918 (1.5%)	1.227 (0.761-1.978)<br>Preserved	0.936 (0.574-1.527)<br>Preserved	1.922 (1.167-3.165)<br>Unstable
Narrow opportunistic infection	29 (3.1%)	2891 (4.8%)	0.644 (0.445-0.931)<br>Attenuated	0.560 (0.385-0.815)<br>Preserved	1.044 (0.714-1.525)<br>Unstable

Endpoint	Exposed events, n (%)	Comparator events, n (%)	Crude ROR (95% CI)	Primary aROR (95% CI)	Extended aROR (95% CI)
**Mosunetuzumab (exposed reports N = 472)**					
CRS	110 (23.3%)	3850 (6.3%)	4.498 (3.626-5.579)<br>Preserved	6.805 (5.421-8.543)<br>Preserved	11.495 (9.033-14.627)<br>Preserved
ICANS	4 (0.8%)	1740 (2.9%)	0.325 (0.128-0.824)<br>Attenuated	0.502 (0.186-1.352)<br>Preserved	1.579 (0.579-4.305)<br>Preserved
Broadly defined neurotoxicity	16 (3.4%)	4728 (7.8%)	0.428 (0.261-0.699)<br>Preserved	0.497 (0.300-0.822)<br>Preserved	0.582 (0.351-0.966)<br>Preserved
Infection composite	138 (29.2%)	11666 (19.2%)	1.739 (1.425-2.122)<br>Preserved	1.554 (1.267-1.905)<br>Preserved	2.102 (1.708-2.586)<br>Preserved
Serious infection proxy	96 (20.3%)	7863 (13.0%)	1.721 (1.374-2.155)<br>Preserved	1.682 (1.336-2.117)<br>Preserved	2.127 (1.681-2.690)<br>Preserved
Pneumonia/lower respiratory infection	59 (12.5%)	3750 (6.2%)	2.184 (1.661-2.871)<br>Preserved	1.761 (1.331-2.331)<br>Preserved	2.023 (1.519-2.694)<br>Preserved
COVID-19	60 (12.7%)	3316 (5.5%)	2.536 (1.932-3.329)<br>Preserved	1.662 (1.252-2.204)<br>Preserved	2.929 (2.191-3.916)<br>Preserved
CMV	9 (1.9%)	918 (1.5%)	1.333 (0.699-2.543)<br>Preserved	1.379 (0.704-2.700)<br>Preserved	2.872 (1.447-5.701)<br>Attenuated
Narrow opportunistic infection	20 (4.2%)	2891 (4.8%)	0.905 (0.581-1.411)<br>Preserved	0.949 (0.602-1.495)<br>Preserved	1.407 (0.887-2.231)<br>Preserved

ROR, reporting odds ratio; aROR, adjusted reporting odds ratio. Comparator reports N = 60,665 for all drug-specific analyses. Interpretation flags are retained from the source table and shown beneath the corresponding model estimate. All adjusted models converged.

Supplementary Table S13.

Characteristic	Valid TTO, n (%)	Without valid TTO, n (%)	Standardized difference
**Epcoritamab (valid TTO N = 613; without valid TTO N = 850)**			
**Age group**			
Age <65	142 (23.2%)	164 (19.3%)	0.095
Age 65-74	195 (31.8%)	162 (19.1%)	0.296
Age >=75	245 (40.0%)	303 (35.6%)	0.089
Age unknown	31 (5.1%)	221 (26.0%)	-0.604
**Sex**			
Female	247 (40.3%)	292 (34.4%)	0.123
Male	326 (53.2%)	460 (54.1%)	-0.019
Sex unknown	40 (6.5%)	98 (11.5%)	-0.175
**Reporting region**			
US	59 (9.6%)	116 (13.6%)	-0.126
Non-US	554 (90.4%)	734 (86.4%)	0.126
**B-cell lymphoma subgroup**			
LBCL/DLBCL/HGBCL	512 (83.5%)	743 (87.4%)	-0.110
FL	51 (8.3%)	49 (5.8%)	0.100
Other/unspecified B-cell lymphoma	50 (8.2%)	58 (6.8%)	0.051
**Report year**			
Report year 2025-2026	313 (51.1%)	540 (63.5%)	-0.254
**Outcome**			
Death	218 (35.6%)	333 (39.2%)	-0.075
Hospitalization	271 (44.2%)	172 (20.2%)	0.531
Severe outcome	436 (71.1%)	457 (53.8%)	0.364
**Selected event**			
CRS	201 (32.8%)	258 (30.4%)	0.052
ICANS	53 (8.6%)	66 (7.8%)	0.032
Infection composite	195 (31.8%)	179 (21.1%)	0.246

Characteristic	Valid TTO, n (%)	Without valid TTO, n (%)	Standardized difference
**Glofitamab (valid TTO N = 318; without valid TTO N = 626)**			
**Age group**			
Age <65	145 (45.6%)	196 (31.3%)	0.297
Age 65-74	96 (30.2%)	114 (18.2%)	0.282
Age >=75	64 (20.1%)	80 (12.8%)	0.199
Age unknown	13 (4.1%)	236 (37.7%)	-0.908
**Sex**			
Female	124 (39.0%)	198 (31.6%)	0.155
Male	192 (60.4%)	312 (49.8%)	0.213
Sex unknown	2 (0.6%)	116 (18.5%)	-0.638
**Reporting region**			
US	31 (9.7%)	91 (14.5%)	-0.147
Non-US	287 (90.3%)	535 (85.5%)	0.147
**B-cell lymphoma subgroup**			
LBCL/DLBCL/HGBCL	238 (74.8%)	541 (86.4%)	-0.296
FL	7 (2.2%)	12 (1.9%)	0.020
Other/unspecified B-cell lymphoma	73 (23.0%)	73 (11.7%)	0.302
**Report year**			
Report year 2025-2026	112 (35.2%)	350 (55.9%)	-0.425
**Outcome**			
Death	69 (21.7%)	265 (42.3%)	-0.454
Hospitalization	173 (54.4%)	103 (16.5%)	0.864
Severe outcome	237 (74.5%)	352 (56.2%)	0.392
**Selected event**			
CRS	98 (30.8%)	155 (24.8%)	0.136
ICANS	9 (2.8%)	38 (6.1%)	-0.158
Infection composite	54 (17.0%)	102 (16.3%)	0.018
**Mosunetuzumab (valid TTO N = 289; without valid TTO N = 183)**			
**Age group**			
Age <65	112 (38.8%)	42 (23.0%)	0.347
Age 65-74	89 (30.8%)	18 (9.8%)	0.540
Age >=75	80 (27.7%)	28 (15.3%)	0.305
Age unknown	8 (2.8%)	95 (51.9%)	-1.322

Characteristic	Valid TTO, n (%)	Without valid TTO, n (%)	Standardized difference
**Sex**			
Female	130 (45.0%)	65 (35.5%)	0.194
Male	155 (53.6%)	71 (38.8%)	0.301
Sex unknown	4 (1.4%)	47 (25.7%)	-0.760
**Reporting region**			
US	93 (32.2%)	100 (54.6%)	-0.465
Non-US	196 (67.8%)	83 (45.4%)	0.465
**B-cell lymphoma subgroup**			
LBCL/DLBCL/HGBCL	28 (9.7%)	10 (5.5%)	0.160
FL	78 (27.0%)	158 (86.3%)	-1.495
Other/unspecified B-cell lymphoma	183 (63.3%)	15 (8.2%)	1.406
**Report year**			
Report year 2025-2026	24 (8.3%)	80 (43.7%)	-0.882
**Outcome**			
Death	38 (13.1%)	20 (10.9%)	0.068
Hospitalization	240 (83.0%)	42 (23.0%)	1.508
Severe outcome	254 (87.9%)	58 (31.7%)	1.398
**Selected event**			
CRS	59 (20.4%)	51 (27.9%)	-0.175
ICANS	2 (0.7%)	2 (1.1%)	-0.043
Infection composite	107 (37.0%)	31 (16.9%)	0.465
